# Prognostic risk model of LIHC T-cells based on scRNA-seq and RNA-seq and the regulation of the tumor immune microenvironment

**DOI:** 10.1007/s12672-024-01424-z

**Published:** 2024-10-10

**Authors:** Shoupeng Ding, Xiaomei Yi, Jinghua Gao, Chunxiao Huang, Shouzhao Zheng, Lixian Wu, Zihan Cai

**Affiliations:** 1 Department of Laboratory Medicine, Gutian County Hospital, Gutian, 352200 China; 2 Department of Medical Laboratory, Siyang Hospital, Siyang, 237000 China; 3 Department of Microbiology and Immunology, School of Basic Medical Sciences, Dali University, No. 22, Wanhua Road, Xiaguan Town, Dali, 671000 China; 4 Department of Laboratory Medicine, Ninghua County General Hospital, Ninghua, 365400 China; 5 Chuxiong Yi Autonomous Prefecture People’s Hospital, Chuxiong, 675000 China

**Keywords:** scRNA, Immune cells, TME, T cell maturation trajectory, Prognostic risk modeling

## Abstract

**Background:**

T-cell-related genes play a crucial role in LIHC development. However, a reliable prognostic profile based on risk models of these genes has yet to be identified.

**Methods:**

Single-cell datasets from both tumor and normal tissue samples were obtained from the GEO database. We identified T-cell marker genes and developed a genetic risk model using the TCGA-LIHC dataset, which was subsequently validated with an independent GEO dataset. We also explored the relationship between risk model predictions and immune responses.

**Results:**

We constructed a prognostic risk model using eight gene features identified through screening 860 T-cell marker genes via scRNA-seq and RNA-seq, which was subsequently integrated with the TCGA dataset. Its validity was independently confirmed using GEO and ICGC datasets. The TCGA dataset was stratified into high-risk and low-risk groups based on the risk model. Multivariate Cox regression analysis confirmed the risk score as an independent prognostic factor. GSEA indicated ribosomal transporter metabolism enrichment in the high-risk group and significant transcriptional activation in the low-risk group. ESTIMATE analysis showed higher ESTIMATE, immune, and stromal scores in the low-risk group, which also exhibited lower tumor purity than the high-risk group. Immunophenotyping revealed distinct patterns of immune cell infiltration and an immunosuppressive environment in the high-risk group.

**Conclusions:**

This study introduces a T-cell marker-based prognostic risk model for LIHC patients. This model effectively predicted survival outcomes and immunotherapy effectiveness in LIHC patients, aligning with diverse immune responses and the distinct immunological profiles observed in the high-risk group.

**Supplementary Information:**

The online version contains supplementary material available at 10.1007/s12672-024-01424-z.

## Introduction

Hepatocellular carcinoma (LIHC), the seventh most common cancer globally, is a prevalent malignancy characterized by high recurrence, late detection, and poor prognosis [1]. Despite the revolutionary impact of cancer immunotherapy on tumor treatment, the effectiveness of emerging immune checkpoint blockade therapies varies significantly among patients with different cancers and cancer subtypes [2]. Tumorigenesis and progression are influenced not only by the clinical stage but also by aberrant gene expression. Therefore, identifying a biomarker that predicts therapeutic efficacy is crucial.

Immunotherapy, successful in treating various tumors, plays a key role in both the formation and progression of the Tumor Microenvironment (TME). The effectiveness of immunosuppressants partly depends on their ability to activate CD8 + T cells within the TME, but this function can be impeded by T cell exhaustion, often induced by regulatory T cells [3]. For example, research by Bonnal et al. revealed significant heterogeneity in regulatory T cells during immune infiltration in colon and lung cancers [4]. In the intricate landscape of non-small cell lung cancer (NSCLC), tumor-infiltrating effector T cell subsets emerge as potent mediators of anti-tumor immunity, characterized by their robust expression of cytokines such as IFN-γ and TNF-α, which are pivotal in orchestrating immune responses against malignancies. These effector T cells, endowed with formidable cytotoxic capabilities, not only exhibit a pronounced ability to eradicate tumor cells but also demonstrate a significant correlation with enhanced patient survival outcomes [5]. Conversely, T cell depletion presents a formidable challenge; however, interventions such as PD-1/PD-L1 blockade therapy offer a glimmer of hope by partially rejuvenating these exhausted T cells, thereby amplifying the anti-tumor immune response [6, 7]. Intriguingly, the extent of T cell depletion has been found to correlate with the therapeutic efficacy of PD-1 blockade, positioning the depleted T cell status as a potential predictive marker for treatment responsiveness. Collectively, these insights underscore the critical importance of unraveling the molecular intricacies and heterogeneity of T cells within the tumor microenvironment (TME), a pursuit that holds the key to devising more nuanced and effective strategies in the realm of cancer immunotherapy.

In recent years, numerous studies using scRNA data have sought potential prognostic markers for LIHC, advancing our knowledge of tumor immunology [8]. Cutting-edge single cell sequencing technology (SCT) enables gene expression analysis at the individual cell level, allowing for the examination of cell types, tumor heterogeneity and their clinical relevance in the TME. This single-cell analysis provides a more detailed understanding of immune cells within the complex TME. The pivotal roles of T-cell exhaustion and activation in melanoma have become apparent [9].

**Fig. 1:**
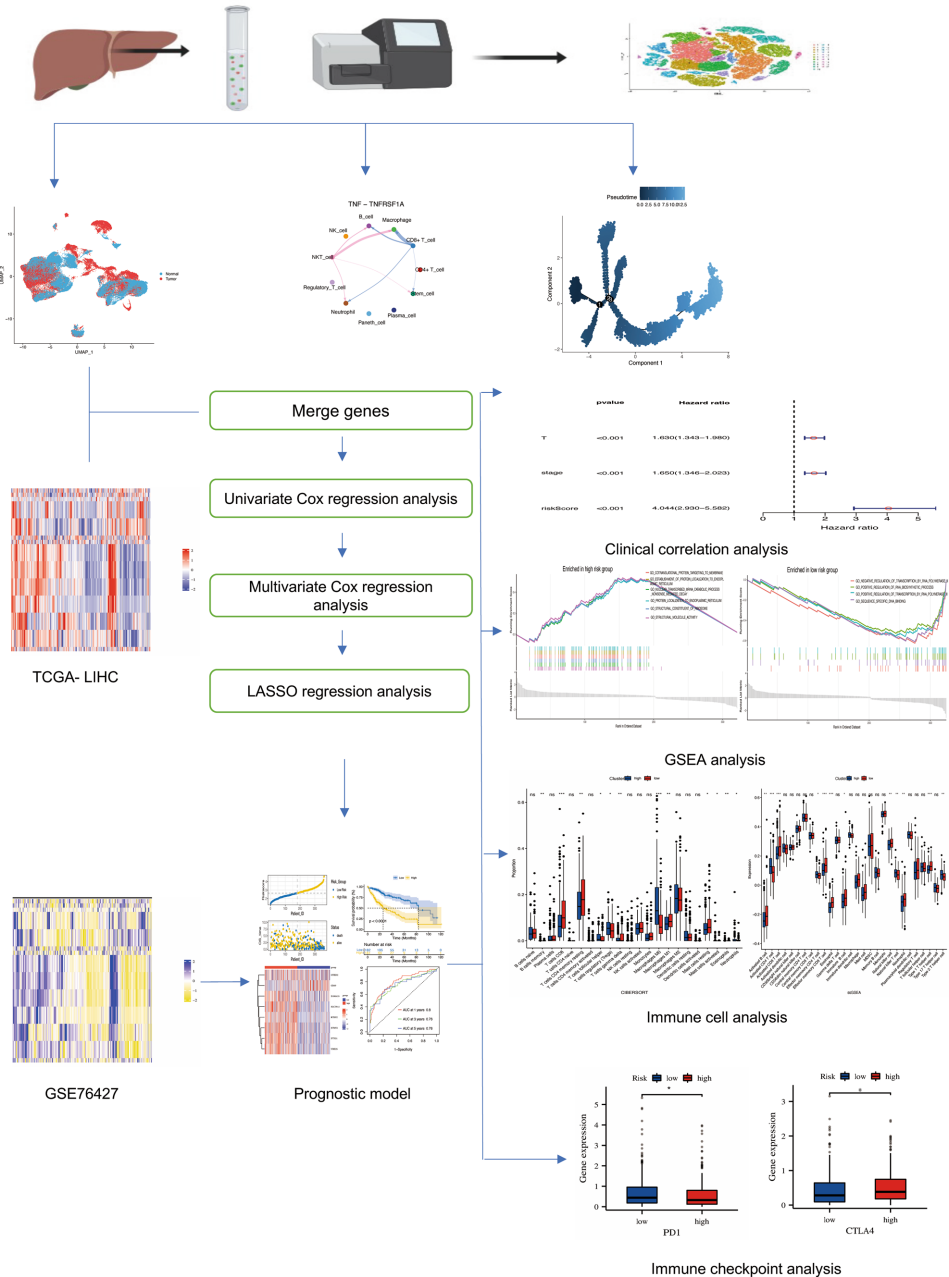
Experimental flow

While immunotherapy has advanced significantly, the limited range of treatment options highlights the need for in-depth research on the tumor immune microenvironment to discover new therapeutic strategies. Our study aims to integrate single-cell RNA sequencing (scRNA-seq) with bulk RNA sequencing data to uncover the cellular and molecular dynamics in hepatocellular carcinoma. Our objective is to develop and externally validate a robust single-cell data-driven prognostic model for LIHC patients. Figure 1 depicts the complete experimental procedure.

## Materials and methods

### Single-cell data acquisition and preprocessing

We obtained single cell sequencing data from the GSE202642 dataset in the GEO database, comprising 7 hepatocellular carcinoma cases and 4 adjacent liver tissue samples, processed using the 10 × Genomics platform. To ensure data quality, preprocessing was conducted using the Seurat [10] package in R version 4.3.1. This included removing low-quality cells (with fewer than 300 expressed genes) and rare genes (detected in fewer than 3 cells), resulting in 88,542 cells retained for analysis. Data normalization was achieved using the SCTransform method. For more efficient biological interpretation and analysis, Principal Component Analysis (PCA) was utilized for dimensionality reduction. Cell clustering was conducted using the UMAP technique, setting the resolution parameter at 0.4, which allowed the identification of 24 distinct cell populations based on specific epithelial and immune cell markers.

### Cell annotation

Cell grouping was performed in Seurat using the 'RunPCA' function, which reduces data dimensionality by focusing on the initial 2,000 highly variable genes. The 'FindNeighbors' and 'FindClusters' functions were then applied with a resolution parameter of 0.4, to improve clustering quality. Annotations were refined using CellMarker [11], the SingleR [12] package, and literature insights, enabling the prediction of cell-to-reference dataset correlations and the attainment of annotation data for the predicted cell types.

### Functional enrichment analysis of T cell marker genes

Marker genes were identified using the "FindAllMarkers" function in R, with a log-fold change (logfc) threshold of 0.25 and a minimum percentage (minpct) of 0.25. For each subgroup, the top 200 most highly expressed genes were selected for enrichment analysis. This was performed using the "ClusterProfiler" package [13] in R to graphically represent biological functions.

In addition, the specific parameters for GO enrichment analysis were (P = 0.05, pvalueCutoff = 0.01, qvalueCutoff = 0.05) and for KEGG enrichment analysis were (P = 0.05, pvalueCutoff = 0.01, qvalueCutoff = 0.05).

### Cell communication analysis

The CellChat package [14] was employed to analyze the intercellular communication network. Simultaneously, the patchwork package integrated expression matrices for potential ligand-receptor pair identification. This integration facilitated communication probability estimation and signaling pathway-level intercellular communication inference.

### Pseudo-temporal analysis of cell sequences

T cells underwent pseudo-temporal sequencing analysis, which categorized them into subsets identified in previous analyses. This aimed to explore cell expression changes over time. The Monocle3 [15] package in R was used to generate single-cell pseudo-temporal traces, which were subsequently enriched for further analysis.

### TCGA-LIHC data pre-processing and analysis

Bulk RNA-seq data, including 463 LIHC samples with corresponding clinical information, were obtained from the TCGA database (https://xenabrowser.net/datapages/?cohort=GDC%20TCGA%20Liver%20Cancer%20(LIHC)&removeHub=https%3A%2F%2Fxena.treehouse.gi.ucsc.edu%3A443). For risk model external validation, we used the GSE76427 dataset(https://www.ncbi.nlm.nih.gov/geo/query/acc.cgi?acc=GSE76427) from the GEO database, containing 115 LIHC and 52 non-hepatocellular carcinoma control samples. In addition, we used the ICGC dataset(https://docs.icgc-argo.org/docs/data-access/icgc-25k-data) for validation. Highly expressed genes in the TCGA-LIHC dataset were screened and aligned with T-cell marker genes from single-cell data to create a corresponding gene expression matrix.

### Prognostic model construction and survival analysis

Initially, prognostic differences in genes were identified using univariate Cox regression analysis. This was followed by LASSO regression analysis to pinpoint genes with high correlation, focusing on those with a P-value below 0.01. A multifactorial Cox regression analysis was then applied to these genes to develop predictive models, based on the formula:

$${\mathbf{Risk}} {\mathbf{Score}} = \mathop \sum \limits_{{{\mathbf{i}} = 0}}^{{\mathbf{k}}} {\mathbf{\beta i}}*{\mathbf{expi}}^{1}$$

In this formula, "βi" represents the coefficient of the selected genes from the multivariate Cox analysis, and " expi" refers to their expression levels. Patients were stratified into high-risk and low-risk groups based on the median risk score. Survival disparities and prognostic conditions of the patients were analyzed using the R packages "ggplot2" and "timeROC". The prognostic model was validated using the GSE76427 dataset as an independent external source.

### Clinical correlation analysis

We then examined the correlation between risk scores and the clinical characteristics of LIHC patients. The prognostic value of the risk scores was validated through initial univariate and multivariate Cox regression analyses. Prognostic nomograms incorporating N, T, M staging, pathological grade, and gender were constructed using the “rms” R package. Kaplan–Meier curves and Cox models were utilized for survival analysis validation. Gene Set Enrichment Analysis (GSEA) based on screening criteria (p < 0.05 and q < 0.25) revealed biological pathway differences between risk groups.

### Immune infiltration analysis

The TME in patients classified into high- and low-risk groups was analyzed using the CIBERSORT [16], ssGSEA [17], and ESTIMATE R packages. The study also explored the association between risk scores and immune checkpoint gene expression levels.

### RT-qPCR validation of 8 independent prognostic DEGs

Human normal hepatocyte LO2 cells and hepatocellular carcinoma cell lines were used in this experiment. Cells were cultured in Dulbecco's Modified Eagle Medium (DMEM) supplemented with 10% fetal bovine serum (FBS).

Total RNA from both normal human hepatocytes and hepatocellular carcinoma cells was isolated using a Total RNA Extraction Kit, following the manufacturer's protocol. Reverse transcription was performed using a cDNA First Strand Synthesis Kit. Real-time fluorescence quantitative PCR was performed using the Step One qPCR detection system. Table 1 lists the qPCR primers utilized.

**Table 1:** Primer sequences for real-time quantitative PCR

Gene	Primer (5'-3')	
*β-actin*	F: GGCTGTATTCCCCTCCATCG	R: CCAGTTGGTAACAATGCCATGT
*PTTG1*	F: ACCCGTGTGGTTGCTAAGG	R: ACGTGGTGTTGAAACTTGAGAT
*STMN1*	F: TCAGCCCTCGGTCAAAAGAAT	R: TTCTCGTGCTCTCGTTTCTCA
*UBE2S*	F: ACAAGGAGGTGACGACACTGA	R: CCACGTTCGGGTGGAAGAT
*RTKN2*	F: ATGCTCGACTAATGGCCTATACA	R: CGTCGTGATCGTTCTTTATTGCT
*S100A10*	F: GGCTACTTAACAAAGGAGGACC	R: GAGGCCCGCAATTAGGGAAA
*CITED2*	F: CCTAATGGGCGAGCACATACA	R: GGGGTAGGGGTGATGGTTGA
*SLC38A1*	F: TGACAGTGCCCGAGGATGATA	R: AGACATGCCTAAGGAGGTTGTA
*CD69*	F: ATTGTCCAGGCCAATACACATT	R: CCTCTCTACCTGCGTATCGTTTT

### Statistical analysis

The association between continuous variables in the two groups was analyzed using the nonparametric Wilcoxon rank sum test. LASSO and Cox regression analyses were employed to construct the predictive model. Kaplan–Meier survival analysis and the log-rank test were used to compare survival differences among the different risk groups. A two-sided p-value of less than 0.05 was considered statistically significant. All analyses were performed using R software (version 4.3.1).

## Results

### Single-cell analysis of hepatocellular liver cancer

**Fig. 2:**
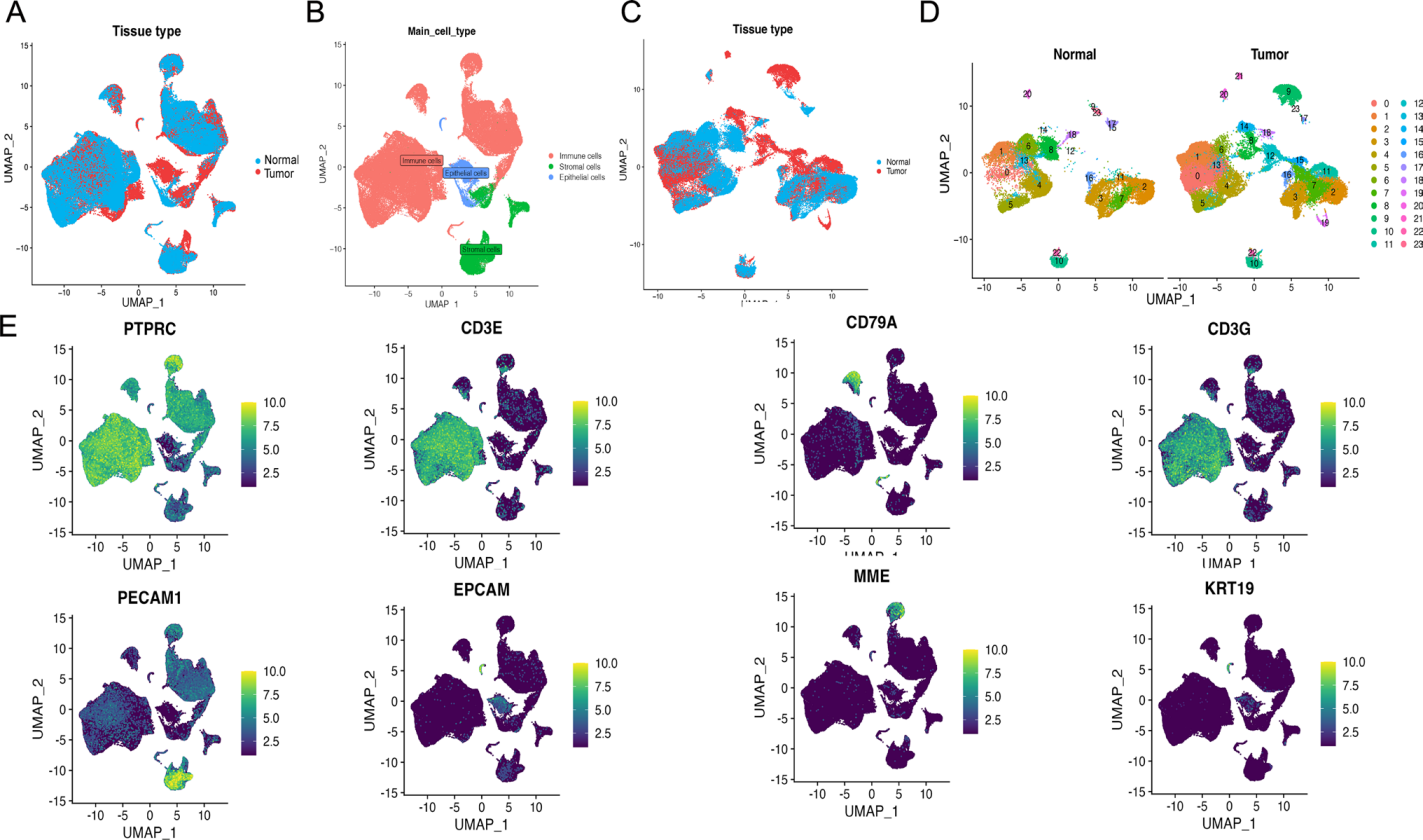
Construction of a single-cell atlas. **A** UMAP representation of normal and tumor samples; **B** UMAP visualization of immune cells and other cell types; **C** UMAP depiction of normal and tumor samples post-extraction of immune cells; **D** UMAP diagram of 24 distinct cell populations; **E** Map depicting the characterization of marker gene expression in cell populations

**Fig. 3:**
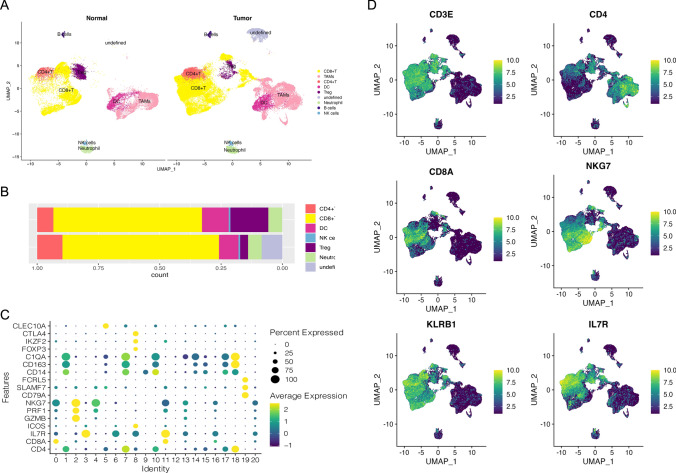
Construction of an immune cell atlas. **A** UMAP plots representing 8 cell populations; **B** Cell scale plots depicting relative proportions; **C** Characterization of genes expressed in cell populations; **D** Analysis of genes expressed in T cell populations

The analysis of eleven samples, including seven hepatocellular carcinoma tissues and four normal liver controls, revealed critical insights into the cellular landscape of the tumor microenvironment. Following rigorous quality control, normalization, batch correction, and PCA downsizing, single-cell dataset analysis distinguished tumor and control samples into epithelial, immune, and stromal cell populations (Fig. 2A, B). Notably, immune cells were classified into 24 distinct subpopulations (Fig. 2C, D). Key markers, such as PTPRC, CD3E, CD4, CD79A, and CD3G in immune cells, EPCAM and KRT19 in epithelial cells, and MME and PECAM1 in stromal cells (Fig. 2E), were identified, providing crucial insights into hepatocellular carcinoma progression and immune response mechanisms [18, 19]. The UMAP visualization further elucidated the diversity of cellular subpopulations, highlighting the presence of CD4 + T cells and TAMs (Fig. 3A–D), which are pivotal in understanding the immune microenvironment of hepatocellular carcinoma and informing therapeutic development strategies. In a further refinement of our analysis, we meticulously re-clustered and subclustered the CD4 + and CD8 + T cell populations, revealing a striking imbalance within the tumor microenvironment: a conspicuously elevated presence of effector T cells juxtaposed against a markedly diminished proportion of exhausted T cells (Figure SF1). "These findings offer a unique insight into immune regulation within the tumor microenvironment.

### Enrichment analysis of T-cell-related genes

**Fig. 4:**
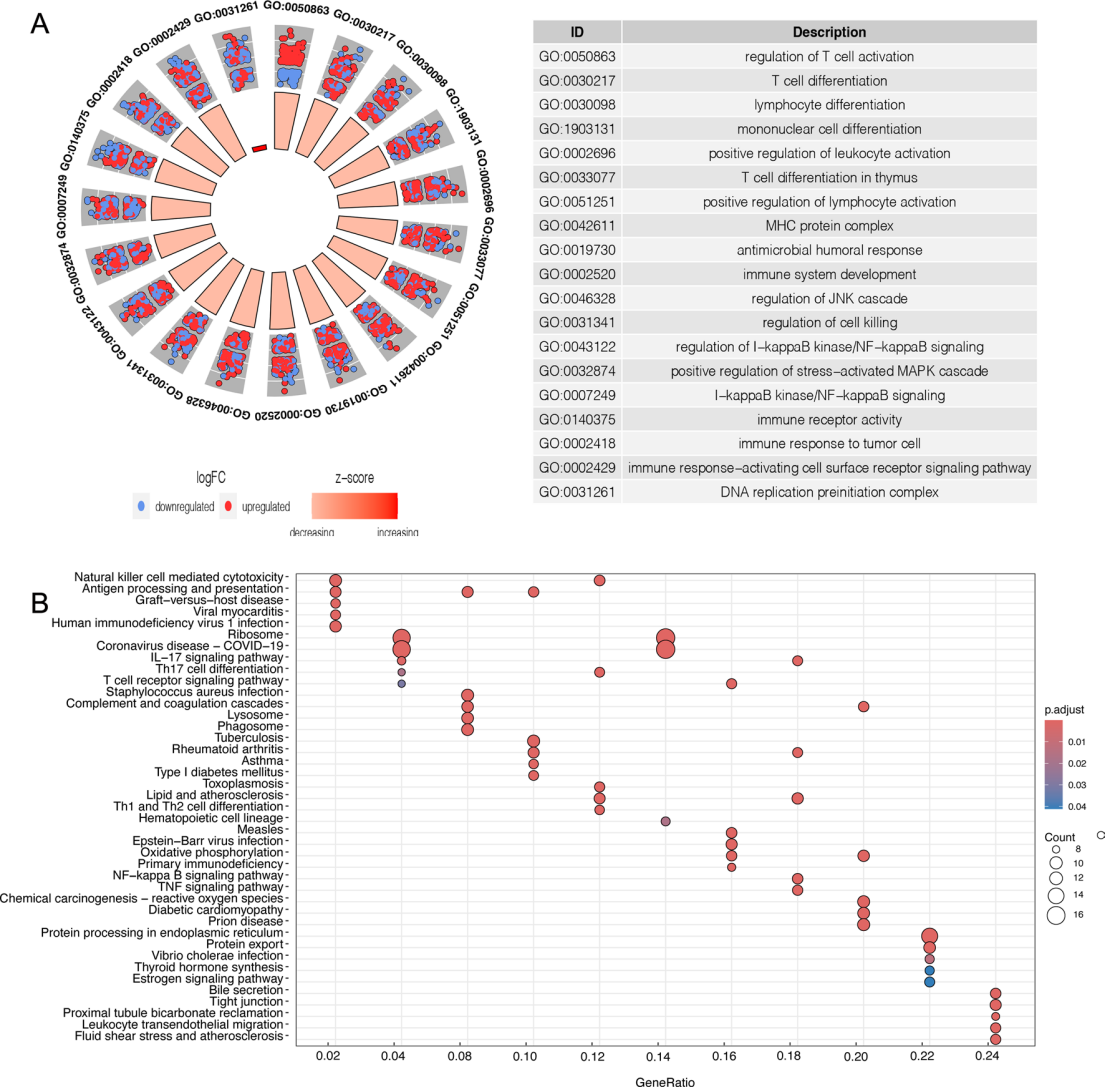
T cell enrichment analysis. **A** GO Enrichment analysis; **B** KEGG pathway enrichment analysis

GO and KEGG enrichment analysis of highly expressed genes in T-cell subpopulations revealed their pivotal roles in T-cell activation, immune response modulation, and ribosome biogenesis. These genes were primarily localized in ribosomes and mitochondria, engaging in molecular functions such as structural support, enzyme inhibition, and death receptor activation. This pattern suggests an increase in metabolic activity and enhanced immunosurveillance within these T cells. Additionally, KEGG analysis underscored the importance of cytotoxicity, apoptosis, and the PD-1/PD-L1 pathway, providing crucial insights into the activation of T-cell signaling pathways relevant to liver cancer therapy and the regulation of immune checkpoints (Fig. 4A, B and Supplementary Table 1–2).

### Analysis of T-cell-related inter-cellular interactions in scRNA-seq

**Fig. 5:**
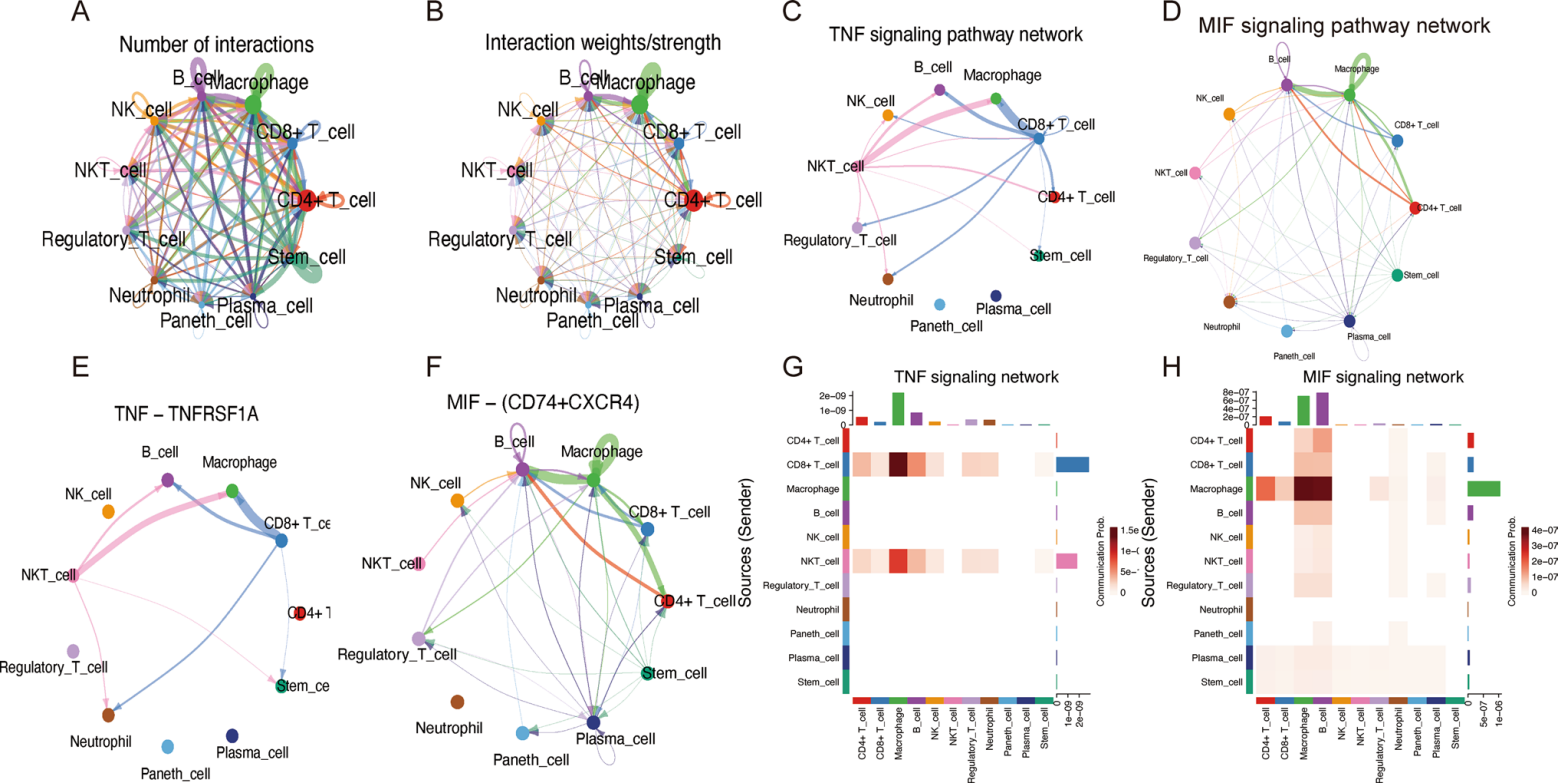
Analysis of Cell–Cell Communication. **A**, **B** Analysis of cell-to-cell interactions; **C** Identification of interacting cells in the TNF signaling pathway; **D** Identification of interacting cells in the MIF signaling pathway; **E** Analysis reveals TNF-TNFRSF1A's crucial role in the cell–cell communication network; **F** Analysis reveals MIF-(CD74 + CXCR4)'s crucial role in the cell–cell communication network; **G** Diverse cellular interactions within the TNF pathway; **H** Diverse cellular interactions within the MIF pathway

Investigating receptor-ligand interactions on cell surfaces offers crucial insights into the mechanisms of intercellular communication. By analyzing gene expression data within the expression matrix, we can infer protein expression and map out cellular interaction networks. Using the "CellChat" R package [14], we identified key communication networks within specific signaling pathways, with a particular emphasis on the significant roles of TNFRSF1A and MIF-(CD74 + CXCR4) in the TNF and MIF signaling pathways (Fig. 5A–H). These findings highlight the critical impact of these interactions on modulating cellular responses and illuminate potential therapeutic targets.

### T cell maturation trajectory analysis

**Fig. 6:**
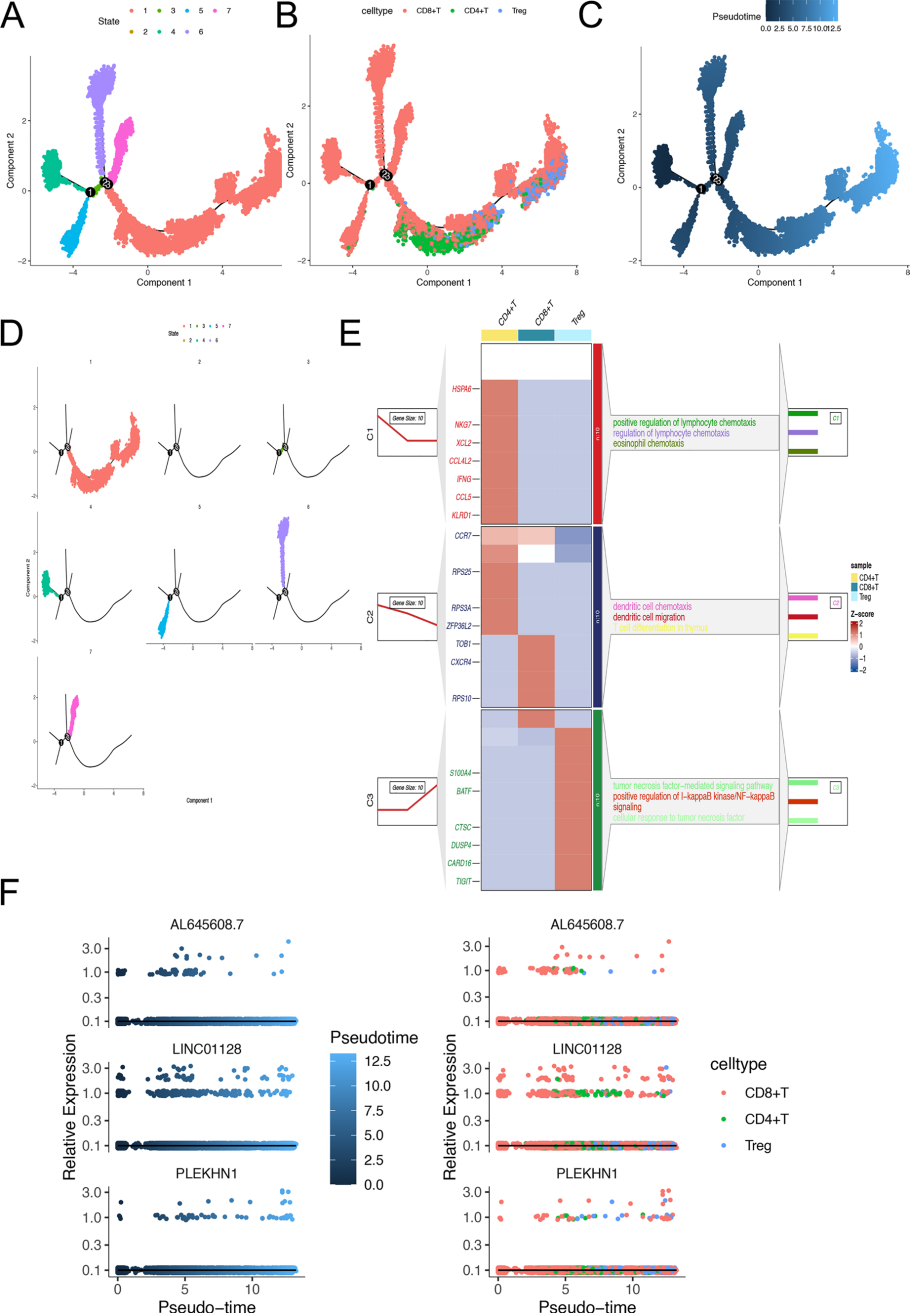
T cell temporal trajectory analysis. **A** Distribution of T cells in various states along temporal trajectories; **B** Distribution of diverse T cell types along temporal trajectories; **C** Representation of T cell developmental time; **D** Distribution of T cell trajectories across various states; **E** Analysis of differential gene enrichment; **F** Temporal changes in expression values of top 3 genes

Our pseudo-temporal analysis using Monocle3 identified seven distinct differentiation states within T-cell subpopulations, each crucial for understanding T-cell development (Fig. 6A, B). Notably, State 4 represents the initial stage of differentiation, as highlighted by the varying shades of color markers denoting different developmental stages (Fig. 6C, D). Enrichment analysis further revealed that key biological processes, including the regulation of cell chemotaxis, nucleotide metabolism, signaling pathways, cytoplasmic protein synthesis, and the TNF signaling pathway, are essential for T-cell functional differentiation and maturation (Fig. 6E). Additionally, Fig. 6F illustrates the differentiation states of three highly expressed genes across various cell types, underscoring their significance in T-cell development.

### Prognostic risk model construction and validation

**Fig. 7:**
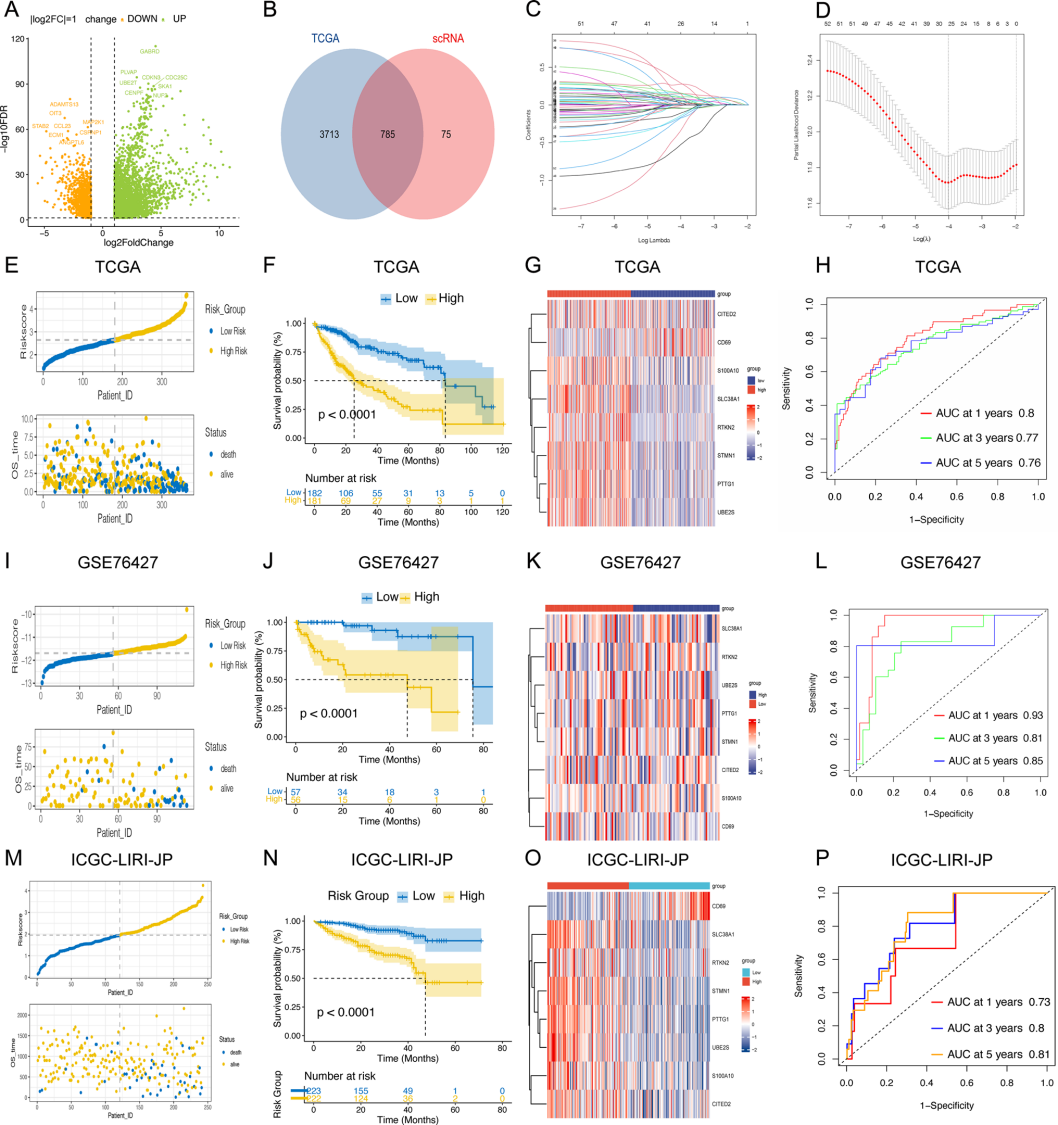
Prognostic risk model construction and validation. **A** Volcano plot analysis; **B** Wayne plot analysis; **C** LASSO Regression analysis; **D** Cross-validation for parameter optimization in LASSO regression; **E** Analysis of TCGA-LIHC risk scores; **F** Survival analysis of TCGA-LIHC patients based on risk scores; **G** Prognostic gene heatmap in TCGA-LIHC; **H** 1-, 3-, and 5-year survival analysis of TCGA-LIHC patients; **I** Analysis of GSE76427 Risk Scores; **J** Survival analysis of GSE76427 patients; **K** Prognostic gene heatmap in GSE76427; **L** 1-, 3-, and 5-year survival analysis of GSE76427 LIHC patients; **M**–**P** ICGC dataset validation of prognostic risk models

In developing the prognostic risk model, we identified highly expressed genes in the TCGA-LIHC dataset and validated them against T-cell marker genes from the single-cell dataset, uncovering potential prognostic markers (Fig. 7A, B). One-way Cox regression analysis revealed 730 genes significantly correlated with patient prognosis (P < 0.001) (Supplementary Table 3). Further refinement through LASSO regression isolated 52 key prognostic genes (Fig. 7C, D) (Supplementary Table 4). Multifactorial Cox regression analysis then identified eight independent prognostic DEGs: PTTG1, STMN1, UBE2S, RTKN2, S100A10, CITED2, SLC38A1, and CD69 (Supplementary Table 5). Stratifying patients into high and low-risk groups based on median risk scores revealed that the low-risk group had significantly better overall survival (OS) compared to the high-risk group (Fig. 7E–G). The model's predictive efficacy was confirmed by AUC values of 0.8, 0.76, and 0.76 for 1, 3, and 5-year OS in the TCGA cohort, respectively (Fig. 7H). These findings were consistently validated in the GSE76427 and ICGC datasets (Fig. 7I–P), demonstrating the model's robust ability to predict overall survival at 1, 3, and 5 years.

**Fig. 8:**
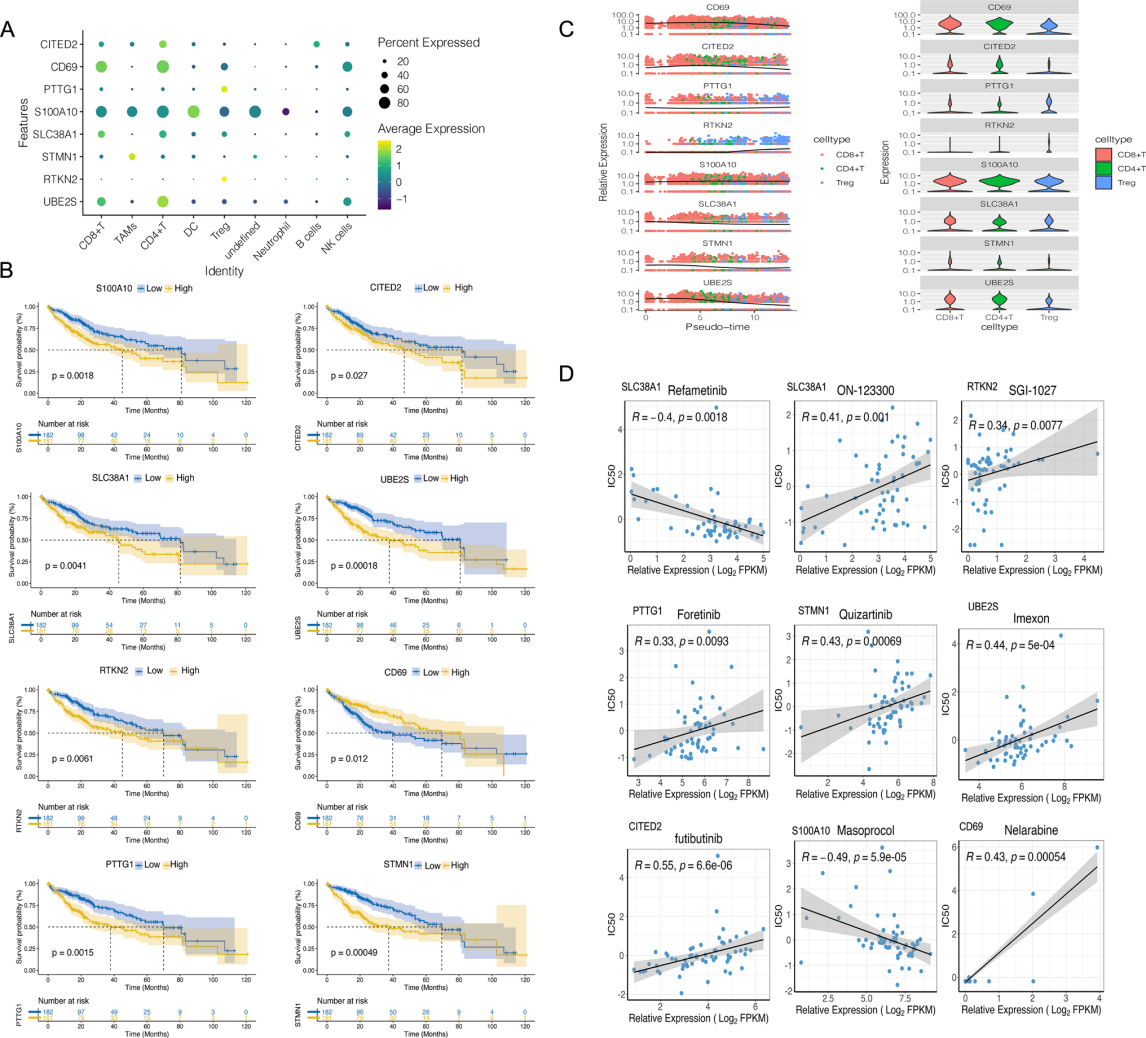
Expression analysis of prognostic genes in cellular subpopulations. **A** Analysis of prognostic gene expression in cellular populations; **B** Survival analysis based on prognostic genes; **C** Cell trajectory analysis of prognostic genes in T-cell populations; **D** Drug sensitivity analysis for prognostic genes

Figure 8A illustrates the expression patterns of eight independent prognostic DEGs within the single-cell dataset, while Fig. 8C's scatter plot depicts their dynamic expression trends across pseudo-time. Notably, seven of these prognostic genes, with the exception of CD69, were linked to higher survival rates in the low-risk group compared to the high-risk group (Fig. 8B). To improve HCC patient prognosis, the drug-gene interaction database CallMiner was utilized to identify potential drug candidates targeting these prognostic genes. Figure 8D showcases these candidates, selected for their therapeutic relevance in targeting the identified prognostic genes, which are pivotal for enhancing HCC patient outcomes. Further in vitro and in vivo studies are essential to validate these drug candidates and evaluate their impact on HCC prognosis, safety, and efficacy.

### Clinical correlation and enrichment analysis between high and low-risk groups

**Fig. 9:**
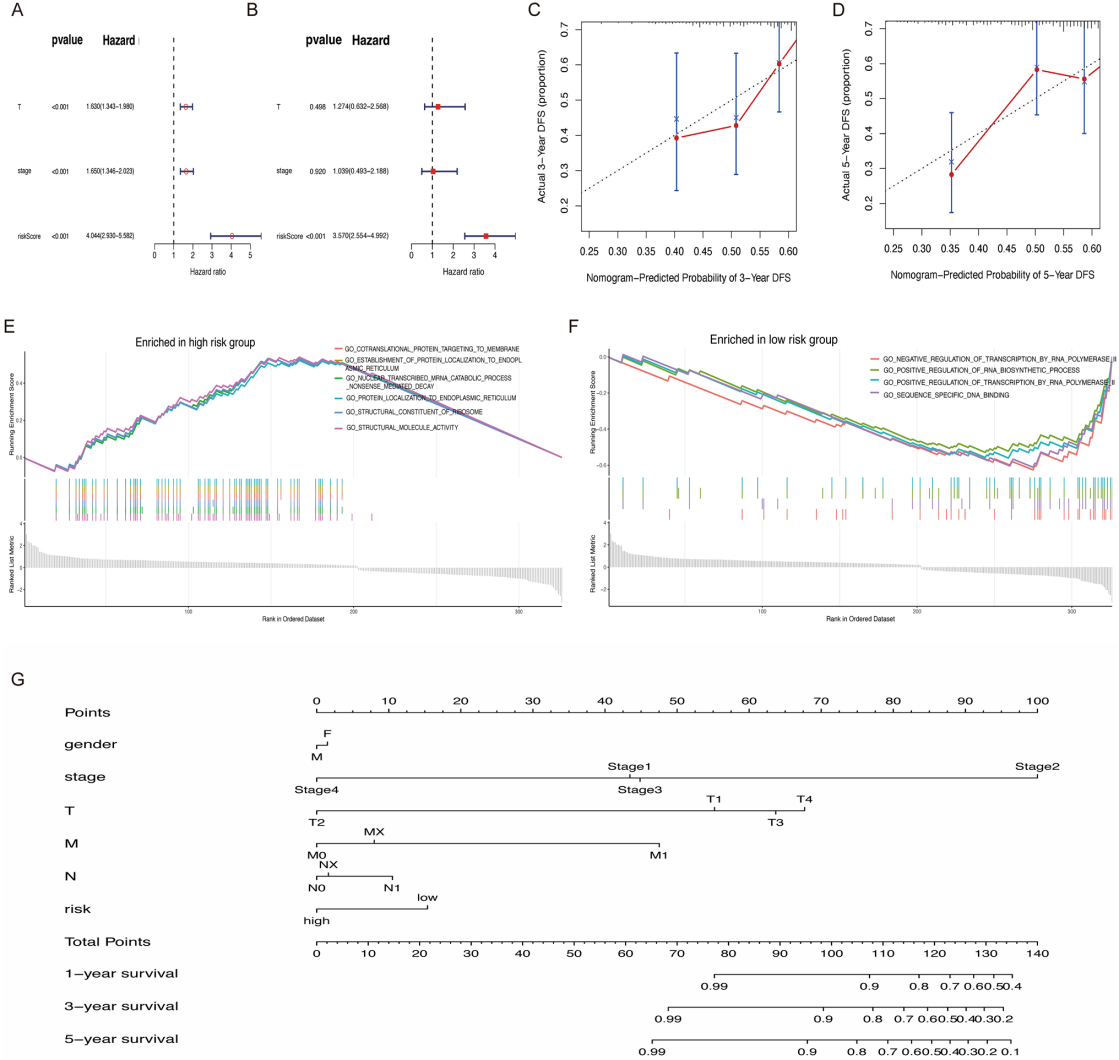
Clinical correlation analysis. **A**, **B** Single and multifactorial cox regression analyses; **C**, **D** 3- and 5-year survival analysis using column line plots; **E**, **F** GSEA enrichment analysis for high and low-risk groups; **G** Normalgram analysis

Cox regression analyses confirmed that the risk score is a significant independent predictor of overall survival (OS), showing a strong positive correlation (P < 0.001) (Fig. 9A, B). The risk score was validated as a robust prognostic factor. A nomogram created using the R package, accurately predicted OS by incorporating variables such as age, gender, stage, T, N, M, and risk score (Fig. 9G). Calibration curves further validated the nomogram’s accuracy in estimating 3- and 5-year survival probabilities (Fig. 9C, D). Moreover, GSEA identified distinct biological pathways between the groups, with ribosomal translocation metabolism emerging as a key process in the high-risk group (Fig. 9E) and significant enrichment of genomic transcriptional positive regulation in the low-risk group (Fig. 9F). These findings highlight the critical role of the risk score in stratifying patient prognosis and informing therapeutic decisions.

### Immune infiltration analysis

**Fig. 10:**
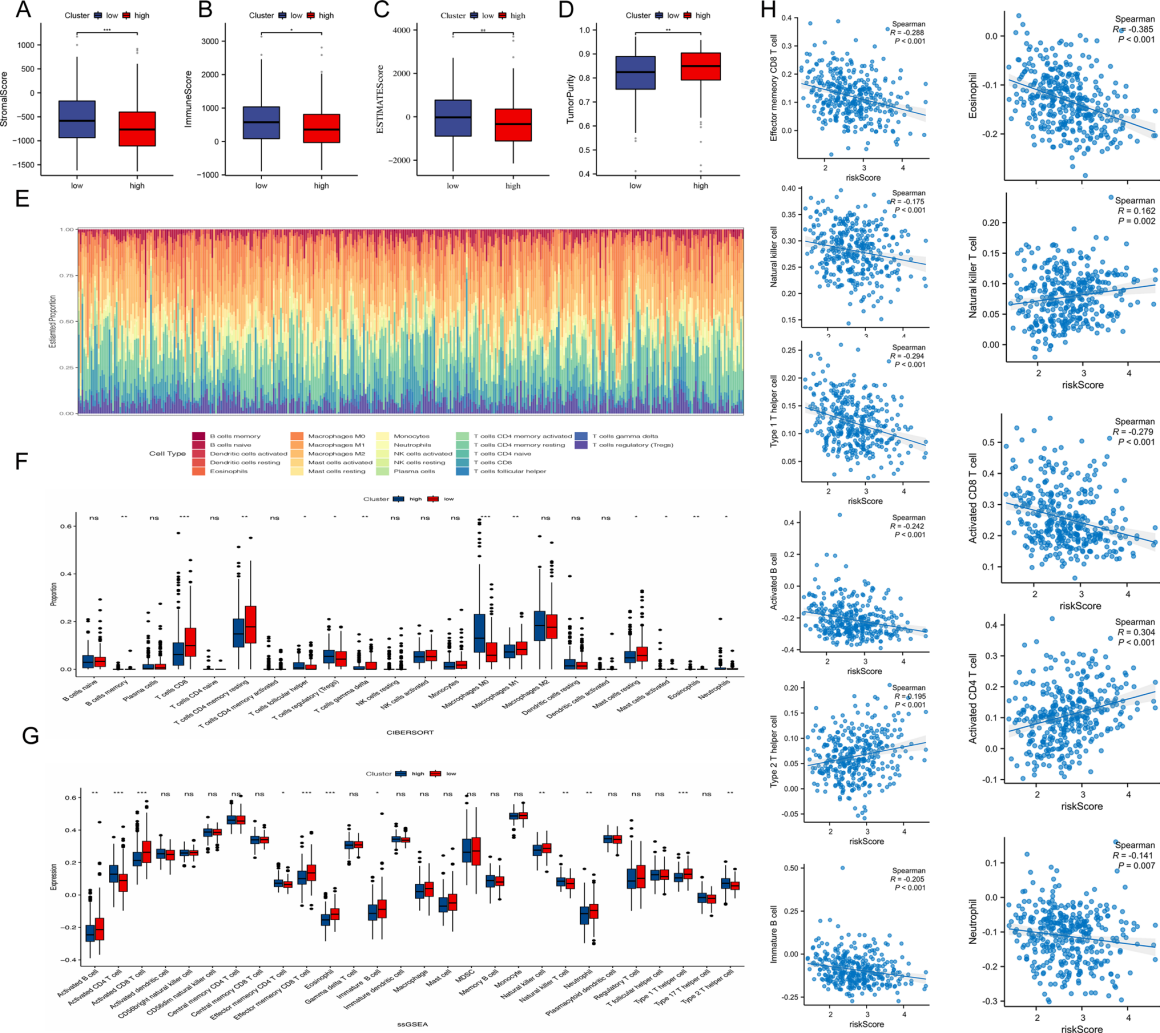
Immune infiltration analysis. **A**–**D** Analysis of ESTIMATE scores; **E** Analysis of immune cell abundance; **F** CIBERSORT analysis; **G** ssGSEA analysis; **H** Correlation analysis

Assessment of the tumor microenvironment using the R package ESTIMATE revealed significant differences between the low- and high-risk groups. The low-risk group exhibited higher immune cell and stromal cell ratios, elevated ESTIMATE scores, and reduced tumor purity (Fig. 10A–D). These findings suggest that the high-risk group is more susceptible to immune escape, potentially leading to increased tumor progression. This conclusion is further supported by the CIBERSORT analysis of 22 tumor-infiltrating immune cells, which showed significantly higher levels of memory B cells, follicular helper T cells, M0 macrophages, regulatory T cells, and neutrophils in the low-risk group. In contrast, the high-risk group had elevated levels of CD8 + T cells, resting CD4 memory T cells, M1 macrophages, and both resting and activated mast cells, as well as eosinophils (P < 0.05) (Fig. 10E, F). Additionally, ssGSEA revealed distinct patterns of immune cell infiltration between the two groups and confirmed a significant correlation between the risk score and immune cell levels (Fig. 10G, H). These results highlight the critical role of tumor-infiltrating immune cells in differentiating between risk groups and suggest that they may serve as valuable targets for therapeutic intervention, providing new opportunities for improving patient prognosis.

### Analysis of immune checkpoints and tumor mutation load

**Fig. 11:**
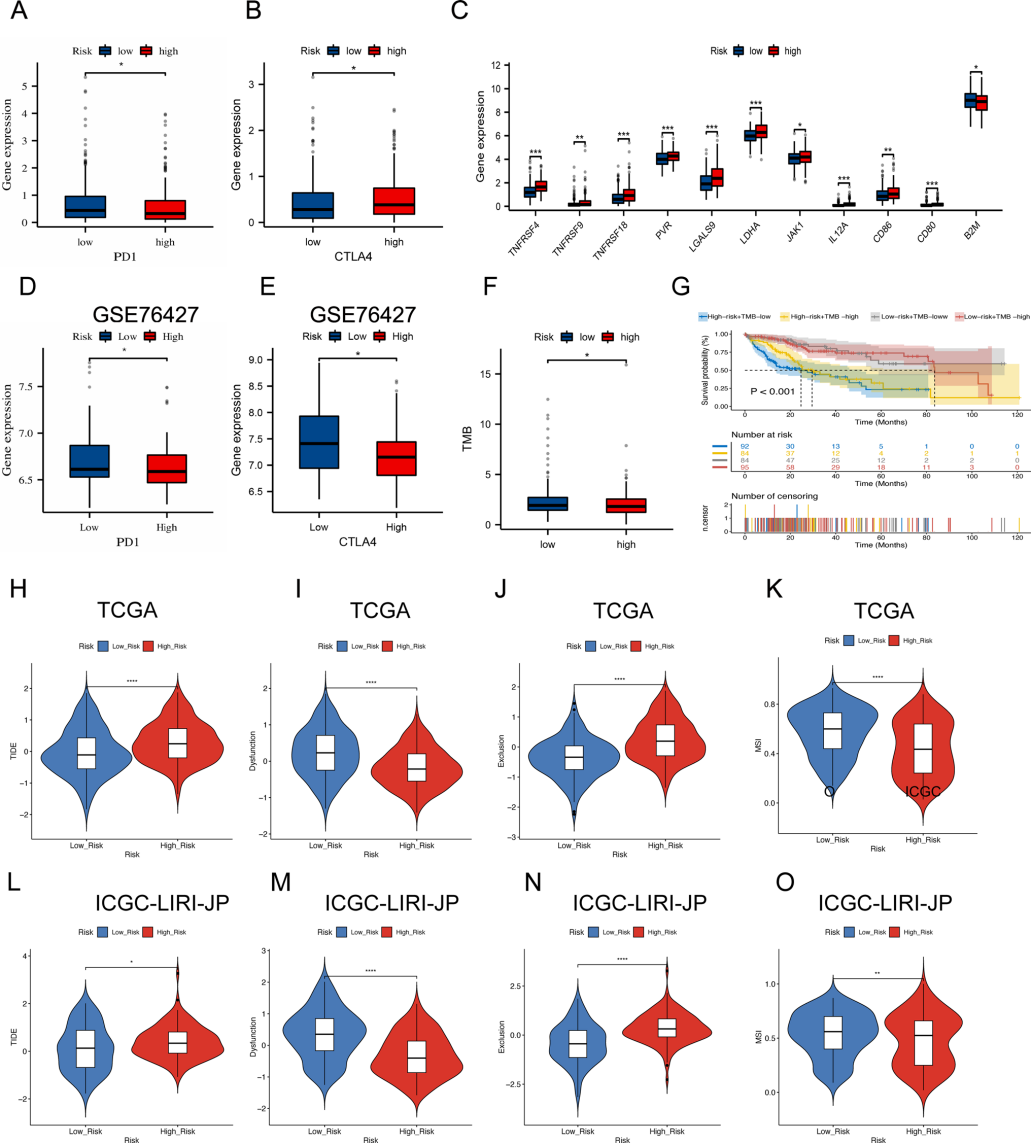
Analysis of immune checkpoints and tumor mutation load. **A** PD1 analysis; **B** CTLA4 analysis; **C** Analysis of other immune checkpoints; **D** PD1 analysis in GSE76427; **E** CTLA4 analysis in GSE76427; **F** Comparative analysis of tumor mutation loads; **G** Prognostic analysis of tumor mutation load in high and low-risk groups; **H**–**K** Analysis of TIDE scores in high and low-risk groups; **L**–**O** ICGC dataset validation of TIDE scores

Further analyses were conducted to uncover differences in immune checkpoint expression between high-risk and low-risk groups, revealing distinct immunomodulatory functions (Fig. 11A–C). The findings indicated that PD1, CTLA4, and B2M were significantly overexpressed in the low-risk group (P < 0.05), while the high-risk group exhibited higher levels of CD80, CD86, LDHA, IL12A, JAK1, LGALS9, PVR, TNFRSF18, and TNFRSF9 (P < 0.05). These results were corroborated by subsequent studies on the impact of immune checkpoints on LIHC prognosis in the GSE76427 dataset (Fig. 11D, E). The elevated expression of PD1 and CTLA4 in the low-risk group suggests a potentially higher responsiveness to immune checkpoint inhibitors. Additionally, a significant difference in tumor mutational load between the two groups was observed (P < 0.05), with higher mutation loads correlating with lower overall survival (Fig. 11F, G). The high-risk group also demonstrated significantly higher TIDE and Exclusion scores, alongside lower Dysfunction and MSI scores, indicating a greater likelihood of immune escape, which may diminish the efficacy of ICI therapy (Fig. 11H–K). Similar trends were observed in the ICGC dataset (Fig. 11L–O). Overall, our study concludes that the low-risk group is less likely to experience immune escape and may have better responses to immunotherapy compared to the high-risk group.

### Detection of mRNA expression in 8 prognostic DEGs

**Fig. 12:**
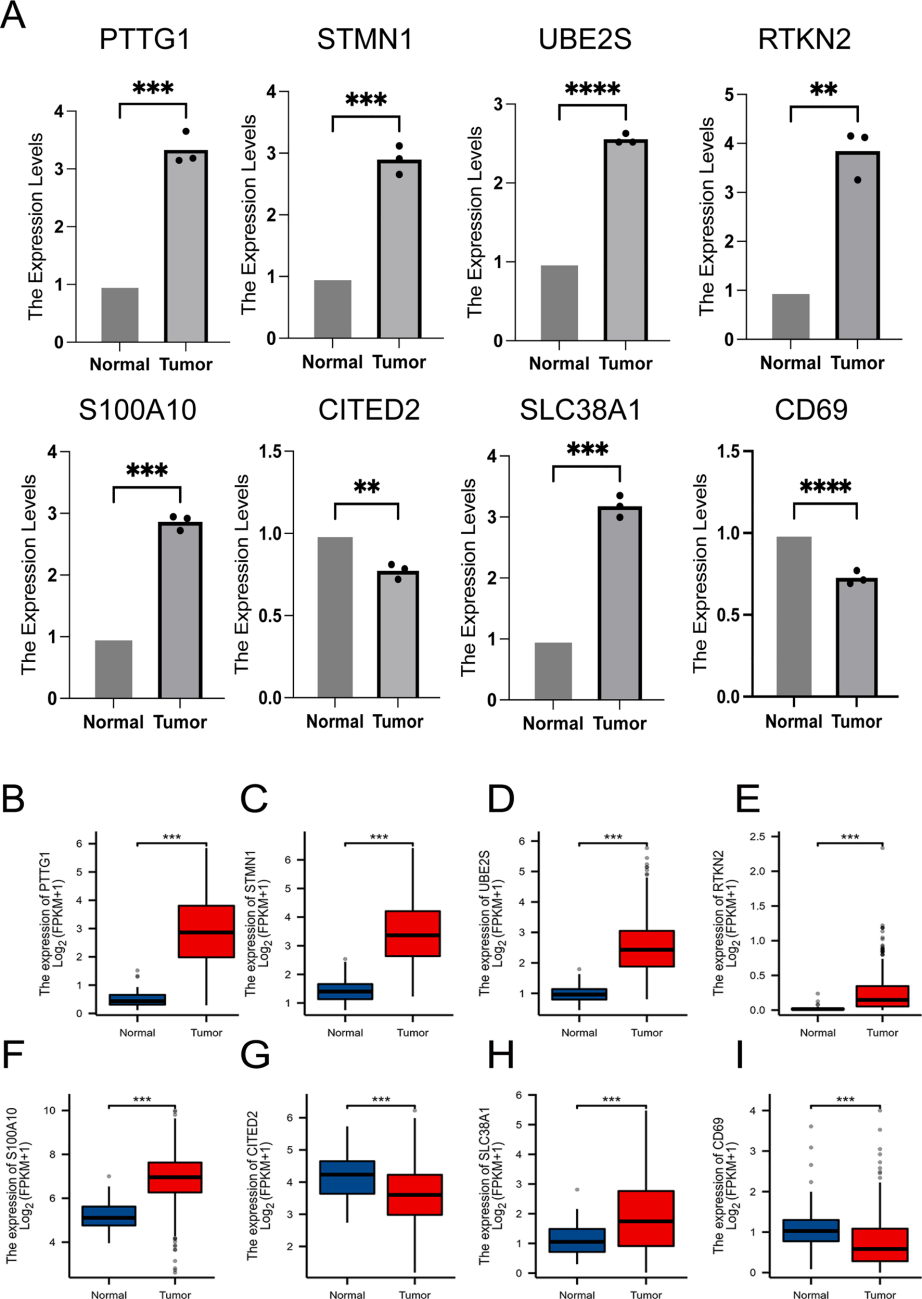
Differential expression analysis. **A** RT-qPCR validation of gene expression for constitutive risk models; **B**–**I** Differential expression analysis of genes in the TCGA database

To validate the mRNA levels of the eight prognostic differentially expressed genes (DEGs) in normal hepatocytes and hepatocellular carcinoma cells, real-time fluorescence quantitative PCR was performed. The results, presented in Fig. 12A–I, indicated that six prognostic DEGs exhibited higher expression in hepatocellular carcinoma cells than in normal hepatocytes, except for CITED2 and CD69.

## Discussion

Recent advances in single-cell sequencing have revolutionized our understanding of gene expression profiles at the individual cell level, providing groundbreaking insights for precision cancer therapy [20, 21]. High-throughput sequencing of both tumor and immune cells offers new perspectives for examining the tumor immune microenvironment, the occurrence and metastasis of hepatocellular carcinoma, and aspects including clinical diagnosis, individual therapeutic differences, prognosis, and assessment [22]. Hepatocellular carcinoma is marked by significant cellular heterogeneity within tumor tissues. Single-cell sequencing is instrumental in identifying biomarkers, particularly in understanding the role of T cells in the TME [22, 23], which could enhance the precision treatment of hepatocellular carcinoma. The scRNA-seq dataset used in this study, obtained from the GEO database, facilitated a unique analysis, distinct from previous research. This study focused on elucidating the specific roles of T-cell marker genes in the tumor microenvironment and leveraged their expression profiles to develop a prognostic risk model aimed at improving HCC patient outcomes. A T-cell prognostic risk model, based on the LIHC dataset from TCGA and centered on T-cell marker genes in hepatocellular carcinoma, was established. The model's reliability was validated using a dataset from the GEO database. Eight genes of prognostic importance were identified: PTTG1, STMN1, UBE2S, RTKN2, S100A10, CITED2, SLC38A1, and CD69.

Studies have demonstrated that high expression of PTTG1, the pituitary tumor transforming gene, is associated with increased T cell permeability in tumors [24, 25]. PTTG1 also plays a role in tumorigenesis by upregulating the transcription of asparagine synthetase, a crucial enzyme in polyamine biosynthesis, with T-cell infiltration closely linked to immune surveillance and the efficacy of immunotherapy [24]. T cells are essential in tumor immune responses, and PTTG1 may serve as a predictive biomarker for immune checkpoint blockade (ICB) responses. STMN1, a cytoplasmic phosphoprotein involved in microtubule dynamics, has been linked to the prognosis of various malignancies [26, 27]. Rui et al. [26] found that STMN1 mediates interactions between hepatocellular carcinoma cells and hepatic stellate cells through the Hepatocyte Growth Factor/MET signaling pathway, influencing tumor behavior and prognosis. Ubiquitination plays a critical role in LIHC progression, affecting processes such as protein degradation and signal transduction. The ubiquitin-conjugating enzyme E2S (UBE2S), a member of the ubiquitin-binding enzyme family, is associated with tumor size, stage, and TNM classification. UBE2S promotes AKT phosphorylation and reduces PTEN protein levels by facilitating PTEN ubiquitination at Lys60 and Lys327, impacting immune responses [28, 29]. S100A10, part of the S100 protein family, is involved in converting fibrinogen to an active protease [30]. Its upregulation in various cancers suggests its potential as a biomarker for cancer prognosis [31, 32]. SLC38A1, a glutamine transporter, plays a significant role in tumor cell migration and transport [33]. Yun Liu et al. [33] reported that increased SLC38A1 expression correlates with hepatocellular carcinoma prognosis and immune infiltration, corroborating our findings.

Rhotekin 2 (RTKN2) is recognized as an oncogene in locally advanced gastric cancer (GC). Although RTKN2 overexpression is known to enhance GC cell proliferation and migration, its specific role in liver hepatocellular carcinoma (LIHC) remains unexplored, suggesting a potential, yet unidentified, function in LIHC development [34]. Additionally, Zhao HG et al. [34] reported that RTKN2 significantly influences the Wnt/β-catenin signaling pathway. Hypoxia, a common feature in various cancers, involves crucial roles in the induction, activation, and stabilization of hypoxia-inducible factor 1 (HIF-1) in cancer's biological functions. CITED2, widely expressed, binds competitively to the CH1 domain of CBP/p300, a transcriptional co-activator, counteracting HIF-1, which is essential for tumor cell adaptation to hypoxic environments [35]. CITED2 functions as both a positive and negative regulator [28]. Molecular studies reveal that CITED2 deficiency results in increased IFN-γ-induced STAT1 transcriptional activity and enhanced STAT1 presence in macrophages. Atif Zafar et al. [36] demonstrated that CITED2 inhibits the STAT1-IRF1 signaling pathway in macrophages, reducing plaque formation in atherosclerosis. The roles of CITED2 and RTKN2 in predicting LIHC outcomes are yet to be thoroughly investigated. Given their established roles in other tumors, these genes could serve as novel biomarkers for LIHC prognosis, meriting further study.

In this study, we successfully constructed a prognostic risk model for HCC based on the TCGA-LIHC dataset and validated its robustness and predictive performance using multiple independent datasets, including GSE76427 and ICGC. One of the most significant findings is that the low-risk group exhibited significantly better overall survival (OS) than the high-risk group. The eight independent prognostic DEGs identified through multivariate Cox regression showed excellent predictive power in the TCGA cohort, with AUCs of 0.8, 0.76, and 0.76 for 1-year, 3-year, and 5-year OS prediction, respectively. These results were further validated in the GSE76427 and ICGC datasets, confirming the model's robustness and generalizability.

Compared to other hepatocellular carcinoma (HCC) prognostic models, our model offers notable advantages. For instance, the gene expression signature model developed by Hoshida et al. [37], while proficient in predicting HCC prognosis, yielded a moderate area under the curve (AUC) of approximately 0.74 in external validation datasets, indicating limited predictive accuracy. Conversely, our model achieved higher AUC values for overall survival (OS) at 1-year, 3-year, and 5-year intervals, with AUCs of 0.8, 0.76, and 0.76, respectively, suggesting an enhanced predictive capability for HCC prognosis. Furthermore, the immune-related gene signature model by Zhang et al. [38] reported AUCs of 0.73, 0.72, and 0.71 for predicting 1-year, 3-year, and 5-year OS in the TCGA validation cohort, highlighting the potential of immune genes in HCC prognosis. Zhou et al.'s [39] study, "T Cell-based Prognostic Prediction of Liver Cancer Patients," introduced a prognostic model focused on T cell depletion, achieving AUCs of 0.82, 0.75, and 0.72 for 1-year, 3-year, and 5-year OS, respectively. Despite the moderate predictive accuracy of their model, its limited validation in independent datasets suggests reduced generalizability.

Overall, our model not only demonstrates high predictive accuracy in the TCGA cohort but also maintains its robustness and generalizability across multiple independent datasets. Compared with other models, our model shows superior performance in long-term survival prediction, providing a new tool and direction for early diagnosis and personalized treatment of HCC patients.

These findings carry profound clinical implications. By pinpointing gene features intimately linked to patient prognosis, we have equipped clinicians with more precise tools for decision-making, thereby facilitating the identification of high-risk patients and the formulation of tailored treatment strategies. Specifically, the elevated expression of genes such as PTTG1, STMN1, and UBE2S is strongly correlated with unfavorable prognosis, whereas the expression of genes like CITED2 and SLC38A1 in the low-risk group is associated with more favorable outcomes. These genes are intricately involved in a myriad of biological processes, including cell cycle regulation, protein degradation, and metabolic regulation, underscoring their potential roles as pivotal drivers in the pathogenesis and progression of hepatocellular carcinoma.

Exhausted T cells are generally more prevalent in the tumor microenvironment due to persistent antigen stimulation and the presence of an immunosuppressive milieu. However, we observed a higher proportion of exhausted T cells in normal tissue compared to tumor tissue, indicating an underlying mechanism of immune balance. In normal tissues, chronic exposure to antigens, such as those from microbial or environmental sources, can lead to gradual T cell exhaustion, serving to maintain immune homeostasis and prevent excessive immune responses. Research has demonstrated that chronic antigen exposure in non-tumor environments can induce T cell exhaustion as a regulatory mechanism [40, 41].

Conversely, within the tumor microenvironment, tumor cells can secrete immunosuppressive molecules and modulate antigen presentation, thereby inhibiting the accumulation of exhausted T cells while maintaining a certain proportion of effector T cells. This may be a strategy that tumor cells use to evade immune surveillance, enabling them to escape immune responses while still manipulating the immune landscape to their advantage. This observation is consistent with existing literature, which suggests that tumors can create an immunosuppressive environment to selectively alter T cell function [42]. Therefore, the increased presence of exhausted T cells in normal tissue compared to tumor tissue highlights a potential shift in immune regulation mechanisms between these environments. This dynamic balance of immune regulation may play a crucial role in the tumor immune response.

In the complex interplay within the TME, comprising tumor cells, immune cells, stromal cells, and other elements [43], patients were classified into high- and low-risk groups based on eight prognostic gene analyses. The low-risk group, characterized by lower tumor purity, suggested a higher presence of non-tumor entities, such as immune and stromal cells, indicating a potentially stronger immune response compared to the high-risk group. The prognostic risk model independently predicted overall survival (OS) in LIHC patients, as confirmed by subsequent analyses. This prompted an exploration of the underlying immunological mechanisms. Immune infiltration analysis showed higher expression of memory B cells, follicular helper T cells, M0 macrophages, regulatory T cells, and neutrophils in the low-risk group (P < 0.05). In contrast, the high-risk group exhibited increased expression of CD8 + T cells, resting CD4 memory T cells, M1 macrophages, both resting and activated mast cells, and eosinophils (P < 0.05). The low-risk group had higher expression of B2M, PD1, and CTLA4 (P < 0.05), whereas the high-risk group showed elevated levels of CD80, CD86, LDHA, IL12A, JAK1, LGALS9, PVR, TNFRSF18, TNFRSF9, and LGALS9 (P < 0.05). Pathway enrichment analysis indicated a dominance of ribosomal transporter metabolism in the high-risk group and increased genomic transcriptional regulation in the low-risk group. These findings elucidate the distinct tumor biological behaviors of the risk groups. Immune checkpoint analysis revealed augmented expression of PD1, CTLA4, and B2M in the low-risk group (P < 0.05), suggesting a more favorable response to immune checkpoint inhibitors. Additionally, low-risk group patients with a high tumor mutation burden (TMB) demonstrated improved OS compared to those with low TMB. Cytological experiments on hepatocellular carcinoma cell lines validated the expression of relevant prognostic genes, consistent with TCGA dataset findings. In summary, our study establishes that the constructed risk model independently predicts LIHC prognosis and offers novel biomarkers for clinical assessment and treatment planning.

This study has several limitations, primarily, it relies on data from public databases, despite utilizing multiple datasets for validation. Nevertheless, to thoroughly validate our risk model and gain a deeper understanding of the molecular mechanisms of T-cell marker genes in LIHC, it is essential to conduct corroborating in vivo and in vitro experiments. In future research, we will concentrate on elucidating the underlying molecular mechanisms of these independent prognostic factors.

## Conclusion

In conclusion, our study constructed a single-cell atlas of LIHC using scRNA-seq datasets. Through enrichment analysis, immunoassays, and investigations into cell differentiation and communication, we revealed the heterogeneity of T cells in LIHC. Additionally, by integrating RNA-seq datasets, we advanced the identification of prognostic biomarkers for LIHC and established a T-cell risk model with independent prognostic significance. Our study provides novel insights into the regulation of tumor-infiltrating immune cells in LIHC. To validate the clinical relevance of these findings, further experimental and clinical studies are warranted.

## Supplementary Information

Supplementary material 1.

Supplementary material 2.

Supplementary material 3.

Supplementary material 4.

Supplementary material 5.

Supplementary material 6.

Supplementary material 7.

## Data Availability

The direct links required to find each data set in the database are as follows: the GEO gene expression and clinical pathology data set: https://www.ncbi.nlm.nih.gov/geo/query/acc.cgi?acc = GSE202642；https://www.ncbi.nlm.nih.gov/geo/query/acc.cgi?acc = GSE76427; The TCGA gene expression and clinical pathology data set: https://xenabrowser.net/datapages/?cohort = GDC%20TCGA%20Liver%20Cancer%20(LIHC)&removeHub = http%3A%2F%2F127.0.0.1%3A722.The data set downloaded by this direct link is the original data set.

## Abbreviations

OS: Overall survival
RT-qPCR: Real-time quantitative PCR
TMB: Tumor mutation burden
LIHC: Hepatocellular carcinoma
TME: Tumor microenvironment
SCT: Single cell sequencing technology
scRNA-seq: Single-cell RNA sequencing
PCA: Principal component analysis
TCGA: The Cancer Genome Atlas
ICGC: International Cancer Genome Consortium
GEO: Gene expression omnibus
GSEA: Gene set enrichment analysis
DMEM: Dulbecco's modified eagle medium
FBS: Fetal bovine serum

## References

[1] Donne R, Lujambio A. The liver cancer immune microenvironment: therapeutic implications for hepatocellular carcinoma. Hepatology. 2023;77(5):1773–96. 35989535 10.1002/hep.32740 PMC9941399

[2] Propper DJ, Balkwill FR. Harnessing cytokines and chemokines for cancer therapy. Nat Rev Clin Oncol. 2022;19(4):237–53. 34997230 10.1038/s41571-021-00588-9

[3] Sun Y, Wu L, Zhong Y, et al. Single-cell landscape of the ecosystem in early-relapse hepatocellular carcinoma. Cell. 2021;184(2):404-421.e416. 33357445 10.1016/j.cell.2020.11.041

[4] Bonnal RJP, Rossetti G, Lugli E, et al. Clonally expanded eomes(+) Tr1-like cells in primary and metastatic tumors are associated with disease progression. Nat Immunol. 2021;22(6):735–45. 34017124 10.1038/s41590-021-00930-4

[5] Francisco LM, Sage PT, Sharpe AH. The Pd-1 pathway in tolerance and autoimmunity. Immunol Rev. 2010;236:219–42. 20636820 10.1111/j.1600-065X.2010.00923.x PMC2919275

[6] Patel AP, Tirosh I, Trombetta JJ, et al. Single-cell Rna-Seq highlights intratumoral heterogeneity in primary glioblastoma. Science. 2014;344(6190):1396–401. 24925914 10.1126/science.1254257 PMC4123637

[7] Gordon SR, Maute RL, Dulken BW, et al. Pd-1 expression by tumour-associated macrophages inhibits phagocytosis and tumour immunity. Nature. 2017;545(7655):495–9. 28514441 10.1038/nature22396 PMC5931375

[8] Han C, Chen J, Huang J, et al. Single-cell transcriptome analysis reveals the metabolic changes and the prognostic value of malignant hepatocyte subpopulations and predict new therapeutic agents for hepatocellular carcinoma. Front Oncol. 2023;13:1104262. 36860314 10.3389/fonc.2023.1104262 PMC9969971

[9] Jerby-Arnon L, Shah P, Cuoco MS, et al. A cancer cell program promotes t cell exclusion and resistance to checkpoint blockade. Cell. 2018;175(4):984-997.e924. 30388455 10.1016/j.cell.2018.09.006 PMC6410377

[10] Stuart T, Butler A, Hoffman P, et al. Comprehensive integration of single-cell data. Cell. 2019;177(7):1888-1902.e1821. 31178118 10.1016/j.cell.2019.05.031 PMC6687398

[11] Hu C, Li T, Xu Y, et al. Cellmarker 2.0: an updated database of manually curated cell markers in human/mouse and web tools based on Scrna-Seq data. Nucleic Acids Res. 2023;51(D1):D870-d876. 36300619 10.1093/nar/gkac947 PMC9825416

[12] Monaco G, Lee B, Xu W, et al. Rna-seq signatures normalized by mrna abundance allow absolute deconvolution of human immune cell types. Cell Rep. 2019;26(6):1627-1640.e1627. 30726743 10.1016/j.celrep.2019.01.041 PMC6367568

[13] Yu G, Wang LG, Han Y, et al. Clusterprofiler: an R package for comparing biological themes among gene clusters. OMICS. 2012;16(5):284–7. 22455463 10.1089/omi.2011.0118 PMC3339379

[14] Jin S, Guerrero-Juarez CF, Zhang L, et al. Inference and analysis of cell-cell communication using cellchat. Nat Commun. 2021;12(1):1088. 33597522 10.1038/s41467-021-21246-9 PMC7889871

[15] Qiu X, Hill A, Packer J, et al. Single-cell Mrna quantification and differential analysis with census. Nat Methods. 2017;14(3):309–15. 28114287 10.1038/nmeth.4150 PMC5330805

[16] Newman AM, Liu CL, Green MR, et al. Robust enumeration of cell subsets from tissue expression profiles. Nat Methods. 2015;12(5):453–7. 25822800 10.1038/nmeth.3337 PMC4739640

[17] Barbie DA, Tamayo P, Boehm JS, et al. Systematic RNA interference reveals that oncogenic Kras-driven cancers require Tbk1. Nature. 2009;462(7269):108–12. 19847166 10.1038/nature08460 PMC2783335

[18] Xing X, Song J. Identification of the different gene expression characteristics from liver cirrhosis to hepatocellular carcinoma using single-cell sequencing analyses. J Immunol Res. 2021;2021:6619302. 33532508 10.1155/2021/6619302 PMC7834792

[19] Zhang J, Liu X, Huang Z, et al. T Cell-related prognostic risk model and tumor immune environment modulation in lung adenocarcinoma based on single-cell and bulk RNA sequencing. Comput Biol Med. 2023;152:106460. 36565482 10.1016/j.compbiomed.2022.106460

[20] Wang R, Li J, Zhou X, et al. Single-cell genomic and transcriptomic landscapes of primary and metastatic colorectal cancer tumors. Genome Med. 2022;14(1):93. 35974387 10.1186/s13073-022-01093-z PMC9380328

[21] Huuhtanen J, Kasanen H, Peltola K, et al. Single-cell characterization of Anti-Lag-3 and Anti-Pd-1 combination treatment in patients with melanoma. J Clin Invest. 2023. 10.1172/JCI164809. 36719749 10.1172/JCI164809 PMC10014104

[22] Parry EM, Lemvigh CK, Deng S, et al. Znf683 marks a Cd8(+) T cell population associated with anti-tumor immunity following anti-Pd-1 therapy for richter syndrome. Cancer Cell. 2023;41(10):1803-1816.e1808. 37738974 10.1016/j.ccell.2023.08.013 PMC10618915

[23] Liu Y, Zhang Q, Xing B, et al. Immune phenotypic linkage between colorectal cancer and liver metastasis. Cancer Cell. 2022;40(4):424-437.e425. 35303421 10.1016/j.ccell.2022.02.013

[24] Tien S, Zhou H, Zhou Q, et al. Pttg1 alleviates acute alcoholic liver injury by inhibiting endoplasmic reticulum stress-induced hepatocyte pyroptosis. Liver Int. 2023;43(4):840–54. 36737842 10.1111/liv.15535

[25] Zhou Q, Li L, Sha F, et al. Pttg1 reprograms asparagine metabolism to promote hepatocellular carcinoma progression. Cancer Res. 2023;83(14):2372–86. 37159932 10.1158/0008-5472.CAN-22-3561

[26] Zhang R, Gao X, Zuo J, et al. Stmn1 Upregulation mediates hepatocellular carcinoma and hepatic stellate cell crosstalk to aggravate cancer by triggering the met pathway. Cancer Sci. 2020;111(2):406–17. 31785057 10.1111/cas.14262 PMC7004522

[27] Zhang ED, Li C, Fang Y, et al. Stmn1 as a novel prognostic biomarker in hcc correlating with immune infiltrates and methylation. World J Surg Oncol. 2022;20(1):301. 36127700 10.1186/s12957-022-02768-y PMC9487063

[28] Zhang RY, Liu ZK, Wei D, et al. Ube2s interacting with Trim28 in the nucleus accelerates cell cycle by ubiquitination of P27 to promote hepatocellular carcinoma development. Signal Transduct Target Ther. 2021;6(1):64. 33589597 10.1038/s41392-020-00432-z PMC7884418

[29] Gui L, Zhang S, Xu Y, et al. ube2s promotes cell chemoresistance through Pten-Akt signaling in hepatocellular carcinoma. Cell Death Discov. 2021;7(1):357. 34785642 10.1038/s41420-021-00750-3 PMC8595659

[30] Saiki Y, Horii A. Multiple functions of S100a10, an important cancer promoter. Pathol Int. 2019;69(11):629–36. 31612598 10.1111/pin.12861

[31] Wang X, Huang H, Sze KM, et al. S100a10 promotes Hcc development and progression via transfer in extracellular vesicles and regulating their protein cargos. Gut. 2023;72(7):1370–84. 36631249 10.1136/gutjnl-2022-327998 PMC10314046

[32] Tantyo NA, Karyadi AS, Rasman SZ, et al. The prognostic value of S100a10 expression in cancer. Oncol Lett. 2019;17(2):1417–24. 30675195 10.3892/ol.2018.9751 PMC6341771

[33] Liu Y, Yang Y, Jiang L, et al. High expression levels of Slc38a1 are correlated with poor prognosis and defective immune infiltration in hepatocellular carcinoma. J Oncol. 2021;2021:5680968. 34697542 10.1155/2021/5680968 PMC8541878

[34] Zhao HG, Yin JJ, Chen X, et al. Rtkn2 enhances radioresistance in gastric cancer through regulating the wnt/β-catenin signalling pathway. Folia Biol (Praha). 2022;68(1):33–9. 36201856 10.14712/fb2022068010033

[35] Fernandes MT, Calado SM, Mendes-Silva L, et al. Cited2 and the modulation of the hypoxic response in cancer. World J Clin Oncol. 2020;11(5):260–74. 32728529 10.5306/wjco.v11.i5.260 PMC7360518

[36] Zafar A, Pong Ng H, Diamond-Zaluski R, et al. Cited2 inhibits Stat1-Irf1 signaling and atherogenesis. Faseb j. 2021;35(9):e21833. 34365659 10.1096/fj.202100792R PMC8607356

[37] Hoshida Y, Villanueva A, Kobayashi M, et al. Gene expression in fixed tissues and outcome in hepatocellular carcinoma. N Engl J Med. 2008;359(19):1995–2004. 18923165 10.1056/NEJMoa0804525 PMC2963075

[38] Wang Z, Zhu J, Liu Y, et al. Development and validation of a novel immune-related prognostic model in hepatocellular carcinoma. J Transl Med. 2020;18(1):67. 32046766 10.1186/s12967-020-02255-6 PMC7011553

[39] Zhou Y, Wu W, Cai W, et al. Prognostic prediction using a gene signature developed based on exhausted T cells for liver cancer patients. Heliyon. 2024;10(6):e28156. 38533068 10.1016/j.heliyon.2024.e28156 PMC10963654

[40] Wherry EJ, Ha SJ, Kaech SM, et al. Molecular signature of CD8+ T cell exhaustion during chronic viral infection [published correction appears in Immunity. 2007 Nov; 27(5): 824]. Immunity. 2007; 27(4): 670–684. 10.1016/j.immuni.2007.09.006 17950003

[41] Mao Y, Poschke I, Kiessling R. Tumour-induced immune suppression: role of inflammatory mediators released by myelomonocytic cells. J Intern Med. 2014;276(2):154–70. 24597954 10.1111/joim.12229

[42] Pardoll DM. The blockade of immune checkpoints in cancer immunotherapy. Nat Rev Cancer. 2012;12(4):252–64. 22437870 10.1038/nrc3239 PMC4856023

[43] Christofides A, Strauss L, Yeo A, et al. The complex role of tumor-infiltrating macrophages. Nat Immunol. 2022;23(8):1148–56. 35879449 10.1038/s41590-022-01267-2 PMC10754321

