# Supplementary material for: Prognostic risk model of LIHC T-cells based on scRNA-seq and RNA-seq and the regulation of the tumor immune microenvironment
Source: Discov Oncol. 2024 Oct 10;15:540. doi: 10.1007/s12672-024-01424-z (PMC11467143; doi:10.1007/s12672-024-01424-z)
Supplement: Supplementary file 1 — Supplementary material 1. [file 12672_2024_1424_MOESM1_ESM.pdf]

A

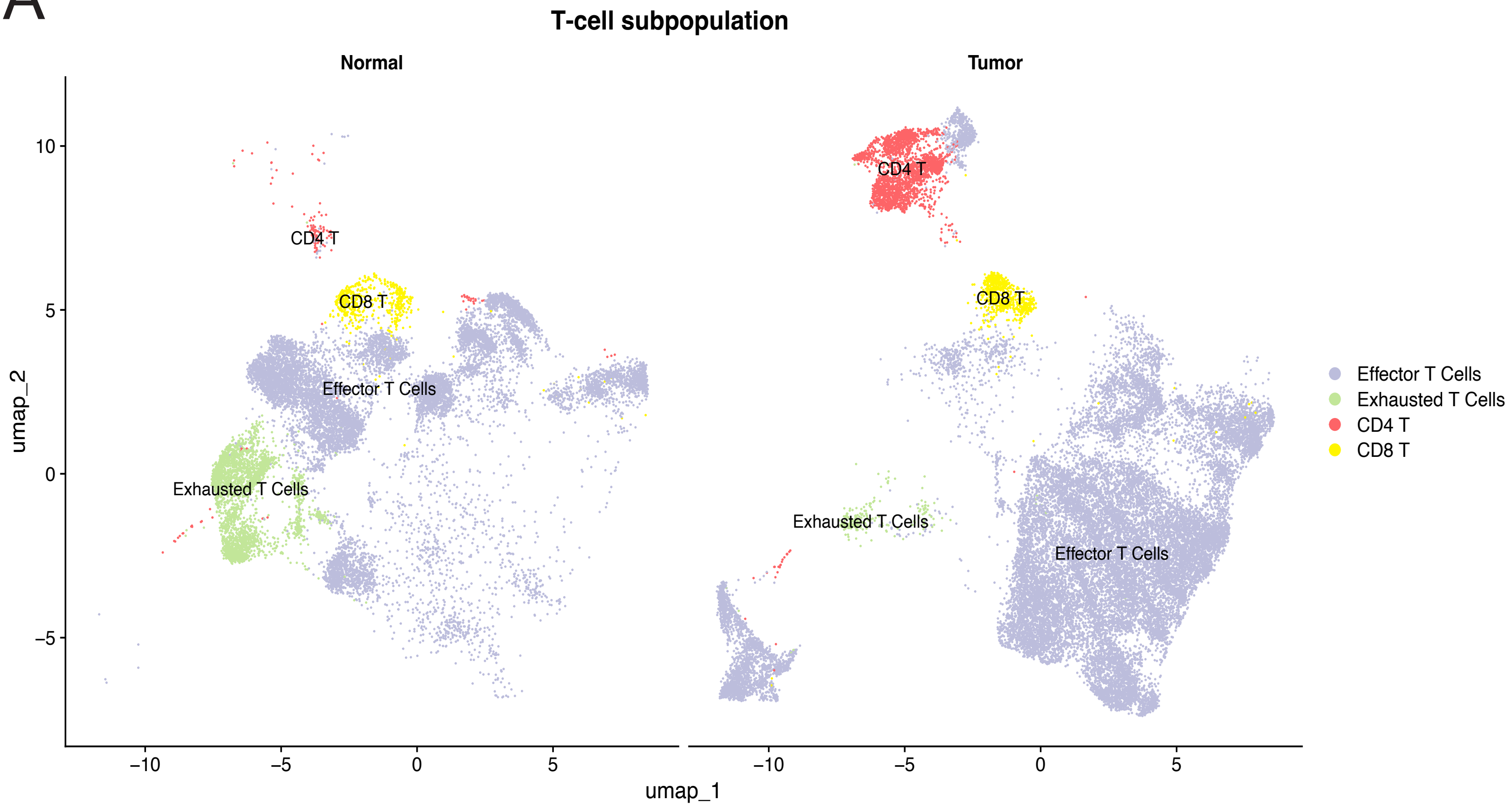

B

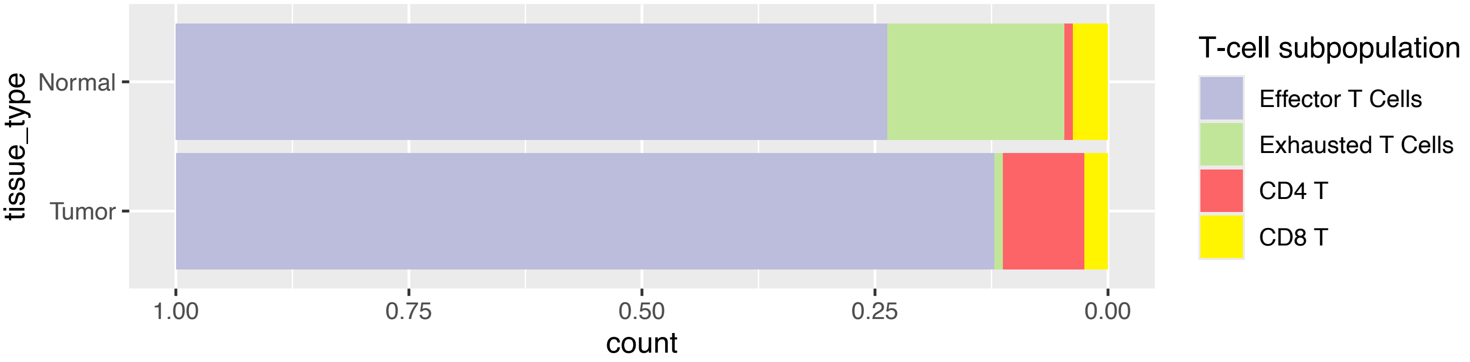

SF1. Construction of a Single-Cell Atlas. (A) UMAP plot representing T cell subpopulations; (B) cell scale plot depicting relative proportions;
