# Supplementary material for: Prognostic risk model of LIHC T-cells based on scRNA-seq and RNA-seq and the regulation of the tumor immune microenvironment
Source: Discov Oncol. 2024 Oct 10;15:540. doi: 10.1007/s12672-024-01424-z (PMC11467143; doi:10.1007/s12672-024-01424-z)
Supplement: Supplementary file 2 — Supplementary material 2. [file 12672_2024_1424_MOESM2_ESM.doc]

**s**
