# Supplementary material for: Prognostic risk model of LIHC T-cells based on scRNA-seq and RNA-seq and the regulation of the tumor immune microenvironment
Source: Discov Oncol. 2024 Oct 10;15:540. doi: 10.1007/s12672-024-01424-z (PMC11467143; doi:10.1007/s12672-024-01424-z)
Supplement: Supplementary file 3 — Supplementary material 3. [file 12672_2024_1424_MOESM3_ESM.doc]

| **Supplementary Table 2 KEGG enrichment analysis results** | | | | | | | | |
| --- | --- | --- | --- | --- | --- | --- | --- | --- |
| ID | Description | GeneRatio | BgRatio | pvalue | p.adjust | qvalue | geneID | Count |
| hsa04650 | Natural killer cell mediated cytotoxicity | 18/114 | 132/8586 | 8.21E-14 | 1.70E-11 | 1.32E-11 | 3458/3002/3824/22914/5551/5295/2534/919/3107/962/10870/3133/3932/3105/3106/5880/3683/4068 | 18 |
| hsa04612 | Antigen processing and presentation | 14/114 | 78/8586 | 1.18E-12 | 1.22E-10 | 9.51E-11 | 3458/3824/3320/925/3312/3304/3326/3107/3303/3134/3133/3105/3106/3310 | 14 |
| hsa05332 | Graft-versus-host disease | 9/114 | 42/8586 | 2.88E-09 | 1.99E-07 | 1.55E-07 | 3458/3002/3824/5551/3107/3134/3133/3105/3106 | 9 |
| hsa05416 | Viral myocarditis | 10/114 | 60/8586 | 4.94E-09 | 2.56E-07 | 1.99E-07 | 5551/2534/3107/3134/3133/54205/3105/3106/5880/3683 | 10 |
| hsa05170 | Human immunodeficiency virus 1 infection | 16/114 | 212/8586 | 1.65E-08 | 6.84E-07 | 5.32E-07 | 3725/5295/801/915/916/917/919/3107/3134/3133/7852/54205/3105/3106/5880/54331 | 16 |
| hsa05330 | Allograft rejection | 8/114 | 38/8586 | 2.65E-08 | 9.13E-07 | 7.11E-07 | 3458/3002/5551/3107/3134/3133/3105/3106 | 8 |
| hsa04660 | T cell receptor signaling pathway | 12/114 | 121/8586 | 5.82E-08 | 1.72E-06 | 1.34E-06 | 3458/5527/925/3725/5295/915/2534/916/917/919/5788/3932 | 12 |
| hsa04940 | Type I diabetes mellitus | 8/114 | 43/8586 | 7.43E-08 | 1.92E-06 | 1.50E-06 | 3458/3002/5551/3107/3134/3133/3105/3106 | 8 |
| hsa05163 | Human cytomegalovirus infection | 15/114 | 225/8586 | 2.47E-07 | 5.69E-06 | 4.43E-06 | 6352/6351/9560/5295/801/3107/3134/3133/7852/54205/3105/3106/5880/54331/11214 | 15 |
| hsa05169 | Epstein-Barr virus infection | 14/114 | 202/8586 | 4.01E-07 | 8.29E-06 | 6.45E-06 | 864/3725/5295/915/916/917/919/3107/3134/3133/54205/3105/3106/3683 | 14 |
| hsa04659 | Th17 cell differentiation | 10/114 | 108/8586 | 1.49E-06 | 2.71E-05 | 2.11E-05 | 3458/3320/3725/915/916/917/919/3326/6095/3932 | 10 |
| hsa05167 | Kaposi sarcoma-associated herpesvirus infection | 13/114 | 194/8586 | 1.57E-06 | 2.71E-05 | 2.11E-05 | 3725/5295/801/7314/3107/3134/9976/3133/54205/3105/3106/54331/7316 | 13 |
| hsa05320 | Autoimmune thyroid disease | 7/114 | 53/8586 | 5.63E-06 | 8.96E-05 | 6.97E-05 | 3002/5551/3107/3134/3133/3105/3106 | 7 |
| hsa05166 | Human T-cell leukemia virus 1 infection | 13/114 | 222/8586 | 7.00E-06 | 0.000103565 | 8.06E-05 | 3725/5295/915/916/917/2113/3107/3134/3133/3932/3105/3106/3683 | 13 |
| hsa05162 | Measles | 10/114 | 138/8586 | 1.36E-05 | 0.00018813 | 0.000146371 | 3312/3725/5295/915/916/3304/917/3303/54205/3310 | 10 |
| hsa05235 | PD-L1 expression and PD-1 checkpoint pathway in cancer | 8/114 | 89/8586 | 2.18E-05 | 0.000281953 | 0.000219368 | 3458/3725/5295/915/916/917/919/3932 | 8 |
| hsa04658 | Th1 and Th2 cell differentiation | 8/114 | 92/8586 | 2.78E-05 | 0.000338387 | 0.000263276 | 3458/864/3725/915/916/917/919/3932 | 8 |
| hsa04514 | Cell adhesion molecules | 10/114 | 158/8586 | 4.41E-05 | 0.000507574 | 0.000394909 | 925/914/5788/3107/3134/3133/3105/3106/3683/4267 | 10 |
| hsa05142 | Chagas disease | 8/114 | 102/8586 | 5.87E-05 | 0.000639023 | 0.00049718 | 6352/3458/3725/5295/915/916/917/919 | 8 |
| hsa04380 | Osteoclast differentiation | 9/114 | 135/8586 | 7.22E-05 | 0.000747154 | 0.00058131 | 3458/3727/3726/3725/5295/2534/2354/3932/8651 | 9 |
| hsa04210 | Apoptosis | 9/114 | 136/8586 | 7.65E-05 | 0.00075363 | 0.000586348 | 3002/7277/5551/1521/3725/5295/7846/54205/5366 | 9 |
| hsa04915 | Estrogen signaling pathway | 9/114 | 137/8586 | 8.09E-05 | 0.000761496 | 0.000592469 | 3320/3312/3725/5295/801/3304/3326/3303/3310 | 9 |
| hsa05417 | Lipid and atherosclerosis | 11/114 | 215/8586 | 0.0001261 | 0.001091868 | 0.000849509 | 6352/3320/3312/3725/5295/801/3304/3326/3303/54205/3310 | 11 |
| hsa03040 | Spliceosome | 11/114 | 216/8586 | 0.0001314 | 0.001091868 | 0.000849509 | 6432/3312/2521/3304/3303/1655/6430/6427/9939/6428/3310 | 11 |
| hsa05340 | Primary immunodeficiency | 5/114 | 38/8586 | 0.0001337 | 0.001091868 | 0.000849509 | 925/915/916/5788/3932 | 5 |
| hsa04670 | Leukocyte transendothelial migration | 8/114 | 115/8586 | 0.0001371 | 0.001091868 | 0.000849509 | 5295/7430/399/7852/10627/5880/3683/4267 | 8 |
| hsa04145 | Phagosome | 9/114 | 152/8586 | 0.0001795 | 0.001376006 | 0.001070577 | 7277/7846/3107/10383/3134/3133/3105/3106/11151 | 9 |
| hsa04218 | Cellular senescence | 9/114 | 156/8586 | 0.0002183 | 0.001614055 | 0.001255786 | 5295/801/678/2113/3107/3134/3133/3105/3106 | 9 |
| hsa05020 | Prion disease | 12/114 | 272/8586 | 0.0002489 | 0.001776734 | 0.001382356 | 6352/7277/3312/5295/2534/3304/7846/3303/10383/54205/5880/3310 | 12 |
| hsa04141 | Protein processing in endoplasmic reticulum | 9/114 | 170/8586 | 0.0004135 | 0.002853262 | 0.002219929 | 3337/3320/3301/3312/3304/3326/3303/10808/3310 | 9 |
| hsa05132 | Salmonella infection | 11/114 | 249/8586 | 0.0004506 | 0.003008566 | 0.002340761 | 7277/3320/3725/6188/3326/5788/7846/10383/399/54205/10627 | 11 |
| hsa04144 | Endocytosis | 11/114 | 250/8586 | 0.0004661 | 0.003015044 | 0.002345801 | 3312/3304/3107/3303/9744/3134/3133/7852/3105/3106/3310 | 11 |
| hsa05418 | Fluid shear stress and atherosclerosis | 8/114 | 139/8586 | 0.0005016 | 0.003146284 | 0.00244791 | 3458/3320/3725/10365/5295/801/3326/5880 | 8 |
| hsa05145 | Toxoplasmosis | 7/114 | 111/8586 | 0.000659 | 0.004012298 | 0.003121696 | 3458/3312/3304/3303/54205/8651/3310 | 7 |
| hsa05134 | Legionellosis | 5/114 | 56/8586 | 0.0008416 | 0.004977444 | 0.003872611 | 3312/3304/3303/54205/3310 | 5 |
| hsa05130 | Pathogenic Escherichia coli infection | 9/114 | 198/8586 | 0.0012355 | 0.006957553 | 0.005413199 | 7277/3725/2534/6188/7846/10383/7430/54205/4691 | 9 |
| hsa04213 | Longevity regulating pathway - multiple species | 5/114 | 61/8586 | 0.0012436 | 0.006957553 | 0.005413199 | 3312/5295/3304/3303/3310 | 5 |
| hsa04657 | IL-17 signaling pathway | 6/114 | 94/8586 | 0.0015139 | 0.008246768 | 0.00641625 | 3458/3727/3320/3725/2354/3326 | 6 |
| hsa04640 | Hematopoietic cell lineage | 6/114 | 99/8586 | 0.0019749 | 0.010482255 | 0.00815553 | 925/915/916/917/914/924 | 6 |
| hsa04062 | Chemokine signaling pathway | 8/114 | 192/8586 | 0.0039427 | 0.020403485 | 0.015874565 | 6352/6351/6846/9560/5295/7852/5880/54331 | 8 |
| hsa05210 | Colorectal cancer | 5/114 | 86/8586 | 0.0056034 | 0.027935083 | 0.021734389 | 3725/5295/54205/5366/5880 | 5 |
| hsa05203 | Viral carcinogenesis | 8/114 | 204/8586 | 0.005668 | 0.027935083 | 0.021734389 | 3725/5295/3107/3134/3133/3105/3106/5366 | 8 |
| hsa04010 | MAPK signaling pathway | 10/114 | 301/8586 | 0.0065712 | 0.031633355 | 0.024611763 | 1844/3727/3312/3725/3304/3303/6789/5880/1847/3310 | 10 |
| hsa04061 | Viral protein interaction with cytokine and cytokine receptor | 5/114 | 100/8586 | 0.0104699 | 0.049256033 | 0.038322771 | 6352/6351/6846/9560/7852 | 5 |
| hsa03010 | Ribosome | 77/143 | 167/8586 | 4.34E-100 | 7.73E-98 | 6.72E-98 | 6188/6122/6206/6204/6235/6156/6134/6232/6164/6191/6233/6230/6189/6194/6137/3921/6222/6208/6161/6187/6171/6227/4736/6125/6147/6210/6165/6139/6144/6143/6181/6207/6176/23521/6141/6133/6158/6135/6202/6167/6142/6193/6224/25873/6228/6160/6217/6223/6234/6173/6170/6132/6175/6169/6130/6157/6154/6152/6201/6209/6168/2197/6155/6203/6146/6159/6128/6124/6218/6138/6136/6129/9045/6166/11224/6192/7311 | 77 |
| hsa05171 | Coronavirus disease - COVID-19 | 80/143 | 232/8586 | 8.00E-92 | 7.12E-90 | 6.19E-90 | 6188/6122/6206/6204/6235/6156/6134/6232/6164/6191/6233/6230/6189/6194/6137/3921/6222/6208/6161/6187/6171/6227/4736/6125/6147/6210/6165/6139/6144/6143/4792/6181/6207/6176/23521/6141/6133/6158/6135/6202/6167/6142/6193/6224/25873/6228/6160/6217/6223/6234/6173/6170/6132/6175/6169/6130/6157/6154/6152/6201/3725/6209/6168/2197/6155/6203/6146/6159/6128/6124/7124/6218/6138/6136/6129/9045/6166/11224/6192/7311 | 80 |
| hsa04657 | IL-17 signaling pathway | 9/143 | 94/8586 | 2.45E-05 | 0.001454165 | 0.001264117 | 6364/7128/3727/4792/2354/3326/3725/7124/3320 | 9 |
| hsa04659 | Th17 cell differentiation | 8/143 | 108/8586 | 0.0004214 | 0.018751775 | 0.01630107 | 4792/6095/916/915/3326/3725/917/3320 | 8 |
| hsa04660 | T cell receptor signaling pathway | 8/143 | 121/8586 | 0.0008986 | 0.031988653 | 0.027807995 | 4792/916/915/3725/7124/5527/917/959 | 8 |
| hsa04137 | Mitophagy - animal | 6/143 | 72/8586 | 0.0012171 | 0.032414111 | 0.028177849 | 6233/3725/54543/7314/10370/7311 | 6 |
| hsa04640 | Hematopoietic cell lineage | 7/143 | 99/8586 | 0.0012747 | 0.032414111 | 0.028177849 | 3575/916/915/914/7124/960/917 | 7 |
| hsa05162 | Measles | 8/143 | 138/8586 | 0.0020917 | 0.045629187 | 0.039665822 | 7128/4792/3312/916/915/3725/10399/917 | 8 |
| hsa05134 | Legionellosis | 5/143 | 56/8586 | 0.0023071 | 0.045629187 | 0.039665822 | 1915/4792/3312/1937/7124 | 5 |
| hsa05150 | Staphylococcus aureus infection | 22/133 | 96/8586 | 2.62E-20 | 5.63E-18 | 4.66E-18 | 713/712/714/3123/3122/3113/3127/3115/3117/718/3119/3108/2266/2214/2212/3075/1675/3109/2359/717/629/719 | 22 |
| hsa04610 | Complement and coagulation cascades | 19/133 | 86/8586 | 2.51E-17 | 2.70E-15 | 2.23E-15 | 713/712/714/7448/1191/2/5265/2244/718/2243/3827/2266/3075/1675/717/629/710/719/11326 | 19 |
| hsa04612 | Antigen processing and presentation | 17/133 | 78/8586 | 1.70E-15 | 1.22E-13 | 1.01E-13 | 1508/5641/972/3123/3122/3113/3127/1514/3115/1520/3117/3119/3108/3109/821/10437/920 | 17 |
| hsa04142 | Lysosome | 20/133 | 132/8586 | 8.07E-15 | 4.34E-13 | 3.59E-13 | 1508/5641/1509/5660/3988/968/10577/967/1522/1514/1520/427/54/1200/57192/2517/950/1075/3920/5476 | 20 |
| hsa04145 | Phagosome | 21/133 | 152/8586 | 1.10E-14 | 4.72E-13 | 3.91E-13 | 3123/3122/3113/3127/4481/1514/929/3115/1520/4360/3117/718/3119/3108/2214/2212/1536/3109/821/60/3920 | 21 |
| hsa05152 | Tuberculosis | 20/133 | 180/8586 | 3.29E-12 | 1.18E-10 | 9.75E-11 | 1509/972/3123/3122/3113/3127/929/3115/1520/2207/4360/3117/718/3119/3108/2214/2212/3109/808/3920 | 20 |
| hsa04979 | Cholesterol metabolism | 12/133 | 51/8586 | 1.10E-11 | 3.37E-10 | 2.79E-10 | 348/336/341/344/335/3988/345/350/10577/338/4035/19 | 12 |
| hsa05310 | Asthma | 10/133 | 31/8586 | 1.90E-11 | 5.12E-10 | 4.23E-10 | 3123/3122/3113/3127/3115/2207/3117/3119/3108/3109 | 10 |
| hsa05140 | Leishmaniasis | 13/133 | 77/8586 | 1.29E-10 | 3.09E-09 | 2.56E-09 | 3123/3122/3113/3127/3115/3117/718/3119/3108/2214/2212/1536/3109 | 13 |
| hsa05322 | Systemic lupus erythematosus | 16/133 | 137/8586 | 2.68E-10 | 5.76E-09 | 4.77E-09 | 713/712/714/3123/3122/3113/3127/3115/3117/718/3119/3108/2214/2212/3109/717 | 16 |
| hsa05330 | Allograft rejection | 9/133 | 38/8586 | 4.36E-09 | 8.52E-08 | 7.05E-08 | 3123/3122/3113/3127/3115/3117/3119/3108/3109 | 9 |
| hsa05332 | Graft-versus-host disease | 9/133 | 42/8586 | 1.13E-08 | 2.03E-07 | 1.68E-07 | 3123/3122/3113/3127/3115/3117/3119/3108/3109 | 9 |
| hsa04940 | Type I diabetes mellitus | 9/133 | 43/8586 | 1.41E-08 | 2.34E-07 | 1.93E-07 | 3123/3122/3113/3127/3115/3117/3119/3108/3109 | 9 |
| hsa05416 | Viral myocarditis | 10/133 | 60/8586 | 2.21E-08 | 3.40E-07 | 2.81E-07 | 3123/3122/3113/3127/3115/3117/3119/3108/3109/60 | 10 |
| hsa04640 | Hematopoietic cell lineage | 12/133 | 99/8586 | 3.40E-08 | 4.88E-07 | 4.04E-07 | 3123/3122/3113/3127/929/3115/3117/3119/3108/1436/3109/920 | 12 |
| hsa04672 | Intestinal immune network for IgA production | 9/133 | 49/8586 | 4.76E-08 | 6.22E-07 | 5.15E-07 | 3123/3122/3113/3127/3115/3117/3119/3108/3109 | 9 |
| hsa05321 | Inflammatory bowel disease | 10/133 | 65/8586 | 4.92E-08 | 6.22E-07 | 5.15E-07 | 3123/3122/3113/3127/3115/3117/3119/3108/4094/3109 | 10 |
| hsa05320 | Autoimmune thyroid disease | 9/133 | 53/8586 | 9.74E-08 | 1.16E-06 | 9.63E-07 | 3123/3122/3113/3127/3115/3117/3119/3108/3109 | 9 |
| hsa04658 | Th1 and Th2 cell differentiation | 11/133 | 92/8586 | 1.50E-07 | 1.70E-06 | 1.41E-06 | 3123/3122/3113/3127/3115/3117/3119/3108/4094/3109/920 | 11 |
| hsa05323 | Rheumatoid arthritis | 11/133 | 93/8586 | 1.68E-07 | 1.81E-06 | 1.50E-06 | 3123/3122/3113/3127/1514/3115/3117/3119/3108/3109/54 | 11 |
| hsa04216 | Ferroptosis | 7/133 | 41/8586 | 2.65E-06 | 2.71E-05 | 2.24E-05 | 30061/2512/7018/3162/1356/1536/8031 | 7 |
| hsa04659 | Th17 cell differentiation | 10/133 | 108/8586 | 6.05E-06 | 5.92E-05 | 4.90E-05 | 3123/3122/3113/3127/3115/3117/3119/3108/3109/920 | 10 |
| hsa05171 | Coronavirus disease - COVID-19 | 14/133 | 232/8586 | 1.32E-05 | 0.000123204 | 0.000101941 | 713/712/714/2244/718/2243/2266/8829/2212/1536/1675/717/629/719 | 14 |
| hsa05133 | Pertussis | 8/133 | 76/8586 | 2.09E-05 | 0.000187173 | 0.00015487 | 713/712/714/929/718/808/717/710 | 8 |
| hsa04514 | Cell adhesion molecules | 11/133 | 158/8586 | 3.16E-05 | 0.000271944 | 0.000225011 | 3123/3122/3113/3127/3115/3117/7412/3119/3108/3109/920 | 11 |
| hsa04978 | Mineral absorption | 7/133 | 60/8586 | 3.56E-05 | 0.000294588 | 0.000243748 | 30061/2512/7018/3162/4502/481/475 | 7 |
| hsa05145 | Toxoplasmosis | 9/133 | 111/8586 | 5.21E-05 | 0.000414787 | 0.000343202 | 3123/3122/3113/3127/3115/3117/3119/3108/3109 | 9 |
| hsa03320 | PPAR signaling pathway | 7/133 | 75/8586 | 0.000151 | 0.001159545 | 0.000959428 | 336/335/345/2171/2168/6319/1622 | 7 |
| hsa05166 | Human T-cell leukemia virus 1 infection | 12/133 | 222/8586 | 0.0001616 | 0.001198174 | 0.00099139 | 3123/3122/3113/3127/3115/3117/3119/3108/8829/3109/821/920 | 12 |
| hsa05418 | Fluid shear stress and atherosclerosis | 9/133 | 139/8586 | 0.0002933 | 0.002102134 | 0.001739343 | 1514/7412/3162/9446/7295/808/7184/60/4257 | 9 |
| hsa05164 | Influenza A | 10/133 | 171/8586 | 0.0003088 | 0.002141786 | 0.001772151 | 3123/3122/3113/3127/3115/3117/3119/3108/3109/60 | 10 |
| hsa04936 | Alcoholic liver disease | 9/133 | 142/8586 | 0.0003438 | 0.002310045 | 0.001911371 | 713/712/714/2168/929/6319/718/717/719 | 9 |
| hsa04918 | Thyroid hormone synthesis | 6/133 | 75/8586 | 0.0010356 | 0.006747321 | 0.00558285 | 213/7276/2878/481/821/7184 | 6 |
| hsa04613 | Neutrophil extracellular trap formation | 9/133 | 191/8586 | 0.0028059 | 0.017743346 | 0.014681153 | 2244/718/2243/2266/2214/2212/1536/2359/60 | 9 |
| hsa05169 | Epstein-Barr virus infection | 9/133 | 202/8586 | 0.004071 | 0.025007366 | 0.020691529 | 3123/3122/3113/3127/3115/3117/3119/3108/3109 | 9 |
| hsa04975 | Fat digestion and absorption | 4/133 | 43/8586 | 0.004255 | 0.025412032 | 0.021026357 | 335/338/2168/19 | 4 |
| hsa04380 | Osteoclast differentiation | 7/133 | 135/8586 | 0.0048967 | 0.028453712 | 0.023543096 | 7305/2214/2212/1436/10990/54/54209 | 7 |
| hsa04210 | Apoptosis | 7/133 | 136/8586 | 0.0050973 | 0.028839809 | 0.02386256 | 1508/1509/1522/1514/1520/60/1075 | 7 |
| hsa05152 | Tuberculosis | 23/138 | 180/8586 | 6.94E-15 | 1.53E-12 | 1.15E-12 | 3553/3115/3122/3117/3113/3119/972/3123/3118/1520/3108/1051/2207/2212/8767/2213/3687/64581/3109/7879/4046/637/3329 | 23 |
| hsa05323 | Rheumatoid arthritis | 17/138 | 93/8586 | 7.19E-14 | 7.91E-12 | 5.98E-12 | 3553/3115/3122/3117/3113/3119/3123/3118/2920/7422/3108/942/3576/3109/10673/6349/6348 | 17 |
| hsa05310 | Asthma | 11/138 | 31/8586 | 7.96E-13 | 5.84E-11 | 4.41E-11 | 3115/3122/3117/3113/3119/3123/3118/2205/3108/2207/3109 | 11 |
| hsa04940 | Type I diabetes mellitus | 12/138 | 43/8586 | 1.84E-12 | 1.01E-10 | 7.65E-11 | 3553/3115/3122/3117/3113/3119/3123/3118/3108/942/3109/3329 | 12 |
| hsa05416 | Viral myocarditis | 13/138 | 60/8586 | 7.32E-12 | 3.22E-10 | 2.44E-10 | 3115/3122/3117/3113/3119/3123/3118/3108/60/1604/942/3109/637 | 13 |
| hsa04612 | Antigen processing and presentation | 14/138 | 78/8586 | 1.68E-11 | 6.16E-10 | 4.66E-10 | 3115/3122/10437/3117/3113/3119/972/3123/3118/1520/3108/3109/3303/3310 | 14 |
| hsa05332 | Graft-versus-host disease | 11/138 | 42/8586 | 3.46E-11 | 1.07E-09 | 8.12E-10 | 3553/3115/3122/3117/3113/3119/3123/3118/3108/942/3109 | 11 |
| hsa04640 | Hematopoietic cell lineage | 15/138 | 99/8586 | 3.91E-11 | 1.07E-09 | 8.12E-10 | 3553/3115/3122/3117/3113/3119/3123/3118/911/3108/1604/913/3109/1438/960 | 15 |
| hsa05171 | Coronavirus disease - COVID-19 | 21/138 | 232/8586 | 9.79E-11 | 2.39E-09 | 1.81E-09 | 3553/6205/6229/9349/114548/1839/2212/6203/728/6202/6207/6154/3576/6228/6129/1536/7311/6218/6189/6173/6142 | 21 |
| hsa05140 | Leishmaniasis | 13/138 | 77/8586 | 2.06E-10 | 4.28E-09 | 3.24E-09 | 3553/3115/3122/3117/3113/3119/3123/3118/3108/2212/3109/1536/5743 | 13 |
| hsa04672 | Intestinal immune network for IgA production | 11/138 | 49/8586 | 2.14E-10 | 4.28E-09 | 3.24E-09 | 3115/3122/3117/3113/3119/3123/3118/3108/942/3109/10673 | 11 |
| hsa04145 | Phagosome | 17/138 | 152/8586 | 2.57E-10 | 4.52E-09 | 3.42E-09 | 3115/3122/3117/3113/3119/3123/3118/4973/1520/3108/60/2212/2213/64581/3109/7879/1536 | 17 |
| hsa05330 | Allograft rejection | 10/138 | 38/8586 | 2.67E-10 | 4.52E-09 | 3.42E-09 | 3115/3122/3117/3113/3119/3123/3118/3108/942/3109 | 10 |
| hsa05164 | Influenza A | 17/138 | 171/8586 | 1.64E-09 | 2.58E-08 | 1.95E-08 | 3553/3115/3122/3117/3113/3119/3123/3118/3108/60/114548/293/9021/3576/3109/637/29108 | 17 |
| hsa05320 | Autoimmune thyroid disease | 10/138 | 53/8586 | 8.98E-09 | 1.32E-07 | 9.95E-08 | 3115/3122/3117/3113/3119/3123/3118/3108/942/3109 | 10 |
| hsa05150 | Staphylococcus aureus infection | 12/138 | 96/8586 | 3.63E-08 | 4.99E-07 | 3.77E-07 | 3115/3122/3117/3113/3119/3123/3118/3108/2212/728/2213/3109 | 12 |
| hsa05321 | Inflammatory bowel disease | 10/138 | 65/8586 | 7.00E-08 | 9.05E-07 | 6.84E-07 | 3553/3115/3122/3117/3113/3119/3123/3118/3108/3109 | 10 |
| hsa04659 | Th17 cell differentiation | 12/138 | 108/8586 | 1.37E-07 | 1.68E-06 | 1.27E-06 | 3553/3115/3122/3117/3113/3119/3123/3118/3108/3109/196/3091 | 12 |
| hsa05145 | Toxoplasmosis | 12/138 | 111/8586 | 1.87E-07 | 2.16E-06 | 1.63E-06 | 3115/3122/3117/3113/3119/3123/3118/3108/10105/3109/3303/3310 | 12 |
| hsa03010 | Ribosome | 14/138 | 167/8586 | 4.19E-07 | 4.60E-06 | 3.48E-06 | 6205/6229/9349/6203/6202/6207/6154/6228/6129/7311/6218/6189/6173/6142 | 14 |
| hsa05169 | Epstein-Barr virus infection | 15/138 | 202/8586 | 7.61E-07 | 7.97E-06 | 6.02E-06 | 3115/3122/3117/3113/3119/3123/3118/3108/7431/1026/3109/637/4067/4616/960 | 15 |
| hsa05417 | Lipid and atherosclerosis | 15/138 | 215/8586 | 1.68E-06 | 1.68E-05 | 1.27E-05 | 3553/6648/4973/2920/114548/3576/1536/637/29108/4067/3303/3329/3310/6349/6348 | 15 |
| hsa05322 | Systemic lupus erythematosus | 11/138 | 137/8586 | 1.17E-05 | 0.000111813 | 8.45E-05 | 3115/3122/3117/3113/3119/3123/3118/3108/2212/942/3109 | 11 |
| hsa05166 | Human T-cell leukemia virus 1 infection | 14/138 | 222/8586 | 1.22E-05 | 0.000112104 | 8.47E-05 | 3115/3122/3117/3113/3119/3123/3118/3108/1026/2114/6688/293/3109/706 | 14 |
| hsa04658 | Th1 and Th2 cell differentiation | 9/138 | 92/8586 | 1.55E-05 | 0.000136113 | 0.000102899 | 3115/3122/3117/3113/3119/3123/3118/3108/3109 | 9 |
| hsa05134 | Legionellosis | 7/138 | 56/8586 | 2.86E-05 | 0.000242049 | 0.000182984 | 3553/2920/3576/29108/3303/3329/3310 | 7 |
| hsa04514 | Cell adhesion molecules | 11/138 | 158/8586 | 4.46E-05 | 0.000363006 | 0.000274425 | 3115/3122/3117/3113/3119/3123/1462/3118/3108/942/3109 | 11 |
| hsa05167 | Kaposi sarcoma-associated herpesvirus infection | 12/138 | 194/8586 | 6.37E-05 | 0.000500522 | 0.000378385 | 2920/7422/1026/942/3576/7311/1499/637/3091/5743/4067/7316 | 12 |
| hsa05146 | Amoebiasis | 8/138 | 102/8586 | 0.0002238 | 0.001697565 | 0.001283327 | 3553/2920/911/3315/5272/913/3576/7879 | 8 |
| hsa04064 | NF-kappa B signaling pathway | 8/138 | 104/8586 | 0.0002559 | 0.001876282 | 0.001418433 | 3553/2920/597/3576/10673/5743/4067/4616 | 8 |
| hsa04216 | Ferroptosis | 5/138 | 41/8586 | 0.0004695 | 0.003227671 | 0.002440058 | 6303/2495/2180/1536/3162 | 5 |
| hsa05219 | Bladder cancer | 5/138 | 41/8586 | 0.0004695 | 0.003227671 | 0.002440058 | 7422/1890/1839/1026/3576 | 5 |
| hsa04657 | IL-17 signaling pathway | 7/138 | 94/8586 | 0.0007608 | 0.005071992 | 0.003834329 | 3553/6280/2920/1051/6279/3576/5743 | 7 |
| hsa04625 | C-type lectin receptor signaling pathway | 7/138 | 104/8586 | 0.0013826 | 0.008946505 | 0.006763387 | 3553/2207/114548/64581/4046/29108/5743 | 7 |
| hsa05418 | Fluid shear stress and atherosclerosis | 8/138 | 139/8586 | 0.0017484 | 0.010989933 | 0.008308179 | 3553/7422/2950/60/7056/4208/1499/3162 | 8 |
| hsa05132 | Salmonella infection | 11/138 | 249/8586 | 0.0021894 | 0.013379455 | 0.010114612 | 3553/60/6281/114548/8767/388/3576/7879/1499/29108/10094 | 11 |
| hsa04621 | NOD-like receptor signaling pathway | 9/138 | 186/8586 | 0.0030124 | 0.017911646 | 0.013540862 | 3553/2920/114548/8767/11337/10135/3576/1536/29108 | 9 |
| hsa05202 | Transcriptional misregulation in cancer | 9/138 | 193/8586 | 0.0038507 | 0.022293791 | 0.016853679 | 597/8013/1051/1026/6688/942/3576/4208/4616 | 9 |
| hsa04217 | Necroptosis | 8/138 | 159/8586 | 0.004027 | 0.022716395 | 0.01717316 | 3553/2495/114548/293/57132/1536/637/29108 | 8 |
| hsa04137 | Mitophagy - animal | 5/138 | 72/8586 | 0.0058951 | 0.032085382 | 0.024255935 | 11337/7879/7311/3091/7316 | 5 |
| hsa04380 | Osteoclast differentiation | 7/138 | 135/8586 | 0.0059795 | 0.032085382 | 0.024255935 | 3553/2212/6688/7305/2213/9021/10288 | 7 |
| hsa04210 | Apoptosis | 7/138 | 136/8586 | 0.0062215 | 0.032353041 | 0.02445828 | 1520/597/60/1512/1522/637/4616 | 7 |
| hsa05131 | Shigellosis | 10/138 | 247/8586 | 0.0063756 | 0.032353041 | 0.02445828 | 3553/60/114548/8767/3576/7311/29108/10094/7316/960 | 10 |
| hsa05135 | Yersinia infection | 7/138 | 137/8586 | 0.0064706 | 0.032353041 | 0.02445828 | 3553/60/114548/2212/3576/29108/10094 | 7 |
| hsa03320 | PPAR signaling pathway | 5/138 | 75/8586 | 0.0069996 | 0.034220447 | 0.025870003 | 4973/2180/2710/7316/123 | 5 |
| hsa04066 | HIF-1 signaling pathway | 6/138 | 109/8586 | 0.0080976 | 0.038727875 | 0.029277532 | 7076/7422/1026/1536/3091/3162 | 6 |
| hsa05163 | Human cytomegalovirus infection | 9/138 | 225/8586 | 0.0102432 | 0.047946819 | 0.036246878 | 3553/7422/1026/3576/1499/637/5743/6349/6348 | 9 |
| hsa04650 | Natural killer cell mediated cytotoxicity | 14/124 | 132/8586 | 5.33E-09 | 1.26E-06 | 8.86E-07 | 3824/5295/3821/919/22914/3823/2534/7462/4068/5594/5551/117157/10870/3458 | 14 |
| hsa05145 | Toxoplasmosis | 12/124 | 111/8586 | 5.67E-08 | 6.72E-06 | 4.72E-06 | 3310/3303/4790/54205/3716/5594/329/3458/3312/8651/4792/7040 | 12 |
| hsa04659 | Th17 cell differentiation | 11/124 | 108/8586 | 3.85E-07 | 2.48E-05 | 1.74E-05 | 3560/3320/4790/3326/919/3716/5594/2625/3458/4792/7040 | 11 |
| hsa05417 | Lipid and atherosclerosis | 15/124 | 215/8586 | 4.19E-07 | 2.48E-05 | 1.74E-05 | 3310/3329/6348/5295/3303/3320/6352/4790/3326/54205/5594/801/3312/4792/2081 | 15 |
| hsa04658 | Th1 and Th2 cell differentiation | 10/124 | 92/8586 | 7.27E-07 | 3.45E-05 | 2.42E-05 | 3560/4790/919/864/3716/5594/6775/2625/3458/4792 | 10 |
| hsa04612 | Antigen processing and presentation | 9/124 | 78/8586 | 1.61E-06 | 6.36E-05 | 4.46E-05 | 3310/3824/3303/3320/3821/3326/3823/3458/3312 | 9 |
| hsa04620 | Toll-like receptor signaling pathway | 10/124 | 108/8586 | 3.22E-06 | 0.000101856 | 7.15E-05 | 6351/6348/5295/9560/6352/4790/3716/1326/5594/4792 | 10 |
| hsa04380 | Osteoclast differentiation | 11/124 | 135/8586 | 3.60E-06 | 0.000101856 | 7.15E-05 | 5295/3727/4790/2534/2355/3716/5594/3458/8651/4792/7040 | 11 |
| hsa04210 | Apoptosis | 11/124 | 136/8586 | 3.87E-06 | 0.000101856 | 7.15E-05 | 5295/4790/54205/7277/1521/5594/5551/329/4792/2081/4616 | 11 |
| hsa05162 | Measles | 11/124 | 138/8586 | 4.46E-06 | 0.000105702 | 7.42E-05 | 3310/5295/3560/3303/4790/54205/894/3716/3312/4792/9367 | 11 |
| hsa05134 | Legionellosis | 7/124 | 56/8586 | 1.43E-05 | 0.000298647 | 0.000209577 | 3310/3329/3303/4790/54205/3312/4792 | 7 |
| hsa05142 | Chagas disease | 9/124 | 102/8586 | 1.51E-05 | 0.000298647 | 0.000209577 | 6348/5295/6352/4790/919/5594/3458/4792/7040 | 9 |
| hsa05163 | Human cytomegalovirus infection | 13/124 | 225/8586 | 2.04E-05 | 0.000371201 | 0.000260492 | 6351/6348/5295/9560/6352/4790/54205/54331/3716/5594/801/11214/4792 | 13 |
| hsa05167 | Kaposi sarcoma-associated herpesvirus infection | 12/124 | 194/8586 | 2.20E-05 | 0.0003717 | 0.000260842 | 5295/4790/54205/7538/7314/54331/3716/5594/801/4792/9976/7316 | 12 |
| hsa04061 | Viral protein interaction with cytokine and cytokine receptor | 8/124 | 100/8586 | 9.25E-05 | 0.001456053 | 0.001021792 | 6846/6351/6375/6348/3560/9560/6352/8740 | 8 |
| hsa04062 | Chemokine signaling pathway | 11/124 | 192/8586 | 9.83E-05 | 0.001456053 | 0.001021792 | 6846/6351/6375/6348/5295/9560/6352/4790/54331/5594/4792 | 11 |
| hsa04915 | Estrogen signaling pathway | 9/124 | 137/8586 | 0.0001549 | 0.002159228 | 0.001515248 | 3310/5295/3303/3320/3326/2288/5594/801/3312 | 9 |
| hsa04141 | Protein processing in endoplasmic reticulum | 10/124 | 170/8586 | 0.000166 | 0.002185289 | 0.001533536 | 3310/3303/3320/10808/3326/3337/3301/3312/2081/23645 | 10 |
| hsa04668 | TNF signaling pathway | 8/124 | 114/8586 | 0.0002312 | 0.00288438 | 0.002024126 | 5295/6352/4790/3659/1326/5594/329/4792 | 8 |
| hsa05235 | PD-L1 expression and PD-1 checkpoint pathway in cancer | 7/124 | 89/8586 | 0.0002858 | 0.003386989 | 0.002376834 | 5295/4790/919/3716/5594/3458/4792 | 7 |
| hsa04660 | T cell receptor signaling pathway | 8/124 | 121/8586 | 0.0003476 | 0.003922752 | 0.002752809 | 5295/4790/919/2534/1326/5594/3458/4792 | 8 |
| hsa04010 | MAPK signaling pathway | 13/124 | 301/8586 | 0.000388 | 0.004122664 | 0.002893098 | 3310/374/3303/3727/4790/1844/1326/5594/3312/2872/3164/7040/4616 | 13 |
| hsa04657 | IL-17 signaling pathway | 7/124 | 94/8586 | 0.0004001 | 0.004122664 | 0.002893098 | 3320/3727/4790/3326/5594/3458/4792 | 7 |
| hsa05202 | Transcriptional misregulation in cancer | 10/124 | 193/8586 | 0.0004615 | 0.004515727 | 0.003168931 | 5966/3560/4790/894/7704/1655/329/64332/3398/4616 | 10 |
| hsa05215 | Prostate cancer | 7/124 | 97/8586 | 0.0004845 | 0.004515727 | 0.003168931 | 5295/3320/4790/3326/2950/5594/4792 | 7 |
| hsa04917 | Prolactin signaling pathway | 6/124 | 70/8586 | 0.0004954 | 0.004515727 | 0.003168931 | 5295/4790/3659/894/5594/8651 | 6 |
| hsa05161 | Hepatitis B | 9/124 | 162/8586 | 0.00054 | 0.004739797 | 0.003326173 | 5295/4790/54205/3716/5594/6775/7534/4792/7040 | 9 |
| hsa04623 | Cytosolic DNA-sensing pathway | 6/124 | 75/8586 | 0.0007175 | 0.00588603 | 0.004130547 | 6351/9560/6352/4790/5440/4792 | 6 |
| hsa04064 | NF-kappa B signaling pathway | 7/124 | 104/8586 | 0.0007373 | 0.00588603 | 0.004130547 | 6351/9560/4790/8740/329/4792/4616 | 7 |
| hsa05212 | Pancreatic cancer | 6/124 | 76/8586 | 0.0007699 | 0.00588603 | 0.004130547 | 5295/4790/3716/5594/7040/4616 | 6 |
| hsa05220 | Chronic myeloid leukemia | 6/124 | 76/8586 | 0.0007699 | 0.00588603 | 0.004130547 | 5295/4790/5594/4792/7040/4616 | 6 |
| hsa05164 | Influenza A | 9/124 | 171/8586 | 0.0007977 | 0.005907791 | 0.004145818 | 5295/6352/4790/54205/3337/3716/5594/3458/4792 | 9 |
| hsa05140 | Leishmaniasis | 6/124 | 77/8586 | 0.0008252 | 0.005926723 | 0.004159104 | 4790/3716/5594/3458/4792/7040 | 6 |
| hsa05166 | Human T-cell leukemia virus 1 infection | 10/124 | 222/8586 | 0.0013592 | 0.009474646 | 0.006648874 | 5295/3560/4790/894/2113/7538/3716/5594/4792/7040 | 10 |
| hsa04621 | NOD-like receptor signaling pathway | 9/124 | 186/8586 | 0.0014441 | 0.009696209 | 0.006804357 | 3320/6352/4790/3326/3716/5594/23710/329/4792 | 9 |
| hsa05210 | Colorectal cancer | 6/124 | 86/8586 | 0.0014728 | 0.009696209 | 0.006804357 | 374/5295/54205/5594/7040/4616 | 6 |
| hsa04218 | Cellular senescence | 8/124 | 156/8586 | 0.0018413 | 0.01166855 | 0.008188456 | 5295/4790/894/2113/5594/801/7040/4616 | 8 |
| hsa05020 | Prion disease | 11/124 | 272/8586 | 0.0018709 | 0.01166855 | 0.008188456 | 3310/5295/3303/6352/54205/7277/2534/10963/5594/4684/3312 | 11 |
| hsa05160 | Hepatitis C | 8/124 | 158/8586 | 0.0019954 | 0.012125945 | 0.008509435 | 5295/4790/54205/3716/5594/7534/3458/4792 | 8 |
| hsa05222 | Small cell lung cancer | 6/124 | 92/8586 | 0.0020826 | 0.012339151 | 0.008659054 | 5295/4790/54205/329/4792/4616 | 6 |
| hsa05321 | Inflammatory bowel disease | 5/124 | 65/8586 | 0.0023964 | 0.013852489 | 0.009721045 | 4790/6775/2625/3458/7040 | 5 |
| hsa05169 | Epstein-Barr virus infection | 9/124 | 202/8586 | 0.0025432 | 0.014351082 | 0.010070935 | 5295/4790/919/54205/894/864/3716/4792/4616 | 9 |
| hsa05131 | Shigellosis | 10/124 | 247/8586 | 0.0029827 | 0.016351457 | 0.011474707 | 5295/6352/4790/54205/7314/5594/81873/4792/989/7316 | 10 |
| hsa05332 | Graft-versus-host disease | 4/124 | 42/8586 | 0.0030357 | 0.016351457 | 0.011474707 | 3824/3821/5551/3458 | 4 |
| hsa05132 | Salmonella infection | 10/124 | 249/8586 | 0.0031607 | 0.016646441 | 0.011681713 | 3320/4790/3326/54205/7277/5594/81873/329/4792/9367 | 10 |
| hsa05170 | Human immunodeficiency virus 1 infection | 9/124 | 212/8586 | 0.0035139 | 0.018104459 | 0.012704883 | 5295/4790/919/54205/60489/54331/5594/801/4792 | 9 |
| hsa04625 | C-type lectin receptor signaling pathway | 6/124 | 104/8586 | 0.0038528 | 0.019236209 | 0.013499094 | 5295/4790/3659/5594/801/4792 | 6 |
| hsa05418 | Fluid shear stress and atherosclerosis | 7/124 | 139/8586 | 0.0039125 | 0.019236209 | 0.013499094 | 5295/3320/4790/3326/2950/801/3458 | 7 |
| hsa01524 | Platinum drug resistance | 5/124 | 73/8586 | 0.0039771 | 0.019236209 | 0.013499094 | 5295/54205/2950/5594/329 | 5 |
| hsa04928 | Parathyroid hormone synthesis, secretion and action | 6/124 | 106/8586 | 0.0042318 | 0.020058772 | 0.014076331 | 3727/5142/5594/2625/4929/11214 | 6 |
| hsa05152 | Tuberculosis | 8/124 | 180/8586 | 0.0044526 | 0.020691308 | 0.014520216 | 3329/4790/54205/3716/5594/801/3458/7040 | 8 |
| hsa05133 | Pertussis | 5/124 | 76/8586 | 0.0047272 | 0.021545194 | 0.015119434 | 4790/3659/3394/5594/801 | 5 |
| hsa04151 | PI3K-Akt signaling pathway | 12/124 | 359/8586 | 0.0055832 | 0.024966398 | 0.017520279 | 374/5295/3560/3320/4790/3326/894/54331/3716/5594/7534/3164 | 12 |
| hsa05144 | Malaria | 4/124 | 50/8586 | 0.0057172 | 0.025092034 | 0.017608445 | 3820/22914/3458/7040 | 4 |
| hsa04662 | B cell receptor signaling pathway | 5/124 | 84/8586 | 0.0072115 | 0.031074885 | 0.021806937 | 5295/4790/8519/5594/4792 | 5 |
| hsa04722 | Neurotrophin signaling pathway | 6/124 | 119/8586 | 0.0074009 | 0.031321663 | 0.021980114 | 5295/4790/4145/5594/801/4792 | 6 |
| hsa05130 | Pathogenic Escherichia coli infection | 8/124 | 198/8586 | 0.0078161 | 0.032498556 | 0.022806004 | 4790/54205/7277/2534/5594/81873/4691/4792 | 8 |
| hsa05203 | Viral carcinogenesis | 8/124 | 204/8586 | 0.0092809 | 0.037923686 | 0.026613113 | 5966/5295/4790/894/3716/5594/7534/4792 | 8 |
| hsa04630 | JAK-STAT signaling pathway | 7/124 | 166/8586 | 0.0101146 | 0.040630014 | 0.028512291 | 5295/3560/894/3716/6775/3458/8651 | 7 |
| hsa04060 | Cytokine-cytokine receptor interaction | 10/124 | 297/8586 | 0.0106398 | 0.041974608 | 0.029455865 | 6846/6351/6375/6348/3560/9560/6352/8740/3458/7040 | 10 |
| hsa04926 | Relaxin signaling pathway | 6/124 | 129/8586 | 0.0108036 | 0.041974608 | 0.029455865 | 5295/4790/54331/5594/4792/7040 | 6 |
| hsa04213 | Longevity regulating pathway - multiple species | 4/124 | 61/8586 | 0.0114769 | 0.04363193 | 0.030618898 | 3310/5295/3303/3312 | 4 |
| hsa04068 | FoxO signaling pathway | 6/124 | 131/8586 | 0.0115984 | 0.04363193 | 0.030618898 | 5295/894/5594/23710/7040/4616 | 6 |
| hsa03010 | Ribosome | 55/86 | 167/8586 | 6.15E-76 | 7.38E-74 | 6.99E-74 | 6231/6164/6170/6235/6232/6206/6210/6176/6233/6204/6171/6227/6173/6165/6139/6154/6223/6169/6202/6161/6144/9045/6234/6156/6158/6136/4736/6135/25873/6207/6230/6147/6167/6222/6181/6152/6146/11224/6194/6228/6160/3921/6155/6168/6128/6201/6133/6137/6209/7311/6224/6166/2197/6193/6191 | 55 |
| hsa05171 | Coronavirus disease - COVID-19 | 56/86 | 232/8586 | 8.91E-69 | 5.34E-67 | 5.06E-67 | 6231/6164/6170/6235/6232/6206/6210/6176/6233/6204/6171/6227/6173/6165/6139/6154/6223/6169/6202/6161/6144/9045/6234/6156/6158/6136/4736/6135/25873/6207/6230/6147/6167/6222/6181/6152/6146/11224/6194/6228/6160/3921/6155/6168/6128/6201/6133/6137/6209/7311/6224/6166/2197/6193/6191/7124 | 56 |
| hsa04640 | Hematopoietic cell lineage | 6/86 | 99/8586 | 0.0004509 | 0.018035214 | 0.017085993 | 914/3575/915/917/7124/960 | 6 |
| hsa05162 | Measles | 13/117 | 138/8586 | 4.03E-08 | 7.25E-06 | 5.42E-06 | 915/3559/894/3561/917/916/6504/940/596/355/9367/3716/6774 | 13 |
| hsa05169 | Epstein-Barr virus infection | 15/117 | 202/8586 | 8.52E-08 | 7.67E-06 | 5.74E-06 | 915/1647/894/917/567/3105/916/596/355/953/3134/10912/3716/6774/3516 | 15 |
| hsa00190 | Oxidative phosphorylation | 12/117 | 134/8586 | 2.42E-07 | 1.45E-05 | 1.09E-05 | 514/1350/10063/4707/51079/4697/10632/517/1351/7381/1347/4729 | 12 |
| hsa05340 | Primary immunodeficiency | 7/117 | 38/8586 | 6.48E-07 | 2.44E-05 | 1.83E-05 | 29851/915/3561/916/3575/5788/3932 | 7 |
| hsa04660 | T cell receptor signaling pathway | 11/117 | 121/8586 | 6.79E-07 | 2.44E-05 | 1.83E-05 | 1493/29851/915/917/916/10125/940/8440/5788/3702/3932 | 11 |
| hsa05166 | Human T-cell leukemia virus 1 infection | 14/117 | 222/8586 | 1.73E-06 | 5.18E-05 | 3.87E-05 | 915/3559/894/2113/3561/917/567/3105/916/9232/119504/3932/3134/3716 | 14 |
| hsa05235 | PD-L1 expression and PD-1 checkpoint pathway in cancer | 9/117 | 89/8586 | 3.03E-06 | 7.80E-05 | 5.84E-05 | 10538/915/917/916/10125/940/3932/3716/6774 | 9 |
| hsa05415 | Diabetic cardiomyopathy | 13/117 | 203/8586 | 3.51E-06 | 7.89E-05 | 5.90E-05 | 9882/514/2597/1350/5880/4707/51079/4697/517/1351/7381/1347/4729 | 13 |
| hsa04658 | Th1 and Th2 cell differentiation | 9/117 | 92/8586 | 4.01E-06 | 8.01E-05 | 6.00E-05 | 915/3559/3561/917/916/4094/3932/3716/3516 | 9 |
| hsa04659 | Th17 cell differentiation | 9/117 | 108/8586 | 1.50E-05 | 0.000270647 | 0.00020259 | 915/50943/3559/3561/917/916/3932/3716/6774 | 9 |
| hsa05170 | Human immunodeficiency virus 1 infection | 12/117 | 212/8586 | 2.95E-05 | 0.000482953 | 0.000361509 | 915/917/567/3105/916/596/355/5880/3134/7133/6923/6500 | 12 |
| hsa04115 | p53 signaling pathway | 7/117 | 74/8586 | 6.17E-05 | 0.000925327 | 0.000692642 | 1647/894/5366/596/3732/355/10912 | 7 |
| hsa04714 | Thermogenesis | 12/117 | 232/8586 | 7.12E-05 | 0.000986207 | 0.000738213 | 514/1350/10063/4707/51079/4697/10632/517/1351/7381/1347/4729 | 12 |
| hsa05014 | Amyotrophic lateral sclerosis | 15/117 | 364/8586 | 0.0001174 | 0.001509783 | 0.00113013 | 6647/514/1350/596/5216/10010/4707/51079/4697/517/7133/1351/7381/1347/4729 | 15 |
| hsa05208 | Chemical carcinogenesis - reactive oxygen species | 11/117 | 223/8586 | 0.0002193 | 0.002631004 | 0.001969407 | 6647/514/1350/4707/51079/4697/517/1351/7381/1347/4729 | 11 |
| hsa04932 | Non-alcoholic fatty liver disease | 9/117 | 155/8586 | 0.0002531 | 0.002739484 | 0.002050608 | 1350/355/4707/51079/4697/1351/7381/1347/4729 | 9 |
| hsa05012 | Parkinson disease | 12/117 | 266/8586 | 0.0002587 | 0.002739484 | 0.002050608 | 6647/514/1350/11315/4707/51079/4697/517/1351/7381/1347/4729 | 12 |
| hsa04940 | Type I diabetes mellitus | 5/117 | 43/8586 | 0.0002745 | 0.002745311 | 0.00205497 | 3105/3382/940/355/3134 | 5 |
| hsa05020 | Prion disease | 12/117 | 272/8586 | 0.0003175 | 0.003007919 | 0.002251541 | 6647/514/1350/5880/4707/51079/4697/517/1351/7381/1347/4729 | 12 |
| hsa04640 | Hematopoietic cell lineage | 7/117 | 99/8586 | 0.0003857 | 0.003471408 | 0.002598481 | 914/915/3559/917/916/3575/924 | 7 |
| hsa05022 | Pathways of neurodegeneration - multiple diseases | 16/117 | 476/8586 | 0.000692 | 0.005931766 | 0.004440152 | 6647/514/1350/596/355/10010/11315/4707/51079/4697/517/7133/1351/7381/1347/4729 | 16 |
| hsa05320 | Autoimmune thyroid disease | 5/117 | 53/8586 | 0.0007344 | 0.006009061 | 0.00449801 | 1493/3105/940/355/3134 | 5 |
| hsa04514 | Cell adhesion molecules | 8/117 | 158/8586 | 0.0013771 | 0.010776946 | 0.008066953 | 201633/1493/914/29851/3105/940/5788/3134 | 8 |
| hsa05330 | Allograft rejection | 4/117 | 38/8586 | 0.0016908 | 0.012681199 | 0.009492359 | 3105/940/355/3134 | 4 |
| hsa05203 | Viral carcinogenesis | 9/117 | 204/8586 | 0.0018237 | 0.013050313 | 0.009768656 | 894/3105/5366/5315/3134/7529/3716/6774/3516 | 9 |
| hsa04630 | JAK-STAT signaling pathway | 8/117 | 166/8586 | 0.001885 | 0.013050313 | 0.009768656 | 3559/894/3561/8027/3575/596/3716/6774 | 8 |
| hsa05132 | Salmonella infection | 10/117 | 249/8586 | 0.0020608 | 0.013738336 | 0.010283667 | 330/2597/596/5216/5788/10095/9367/399/1781/6500 | 10 |
| hsa04060 | Cytokine-cytokine receptor interaction | 11/117 | 297/8586 | 0.0023478 | 0.015015931 | 0.011239995 | 7293/8784/9235/4050/939/3604/3559/3561/3575/355/7133 | 11 |
| hsa05332 | Graft-versus-host disease | 4/117 | 42/8586 | 0.0024589 | 0.015015931 | 0.011239995 | 3105/940/355/3134 | 4 |
| hsa04210 | Apoptosis | 7/117 | 136/8586 | 0.0025027 | 0.015015931 | 0.011239995 | 1075/1647/330/5366/596/355/10912 | 7 |
| hsa04064 | NF-kappa B signaling pathway | 6/117 | 104/8586 | 0.0028879 | 0.016644618 | 0.012459129 | 4050/1647/330/596/3932/10912 | 6 |
| hsa05016 | Huntington disease | 11/117 | 306/8586 | 0.002959 | 0.016644618 | 0.012459129 | 6647/514/1350/4707/51079/4697/517/1351/7381/1347/4729 | 11 |
| hsa03050 | Proteasome | 4/117 | 46/8586 | 0.0034384 | 0.018754855 | 0.014038722 | 5698/5696/5720/5721 | 4 |
| hsa05212 | Pancreatic cancer | 5/117 | 76/8586 | 0.0036899 | 0.019534957 | 0.014622658 | 1647/5880/10912/3716/6774 | 5 |
| hsa04612 | Antigen processing and presentation | 5/117 | 78/8586 | 0.0041268 | 0.021223735 | 0.015886772 | 567/3105/3134/5720/5721 | 5 |
| hsa04218 | Cellular senescence | 7/117 | 156/8586 | 0.005344 | 0.026719892 | 0.020000855 | 1647/894/2113/3105/2308/3134/10912 | 7 |
| hsa04110 | Cell cycle | 7/117 | 157/8586 | 0.0055317 | 0.026910804 | 0.020143759 | 1647/894/9232/119504/7529/10912/6500 | 7 |
| hsa05010 | Alzheimer disease | 12/117 | 384/8586 | 0.0059509 | 0.028188347 | 0.021100049 | 514/2597/1350/355/4707/51079/4697/517/1351/7381/1347/4729 | 12 |
| hsa05210 | Colorectal cancer | 5/117 | 86/8586 | 0.006248 | 0.028836974 | 0.021585571 | 1647/5366/596/5880/10912 | 5 |
| hsa04068 | FoxO signaling pathway | 6/117 | 131/8586 | 0.0088518 | 0.038999107 | 0.029192314 | 1647/894/3575/2308/10912/6774 | 6 |
| hsa05416 | Viral myocarditis | 4/117 | 60/8586 | 0.0088831 | 0.038999107 | 0.029192314 | 3105/940/5880/3134 | 4 |
| hsa04215 | Apoptosis - multiple species | 3/117 | 32/8586 | 0.0091742 | 0.039317808 | 0.029430874 | 330/5366/596 | 3 |
| hsa05321 | Inflammatory bowel disease | 4/117 | 65/8586 | 0.0117113 | 0.049023843 | 0.03669621 | 50943/3561/4094/6774 | 4 |
| hsa04064 | NF-kappa B signaling pathway | 12/110 | 104/8586 | 6.88E-09 | 1.47E-06 | 1.10E-06 | 3576/597/5743/3553/7128/4792/2919/5328/4067/3383/7132/7099 | 12 |
| hsa05417 | Lipid and atherosclerosis | 15/110 | 215/8586 | 8.42E-08 | 8.96E-06 | 6.73E-06 | 3576/6648/3553/4780/4688/2353/4792/2919/7097/4067/5606/653361/3383/7132/7099 | 15 |
| hsa04668 | TNF signaling pathway | 11/110 | 114/8586 | 1.97E-07 | 1.24E-05 | 9.28E-06 | 5743/1051/3553/7128/2353/4792/2919/7133/5606/3383/7132 | 11 |
| hsa05323 | Rheumatoid arthritis | 10/110 | 93/8586 | 2.62E-07 | 1.24E-05 | 9.28E-06 | 3576/3553/2353/2919/7422/7097/527/3383/7099/533 | 10 |
| hsa04657 | IL-17 signaling pathway | 10/110 | 94/8586 | 2.90E-07 | 1.24E-05 | 9.28E-06 | 3576/6279/6280/5743/1051/3553/7128/2353/4792/2919 | 10 |
| hsa05140 | Leishmaniasis | 9/110 | 77/8586 | 5.22E-07 | 1.85E-05 | 1.39E-05 | 5743/2215/3553/4688/2353/4792/7097/653361/7099 | 9 |
| hsa05418 | Fluid shear stress and atherosclerosis | 11/110 | 139/8586 | 1.46E-06 | 4.45E-05 | 3.34E-05 | 3553/1843/4780/4688/7850/2353/7422/653361/7056/3383/7132 | 11 |
| hsa05152 | Tuberculosis | 11/110 | 180/8586 | 1.78E-05 | 0.000474492 | 0.000356426 | 2215/1051/3553/26253/7097/527/3687/1520/7132/7099/533 | 11 |
| hsa05120 | Epithelial cell signaling in Helicobacter pylori infection | 7/110 | 70/8586 | 2.88E-05 | 0.000682752 | 0.000512865 | 3576/3579/4792/2919/4067/527/533 | 7 |
| hsa05167 | Kaposi sarcoma-associated herpesvirus infection | 11/110 | 194/8586 | 3.58E-05 | 0.000762384 | 0.000572683 | 3576/5743/2353/4792/2919/3091/81631/7422/4067/3383/7132 | 11 |
| hsa04380 | Osteoclast differentiation | 9/110 | 135/8586 | 5.45E-05 | 0.001055844 | 0.000793122 | 2215/3553/3937/4688/2353/4792/2355/653361/7132 | 9 |
| hsa05134 | Legionellosis | 6/110 | 56/8586 | 7.44E-05 | 0.001320409 | 0.000991857 | 3576/3553/4792/2919/7097/7099 | 6 |
| hsa04936 | Alcoholic liver disease | 9/110 | 142/8586 | 8.09E-05 | 0.001325186 | 0.000995445 | 3576/728/3553/4792/2919/2309/5606/7132/7099 | 9 |
| hsa04613 | Neutrophil extracellular trap formation | 10/110 | 191/8586 | 0.0001598 | 0.002431217 | 0.001826266 | 728/2357/2215/366/10105/4688/2358/7097/653361/7099 | 10 |
| hsa05142 | Chagas disease | 7/110 | 102/8586 | 0.0003177 | 0.004510665 | 0.003388293 | 3576/3553/2353/4792/7097/7132/7099 | 7 |
| hsa05135 | Yersinia infection | 8/110 | 137/8586 | 0.0003574 | 0.004758252 | 0.003574274 | 3576/3553/3937/2353/4792/5606/7099/8935 | 8 |
| hsa05144 | Malaria | 5/110 | 50/8586 | 0.0004219 | 0.005285697 | 0.003970476 | 3576/3553/7097/3383/7099 | 5 |
| hsa04620 | Toll-like receptor signaling pathway | 7/110 | 108/8586 | 0.0004504 | 0.005329871 | 0.004003659 | 3576/3553/2353/4792/7097/5606/7099 | 7 |
| hsa05208 | Chemical carcinogenesis - reactive oxygen species | 10/110 | 223/8586 | 0.0005538 | 0.006208662 | 0.004663784 | 6648/10105/4780/4688/2353/4792/2309/3091/7422/653361 | 10 |
| hsa04621 | NOD-like receptor signaling pathway | 9/110 | 186/8586 | 0.0006122 | 0.006520448 | 0.004897989 | 3576/10135/3553/7128/4792/2919/81631/11337/7099 | 9 |
| hsa04145 | Phagosome | 8/110 | 152/8586 | 0.0007153 | 0.007255371 | 0.005450044 | 2215/4688/7097/527/653361/1520/7099/533 | 8 |
| hsa04610 | Complement and coagulation cascades | 6/110 | 86/8586 | 0.0007896 | 0.007644742 | 0.005742529 | 728/5329/1604/5328/3687/7056 | 6 |
| hsa05235 | PD-L1 expression and PD-1 checkpoint pathway in cancer | 6/110 | 89/8586 | 0.0009462 | 0.00876233 | 0.006582032 | 2353/4792/3091/7097/5606/7099 | 6 |
| hsa04142 | Lysosome | 7/110 | 132/8586 | 0.0014812 | 0.013145897 | 0.009874852 | 9516/6556/427/7805/527/1520/533 | 7 |
| hsa04061 | Viral protein interaction with cytokine and cytokine receptor | 6/110 | 100/8586 | 0.0017328 | 0.014083082 | 0.010578841 | 3576/3579/2919/7133/7852/7132 | 6 |
| hsa04216 | Ferroptosis | 4/110 | 41/8586 | 0.0017932 | 0.014083082 | 0.010578841 | 2495/2180/6303/81631 | 4 |
| hsa04920 | Adipocytokine signaling pathway | 5/110 | 69/8586 | 0.0018459 | 0.014083082 | 0.010578841 | 2180/8660/4792/7133/7132 | 5 |
| hsa05162 | Measles | 7/110 | 138/8586 | 0.0019128 | 0.014083082 | 0.010578841 | 3553/7128/2353/4600/4792/7097/7099 | 7 |
| hsa05146 | Amoebiasis | 6/110 | 102/8586 | 0.0019174 | 0.014083082 | 0.010578841 | 3576/3553/7850/2919/7097/7099 | 6 |
| hsa05171 | Coronavirus disease - COVID-19 | 9/110 | 232/8586 | 0.0028586 | 0.02019333 | 0.015168698 | 3576/728/3553/2353/4600/4792/7097/7132/7099 | 9 |
| hsa05145 | Toxoplasmosis | 6/110 | 111/8586 | 0.0029389 | 0.02019333 | 0.015168698 | 10105/4792/7097/5606/7132/7099 | 6 |
| hsa04062 | Chemokine signaling pathway | 8/110 | 192/8586 | 0.0031653 | 0.021069113 | 0.015826564 | 3576/3579/4792/2919/2309/7852/4067/653361 | 8 |
| hsa05202 | Transcriptional misregulation in cancer | 8/110 | 193/8586 | 0.0032677 | 0.021091296 | 0.015843227 | 3576/597/1051/7850/8013/5328/604/64332 | 8 |
| hsa04060 | Cytokine-cytokine receptor interaction | 10/110 | 297/8586 | 0.0046608 | 0.029198314 | 0.021933006 | 3576/1441/3553/5008/7850/3579/2919/7133/7852/7132 | 10 |
| hsa05170 | Human immunodeficiency virus 1 infection | 8/110 | 212/8586 | 0.0057493 | 0.034988531 | 0.026282465 | 2353/4792/7097/7133/7852/5606/7132/7099 | 8 |
| hsa05164 | Influenza A | 7/110 | 171/8586 | 0.0062721 | 0.037110122 | 0.027876148 | 3576/3553/4600/4792/3383/7132/7099 | 7 |
| hsa05150 | Staphylococcus aureus infection | 5/110 | 96/8586 | 0.0076493 | 0.043342639 | 0.032557851 | 728/2357/2215/2358/3383 | 5 |
| hsa04210 | Apoptosis | 6/110 | 136/8586 | 0.0078926 | 0.043342639 | 0.032557851 | 597/4170/2353/4792/1520/7132 | 6 |
| hsa04666 | Fc gamma R-mediated phagocytosis | 5/110 | 97/8586 | 0.0079846 | 0.043342639 | 0.032557851 | 2215/4082/4067/653361/7408 | 5 |
| hsa05163 | Human cytomegalovirus infection | 8/110 | 225/8586 | 0.0081395 | 0.043342639 | 0.032557851 | 3576/5743/3553/3579/4792/7422/7852/7132 | 8 |
| hsa03250 | Viral life cycle - HIV-1 | 4/110 | 63/8586 | 0.0085083 | 0.044047369 | 0.033087226 | 22936/4600/7852/10955 | 4 |
| hsa04640 | Hematopoietic cell lineage | 5/110 | 99/8586 | 0.0086854 | 0.044047369 | 0.033087226 | 1604/1441/3553/7850/4311 | 5 |
| hsa04933 | AGE-RAGE signaling pathway in diabetic complications | 5/110 | 100/8586 | 0.0090513 | 0.044835634 | 0.03367935 | 3576/3553/7422/7056/3383 | 5 |
| hsa00190 | Oxidative phosphorylation | 19/136 | 134/8586 | 2.11E-13 | 4.94E-11 | 3.89E-11 | 1345/27089/4697/7381/517/522/1337/29796/4718/9551/1340/4707/521/7388/506/518/1329/4726/1349 | 19 |
| hsa05208 | Chemical carcinogenesis - reactive oxygen species | 22/136 | 223/8586 | 4.41E-12 | 4.44E-10 | 3.49E-10 | 2938/1345/4257/2052/27089/4697/7381/517/522/1337/29796/4718/1340/4707/292/7388/506/5879/518/1329/4726/1349 | 22 |
| hsa05415 | Diabetic cardiomyopathy | 21/136 | 203/8586 | 5.69E-12 | 4.44E-10 | 3.49E-10 | 5166/183/1345/27089/4697/7381/517/522/1337/29796/4718/1340/4707/292/7388/506/5879/518/1329/4726/1349 | 21 |
| hsa05020 | Prion disease | 23/136 | 272/8586 | 3.36E-11 | 1.96E-09 | 1.55E-09 | 713/712/714/1345/27089/4697/10376/7381/517/522/1337/29796/4718/1340/4707/292/7388/506/5879/518/1329/4726/1349 | 23 |
| hsa04610 | Complement and coagulation cascades | 14/136 | 86/8586 | 5.48E-11 | 2.56E-09 | 2.02E-09 | 713/712/462/7448/714/3827/629/5265/2266/2244/2243/2/710/966 | 14 |
| hsa04612 | Antigen processing and presentation | 13/136 | 78/8586 | 2.03E-10 | 7.92E-09 | 6.24E-09 | 3122/3113/3115/3123/972/3117/5641/1508/3108/3119/10437/1520/821 | 13 |
| hsa04979 | Cholesterol metabolism | 11/136 | 51/8586 | 2.91E-10 | 9.73E-09 | 7.66E-09 | 345/341/335/348/344/336/338/350/27329/3988/10577 | 11 |
| hsa05012 | Parkinson disease | 21/136 | 266/8586 | 9.42E-10 | 2.75E-08 | 2.17E-08 | 1345/7295/27089/4697/10376/7381/805/517/522/1337/29796/4718/1340/4707/292/7388/506/518/1329/4726/1349 | 21 |
| hsa05150 | Staphylococcus aureus infection | 13/136 | 96/8586 | 2.92E-09 | 7.59E-08 | 5.98E-08 | 3122/3113/713/712/3115/714/3123/3117/629/2266/1672/3108/3119 | 13 |
| hsa04714 | Thermogenesis | 19/136 | 232/8586 | 3.50E-09 | 8.18E-08 | 6.44E-08 | 1345/27089/4697/7381/517/522/1337/29796/4718/9551/1340/4707/521/7388/506/518/1329/4726/1349 | 19 |
| hsa05310 | Asthma | 8/136 | 31/8586 | 1.87E-08 | 3.98E-07 | 3.13E-07 | 3122/3113/3115/3123/3117/3108/3119/2207 | 8 |
| hsa04932 | Non-alcoholic fatty liver disease | 14/136 | 155/8586 | 1.38E-07 | 2.69E-06 | 2.12E-06 | 1345/27089/4697/7381/1337/29796/4718/1340/4707/7388/5879/1329/4726/1349 | 14 |
| hsa05014 | Amyotrophic lateral sclerosis | 21/136 | 364/8586 | 2.34E-07 | 4.12E-06 | 3.24E-06 | 283/1345/27089/4697/10376/7381/400916/517/522/1337/29796/4718/1340/4707/7388/506/5879/518/1329/4726/1349 | 21 |
| hsa03320 | PPAR signaling pathway | 10/136 | 75/8586 | 2.46E-07 | 4.12E-06 | 3.24E-06 | 345/2168/335/5105/3158/336/6319/2171/123/6342 | 10 |
| hsa05016 | Huntington disease | 19/136 | 306/8586 | 3.03E-07 | 4.73E-06 | 3.73E-06 | 1345/27089/4697/10376/7381/517/522/1337/29796/4718/1340/4707/292/7388/506/518/1329/4726/1349 | 19 |
| hsa05010 | Alzheimer disease | 21/136 | 384/8586 | 5.69E-07 | 8.33E-06 | 6.55E-06 | 348/1345/27089/4697/10376/7381/805/517/522/1337/29796/4718/1340/4707/292/7388/506/518/1329/4726/1349 | 21 |
| hsa05330 | Allograft rejection | 7/136 | 38/8586 | 1.80E-06 | 2.47E-05 | 1.95E-05 | 3122/3113/3115/3123/3117/3108/3119 | 7 |
| hsa05332 | Graft-versus-host disease | 7/136 | 42/8586 | 3.64E-06 | 4.73E-05 | 3.73E-05 | 3122/3113/3115/3123/3117/3108/3119 | 7 |
| hsa05416 | Viral myocarditis | 8/136 | 60/8586 | 4.12E-06 | 5.02E-05 | 3.95E-05 | 3122/3113/3115/3123/3117/3108/3119/5879 | 8 |
| hsa04940 | Type I diabetes mellitus | 7/136 | 43/8586 | 4.29E-06 | 5.02E-05 | 3.95E-05 | 3122/3113/3115/3123/3117/3108/3119 | 7 |
| hsa04142 | Lysosome | 11/136 | 132/8586 | 7.11E-06 | 7.92E-05 | 6.24E-05 | 3988/5660/10577/5641/967/1508/968/1520/1522/427/5538 | 11 |
| hsa04260 | Cardiac muscle contraction | 9/136 | 87/8586 | 8.68E-06 | 9.23E-05 | 7.27E-05 | 1345/27089/7381/1337/29796/1340/7388/1329/1349 | 9 |
| hsa04672 | Intestinal immune network for IgA production | 7/136 | 49/8586 | 1.06E-05 | 0.000107553 | 8.47E-05 | 3122/3113/3115/3123/3117/3108/3119 | 7 |
| hsa05204 | Chemical carcinogenesis - DNA adducts | 8/136 | 70/8586 | 1.33E-05 | 0.000129883 | 0.000102247 | 1576/1549/6822/2938/7363/4257/2052/2950 | 8 |
| hsa05323 | Rheumatoid arthritis | 9/136 | 93/8586 | 1.50E-05 | 0.000140571 | 0.000110661 | 3122/3113/3115/3123/3117/3108/3119/2920/6348 | 9 |
| hsa05022 | Pathways of neurodegeneration - multiple diseases | 21/136 | 476/8586 | 1.70E-05 | 0.000152722 | 0.000120227 | 1345/27089/4697/10376/7381/805/517/522/1337/29796/4718/1340/4707/292/7388/506/5879/518/1329/4726/1349 | 21 |
| hsa05320 | Autoimmune thyroid disease | 7/136 | 53/8586 | 1.80E-05 | 0.000155966 | 0.00012278 | 3122/3113/3115/3123/3117/3108/3119 | 7 |
| hsa04145 | Phagosome | 11/136 | 152/8586 | 2.72E-05 | 0.000227007 | 0.000178705 | 3122/3113/3115/3123/3117/3108/3119/1520/10376/821/5879 | 11 |
| hsa00980 | Metabolism of xenobiotics by cytochrome P450 | 8/136 | 78/8586 | 2.98E-05 | 0.000240054 | 0.000188976 | 1576/1549/6822/2938/7363/4257/2052/2950 | 8 |
| hsa04216 | Ferroptosis | 6/136 | 41/8586 | 4.03E-05 | 0.000314458 | 0.000247549 | 7018/30061/2512/1356/8031/2879 | 6 |
| hsa05322 | Systemic lupus erythematosus | 10/136 | 137/8586 | 5.93E-05 | 0.000447718 | 0.000352454 | 3122/3113/713/712/3115/714/3123/3117/3108/3119 | 10 |
| hsa05321 | Inflammatory bowel disease | 7/136 | 65/8586 | 6.94E-05 | 0.000507535 | 0.000399544 | 3122/3113/3115/3123/3117/3108/3119 | 7 |
| hsa05152 | Tuberculosis | 11/136 | 180/8586 | 0.000127 | 0.000900493 | 0.00070889 | 3122/3113/3115/3123/972/3117/3108/3119/2207/1520/805 | 11 |
| hsa04640 | Hematopoietic cell lineage | 8/136 | 99/8586 | 0.0001644 | 0.001131548 | 0.000890782 | 3122/3113/3115/3123/3117/3108/3119/966 | 8 |
| hsa05140 | Leishmaniasis | 7/136 | 77/8586 | 0.0002048 | 0.001369112 | 0.001077799 | 3122/3113/3115/3123/3117/3108/3119 | 7 |
| hsa00983 | Drug metabolism - other enzymes | 7/136 | 80/8586 | 0.0002601 | 0.001690468 | 0.001330778 | 1576/1549/2938/7363/4257/4831/2950 | 7 |
| hsa05145 | Toxoplasmosis | 8/136 | 111/8586 | 0.0003617 | 0.002287683 | 0.00180092 | 3122/3113/3115/3123/3117/3108/3119/23643 | 8 |
| hsa04658 | Th1 and Th2 cell differentiation | 7/136 | 92/8586 | 0.0006128 | 0.00377379 | 0.00297082 | 3122/3113/3115/3123/3117/3108/3119 | 7 |
| hsa00982 | Drug metabolism - cytochrome P450 | 6/136 | 72/8586 | 0.0009378 | 0.00562688 | 0.004429618 | 1576/1549/2938/7363/4257/2950 | 6 |
| hsa05133 | Pertussis | 6/136 | 76/8586 | 0.0012463 | 0.007290741 | 0.00573945 | 713/712/714/710/805/23643 | 6 |
| hsa05169 | Epstein-Barr virus infection | 10/136 | 202/8586 | 0.0013473 | 0.00768955 | 0.006053402 | 3122/3113/3115/3123/3117/3108/3119/3280/10912/5879 | 10 |
| hsa04659 | Th17 cell differentiation | 7/136 | 108/8586 | 0.0015823 | 0.008815929 | 0.006940115 | 3122/3113/3115/3123/3117/3108/3119 | 7 |
| hsa04936 | Alcoholic liver disease | 8/136 | 142/8586 | 0.0018248 | 0.009930435 | 0.007817482 | 2168/713/712/714/6319/2920/23643/217 | 8 |
| hsa04978 | Mineral absorption | 5/136 | 60/8586 | 0.0025168 | 0.013384578 | 0.010536667 | 7018/30061/2512/4502/475 | 5 |
| hsa00830 | Retinol metabolism | 5/136 | 68/8586 | 0.0043439 | 0.022588094 | 0.0177819 | 1576/1549/8630/7363/216 | 5 |
| hsa05164 | Influenza A | 8/136 | 171/8586 | 0.0057047 | 0.029019614 | 0.02284495 | 3122/3113/3115/3123/3117/3108/3119/292 | 8 |
| hsa05166 | Human T-cell leukemia virus 1 infection | 9/136 | 222/8586 | 0.0086061 | 0.042847594 | 0.033730675 | 3122/3113/3115/3123/3117/3108/3119/821/292 | 9 |
| hsa04141 | Protein processing in endoplasmic reticulum | 23/54 | 170/8586 | 9.36E-26 | 1.37E-23 | 1.25E-23 | 6748/91319/81567/7494/27248/9709/55829/7184/51465/3998/6747/746/6238/64374/6400/10952/23480/9601/7095/10130/10960/468/3310 | 23 |
| hsa03060 | Protein export | 8/54 | 23/8586 | 6.50E-13 | 4.75E-11 | 4.35E-11 | 90701/9789/60559/28972/58477/10952/23480/7095 | 8 |
| hsa05110 | Vibrio cholerae infection | 4/54 | 50/8586 | 0.0002598 | 0.012641319 | 0.011574964 | 10952/10945/23480/9601 | 4 |
| hsa04918 | Thyroid hormone synthesis | 4/54 | 75/8586 | 0.0012207 | 0.042123049 | 0.038569771 | 7184/64764/9601/468 | 4 |
| hsa04915 | Estrogen signaling pathway | 5/54 | 137/8586 | 0.0016237 | 0.042123049 | 0.038569771 | 7184/64764/3725/468/3310 | 5 |
| hsa05418 | Fluid shear stress and atherosclerosis | 5/54 | 139/8586 | 0.0017311 | 0.042123049 | 0.038569771 | 6382/7184/1535/3725/10365 | 5 |
| hsa05417 | Lipid and atherosclerosis | 6/54 | 215/8586 | 0.0021788 | 0.04544303 | 0.041609696 | 7494/7184/1535/3725/468/3310 | 6 |
| hsa04976 | Bile secretion | 11/104 | 89/8586 | 8.02E-09 | 1.79E-06 | 1.40E-06 | 486/358/1080/760/8671/6523/7366/8714/3949/481/476 | 11 |
| hsa04530 | Tight junction | 13/104 | 170/8586 | 1.16E-07 | 1.30E-05 | 1.02E-05 | 1365/1364/9071/9076/1080/4301/595/1366/9223/3688/3725/103910/71 | 13 |
| hsa04964 | Proximal tubule bicarbonate reclamation | 6/104 | 23/8586 | 2.33E-07 | 1.73E-05 | 1.36E-05 | 486/358/760/8671/481/476 | 6 |
| hsa04670 | Leukocyte transendothelial migration | 10/104 | 115/8586 | 1.14E-06 | 6.34E-05 | 4.97E-05 | 1365/1364/9071/9076/4301/1366/1500/3688/103910/71 | 10 |
| hsa05418 | Fluid shear stress and atherosclerosis | 10/104 | 139/8586 | 6.39E-06 | 0.000285074 | 0.000223377 | 6385/858/857/7422/2950/2353/3725/1843/805/71 | 10 |
| hsa04514 | Cell adhesion molecules | 10/104 | 158/8586 | 1.98E-05 | 0.000736372 | 0.000577001 | 1365/1364/9071/9076/1366/6385/999/1000/3696/3688 | 10 |
| hsa04972 | Pancreatic secretion | 8/104 | 102/8586 | 3.03E-05 | 0.000852369 | 0.000667894 | 486/6558/1080/760/8671/1361/481/476 | 8 |
| hsa04510 | Focal adhesion | 11/104 | 203/8586 | 3.21E-05 | 0.000852369 | 0.000667894 | 7448/595/858/3696/857/7057/7422/3688/3725/103910/71 | 11 |
| hsa04971 | Gastric acid secretion | 7/104 | 76/8586 | 3.44E-05 | 0.000852369 | 0.000667894 | 1080/760/3773/481/476/805/71 | 7 |
| hsa04015 | Rap1 signaling pathway | 11/104 | 210/8586 | 4.39E-05 | 0.000978351 | 0.00076661 | 2263/4301/2261/999/9223/7057/7422/1500/3688/805/71 | 11 |
| hsa04610 | Complement and coagulation cascades | 7/104 | 86/8586 | 7.66E-05 | 0.001552602 | 0.001216577 | 1191/710/7448/5104/1361/966/5265 | 7 |
| hsa05219 | Bladder cancer | 5/104 | 41/8586 | 0.0001255 | 0.002312368 | 0.00181191 | 595/2261/999/7057/7422 | 5 |
| hsa04657 | IL-17 signaling pathway | 7/104 | 94/8586 | 0.0001348 | 0.002312368 | 0.00181191 | 6372/2919/2353/3725/2354/2920/3727 | 7 |
| hsa05133 | Pertussis | 6/104 | 76/8586 | 0.0003008 | 0.004790766 | 0.003753916 | 6372/710/2353/3688/3725/805 | 6 |
| hsa05412 | Arrhythmogenic right ventricular cardiomyopathy | 6/104 | 77/8586 | 0.000323 | 0.004802173 | 0.003762854 | 1832/1000/3696/1829/3688/71 | 6 |
| hsa05130 | Pathogenic Escherichia coli infection | 9/104 | 198/8586 | 0.0006381 | 0.00889308 | 0.00696838 | 1365/1364/9071/9076/1366/2353/3688/3725/71 | 9 |
| hsa04978 | Mineral absorption | 5/104 | 60/8586 | 0.0007622 | 0.009998636 | 0.007834664 | 486/4493/6523/481/476 | 5 |
| hsa05205 | Proteoglycans in cancer | 9/104 | 205/8586 | 0.0008186 | 0.010141788 | 0.007946834 | 7448/595/6385/858/857/7057/7422/3688/71 | 9 |
| hsa05323 | Rheumatoid arthritis | 6/104 | 93/8586 | 0.0008886 | 0.01042921 | 0.00817205 | 6372/2919/7422/2353/3725/2920 | 6 |
| hsa04024 | cAMP signaling pathway | 9/104 | 225/8586 | 0.0015748 | 0.017558759 | 0.013758574 | 486/6662/1080/4301/481/476/2353/3725/805 | 9 |
| hsa05100 | Bacterial invasion of epithelial cells | 5/104 | 77/8586 | 0.0023447 | 0.023553686 | 0.018456039 | 858/999/857/3688/71 | 5 |
| hsa05167 | Kaposi sarcoma-associated herpesvirus infection | 8/104 | 194/8586 | 0.0023777 | 0.023553686 | 0.018456039 | 2919/595/7422/2353/3725/805/2920/7538 | 8 |
| hsa04973 | Carbohydrate digestion and absorption | 4/104 | 47/8586 | 0.0024293 | 0.023553686 | 0.018456039 | 486/6523/481/476 | 4 |
| hsa05160 | Hepatitis C | 7/104 | 158/8586 | 0.0029882 | 0.027765388 | 0.021756216 | 1365/1364/9071/9076/595/1366/3949 | 7 |
| hsa04512 | ECM-receptor interaction | 5/104 | 89/8586 | 0.0043947 | 0.039200629 | 0.030716566 | 7448/6385/3696/7057/3688 | 5 |
| hsa05410 | Hypertrophic cardiomyopathy | 5/104 | 90/8586 | 0.0046092 | 0.039533102 | 0.030977082 | 183/7168/3696/3688/71 | 5 |
| hsa04520 | Adherens junction | 5/104 | 93/8586 | 0.0052976 | 0.042191685 | 0.033060277 | 4301/999/1500/103910/71 | 5 |
| hsa04970 | Salivary secretion | 5/104 | 93/8586 | 0.0052976 | 0.042191685 | 0.033060277 | 486/6558/481/476/805 | 5 |
| hsa05416 | Viral myocarditis | 4/104 | 60/8586 | 0.0058914 | 0.045015329 | 0.035272809 | 595/1525/857/71 | 4 |
| hsa05414 | Dilated cardiomyopathy | 5/104 | 96/8586 | 0.0060559 | 0.045015329 | 0.035272809 | 183/7168/3696/3688/71 | 5 |
| hsa04925 | Aldosterone synthesis and secretion | 5/104 | 98/8586 | 0.006602 | 0.046111496 | 0.036131736 | 183/3949/481/476/805 | 5 |
| hsa00140 | Steroid hormone biosynthesis | 4/104 | 62/8586 | 0.0066169 | 0.046111496 | 0.036131736 | 1577/1645/3294/7366 | 4 |
| hsa04933 | AGE-RAGE signaling pathway in diabetic complications | 5/104 | 100/8586 | 0.0071818 | 0.048531481 | 0.038027972 | 183/595/7422/1958/3725 | 5 |
