# Supplementary material for: Prognostic risk model of LIHC T-cells based on scRNA-seq and RNA-seq and the regulation of the tumor immune microenvironment
Source: Discov Oncol. 2024 Oct 10;15:540. doi: 10.1007/s12672-024-01424-z (PMC11467143; doi:10.1007/s12672-024-01424-z)
Supplement: Supplementary file 4 — Supplementary material 4. [file 12672_2024_1424_MOESM4_ESM.doc]

| **Supplementary Table 3 Differential genes after screening** | | | | | | |
| --- | --- | --- | --- | --- | --- | --- |
|  | baseMean | log2FoldChange | lfcSE | stat | pvalue | padj |
| CDKN3 | 372.9955 | 4.026011 | 0.189996 | 21.18997 | 1.18E-99 | 6.38E-96 |
| UBE2T | 374.1753 | 3.313764 | 0.158469 | 20.91106 | 4.25E-97 | 1.15E-93 |
| NUF2 | 275.5627 | 4.320362 | 0.209382 | 20.63383 | 1.36E-94 | 2.45E-91 |
| CENPF | 1008.804 | 4.015222 | 0.194808 | 20.6112 | 2.18E-94 | 2.94E-91 |
| TROAP | 391.4894 | 4.343965 | 0.211849 | 20.50499 | 1.94E-93 | 2.10E-90 |
| KIFC1 | 644.8226 | 3.876841 | 0.192067 | 20.18488 | 1.33E-90 | 1.20E-87 |
| KIF4A | 468.4539 | 4.032002 | 0.200255 | 20.13433 | 3.69E-90 | 2.85E-87 |
| BIRC5 | 1037.067 | 4.201768 | 0.20898 | 20.10607 | 6.53E-90 | 4.41E-87 |
| HJURP | 340.1049 | 4.007988 | 0.20065 | 19.97497 | 9.09E-89 | 5.45E-86 |
| CDCA5 | 534.9917 | 3.553464 | 0.178135 | 19.94818 | 1.55E-88 | 8.39E-86 |
| CCNB2 | 427.1095 | 3.833794 | 0.193714 | 19.79104 | 3.56E-87 | 1.75E-84 |
| GPC3 | 29546.31 | 6.050124 | 0.305848 | 19.78145 | 4.30E-87 | 1.79E-84 |
| MELK | 291.7799 | 3.903969 | 0.197343 | 19.78266 | 4.20E-87 | 1.79E-84 |
| CDC20 | 807.636 | 4.392039 | 0.22263 | 19.72796 | 1.24E-86 | 4.78E-84 |
| KIF2C | 424.2079 | 4.04926 | 0.206455 | 19.61328 | 1.19E-85 | 4.29E-83 |
| NCAPG | 408.6133 | 3.902537 | 0.199027 | 19.60811 | 1.32E-85 | 4.45E-83 |
| ASPM | 830.8271 | 3.829758 | 0.19541 | 19.59856 | 1.59E-85 | 5.05E-83 |
| TOP2A | 1819.415 | 4.013607 | 0.205922 | 19.49087 | 1.31E-84 | 3.94E-82 |
| PRC1 | 773.1555 | 3.328557 | 0.170845 | 19.48295 | 1.53E-84 | 4.24E-82 |
| MXD3 | 438.5333 | 2.788994 | 0.14316 | 19.48162 | 1.57E-84 | 4.24E-82 |
| CCNB1 | 919.4291 | 3.387365 | 0.175418 | 19.31025 | 4.40E-83 | 1.13E-80 |
| PTTG1 | 728.2589 | 3.83932 | 0.199272 | 19.26675 | 1.02E-82 | 2.51E-80 |
| UBE2C | 661.3591 | 4.241141 | 0.220388 | 19.24394 | 1.59E-82 | 3.72E-80 |
| NDC80 | 330.5842 | 3.421347 | 0.179111 | 19.10182 | 2.44E-81 | 5.49E-79 |
| CDK1 | 631.2883 | 3.59351 | 0.188462 | 19.06756 | 4.70E-81 | 1.01E-78 |
| MYBL2 | 972.555 | 4.687064 | 0.246635 | 19.00407 | 1.58E-80 | 3.28E-78 |
| BUB1 | 336.1233 | 3.610159 | 0.190215 | 18.97934 | 2.53E-80 | 4.87E-78 |
| CENPA | 134.5681 | 4.232737 | 0.223006 | 18.98035 | 2.48E-80 | 4.87E-78 |
| DLGAP5 | 253.2643 | 3.817587 | 0.201296 | 18.96502 | 3.32E-80 | 6.18E-78 |
| FOXM1 | 717.3603 | 3.715687 | 0.19925 | 18.64836 | 1.30E-77 | 2.34E-75 |
| CDCA3 | 324.0299 | 3.402665 | 0.185313 | 18.36169 | 2.66E-75 | 4.64E-73 |
| CDKN2A | 699.5948 | 4.170648 | 0.227183 | 18.35813 | 2.84E-75 | 4.80E-73 |
| PLK1 | 484.821 | 3.652873 | 0.199392 | 18.32009 | 5.72E-75 | 9.36E-73 |
| CDCA8 | 358.6422 | 3.273582 | 0.179997 | 18.18687 | 6.56E-74 | 1.04E-71 |
| HMMR | 371.0426 | 3.336475 | 0.183609 | 18.1716 | 8.66E-74 | 1.34E-71 |
| ANLN | 496.5011 | 3.743968 | 0.206107 | 18.16517 | 9.74E-74 | 1.46E-71 |
| PKMYT1 | 261.7531 | 3.278785 | 0.181898 | 18.02541 | 1.23E-72 | 1.80E-70 |
| AURKB | 362.9437 | 3.683296 | 0.207044 | 17.78989 | 8.46E-71 | 1.20E-68 |
| EZH2 | 518.6177 | 2.526214 | 0.142064 | 17.78218 | 9.71E-71 | 1.34E-68 |
| RACGAP1 | 635.6994 | 2.532764 | 0.142502 | 17.77359 | 1.13E-70 | 1.53E-68 |
| CDT1 | 544.9886 | 3.533172 | 0.198814 | 17.77127 | 1.18E-70 | 1.55E-68 |
| RRM2 | 1200.021 | 3.166357 | 0.178404 | 17.7482 | 1.78E-70 | 2.29E-68 |
| MKI67 | 1328.842 | 3.470293 | 0.195743 | 17.72879 | 2.51E-70 | 3.16E-68 |
| DTL | 472.8437 | 3.380526 | 0.190694 | 17.72752 | 2.57E-70 | 3.16E-68 |
| AURKA | 803.9368 | 2.803932 | 0.158819 | 17.65484 | 9.34E-70 | 1.12E-67 |
| SPC25 | 135.3346 | 3.248646 | 0.184138 | 17.64247 | 1.16E-69 | 1.36E-67 |
| BUB1B | 262.4953 | 3.629266 | 0.205765 | 17.63794 | 1.26E-69 | 1.45E-67 |
| GTSE1 | 221.7363 | 3.660855 | 0.207731 | 17.62308 | 1.64E-69 | 1.84E-67 |
| CDC6 | 560.8695 | 3.319289 | 0.189566 | 17.50996 | 1.20E-68 | 1.33E-66 |
| DEPDC1 | 216.1995 | 3.8793 | 0.222111 | 17.46561 | 2.62E-68 | 2.83E-66 |
| CENPM | 297.5725 | 3.647379 | 0.209171 | 17.43728 | 4.30E-68 | 4.55E-66 |
| IGF2BP1 | 651.8703 | 7.133281 | 0.410822 | 17.36345 | 1.56E-67 | 1.62E-65 |
| CENPE | 222.0419 | 3.249362 | 0.187252 | 17.35286 | 1.88E-67 | 1.91E-65 |
| PBK | 251.0326 | 3.507176 | 0.206392 | 16.99276 | 9.29E-65 | 9.29E-63 |
| KIF23 | 273.4796 | 3.411974 | 0.201718 | 16.91455 | 3.51E-64 | 3.45E-62 |
| NCAPH | 245.3701 | 3.082171 | 0.183386 | 16.80702 | 2.17E-63 | 2.09E-61 |
| CKAP2L | 203.9772 | 3.310501 | 0.19715 | 16.79179 | 2.80E-63 | 2.65E-61 |
| MND1 | 110.9716 | 3.042944 | 0.181457 | 16.76952 | 4.08E-63 | 3.80E-61 |
| CCNA2 | 559.2196 | 3.602181 | 0.214863 | 16.76501 | 4.40E-63 | 4.03E-61 |
| KIF14 | 193.0199 | 3.218423 | 0.192371 | 16.73031 | 7.88E-63 | 7.09E-61 |
| MDK | 7263.797 | 3.928674 | 0.235565 | 16.67767 | 1.91E-62 | 1.69E-60 |
| TCF19 | 772.1282 | 2.886987 | 0.174307 | 16.56268 | 1.30E-61 | 1.13E-59 |
| TRIM71 | 356.5125 | 8.745396 | 0.533121 | 16.40413 | 1.79E-60 | 1.53E-58 |
| SPATS2 | 727.6873 | 1.691733 | 0.103167 | 16.39802 | 1.98E-60 | 1.67E-58 |
| SGO1 | 126.8034 | 3.160206 | 0.192965 | 16.37713 | 2.79E-60 | 2.31E-58 |
| ASF1B | 401.0101 | 3.097963 | 0.189689 | 16.33181 | 5.86E-60 | 4.80E-58 |
| SHCBP1 | 166.1033 | 3.4169 | 0.210375 | 16.24197 | 2.55E-59 | 2.05E-57 |
| KIF15 | 155.5961 | 3.474439 | 0.214224 | 16.2187 | 3.72E-59 | 2.95E-57 |
| TMEM120B | 432.6733 | 1.371497 | 0.08502 | 16.13154 | 1.53E-58 | 1.20E-56 |
| DIAPH3 | 148.3528 | 3.494892 | 0.217522 | 16.06685 | 4.36E-58 | 3.36E-56 |
| DUSP9 | 1176.025 | 5.16285 | 0.32156 | 16.05565 | 5.22E-58 | 3.97E-56 |
| SPDL1 | 285.6809 | 1.923039 | 0.120792 | 15.92026 | 4.58E-57 | 3.44E-55 |
| GMNN | 1706.691 | 2.317392 | 0.145804 | 15.89392 | 6.98E-57 | 5.16E-55 |
| CSRNP1 | 1907.35 | -2.14262 | 0.135291 | -15.8371 | 1.72E-56 | 1.26E-54 |
| MMP11 | 794.0755 | 3.928628 | 0.248809 | 15.7897 | 3.66E-56 | 2.64E-54 |
| ARHGAP11A | 347.5362 | 2.655022 | 0.168906 | 15.71891 | 1.12E-55 | 7.97E-54 |
| ZWINT | 725.7241 | 2.572544 | 0.16497 | 15.59403 | 7.99E-55 | 5.60E-53 |
| UHRF1 | 261.7557 | 3.34302 | 0.215224 | 15.53277 | 2.08E-54 | 1.44E-52 |
| CENPW | 264.8428 | 2.708161 | 0.174403 | 15.5282 | 2.24E-54 | 1.53E-52 |
| CFP | 398.9105 | -3.28163 | 0.211704 | -15.501 | 3.41E-54 | 2.30E-52 |
| FANCI | 540.728 | 2.376361 | 0.153314 | 15.50001 | 3.47E-54 | 2.31E-52 |
| ORC6 | 138.6505 | 2.970837 | 0.193078 | 15.38674 | 2.01E-53 | 1.32E-51 |
| POLQ | 121.5805 | 3.117718 | 0.202948 | 15.36219 | 2.93E-53 | 1.91E-51 |
| CDCA2 | 102.7414 | 3.297408 | 0.21606 | 15.26154 | 1.38E-52 | 8.87E-51 |
| MAP2K1 | 1972.024 | -1.15807 | 0.076335 | -15.1708 | 5.52E-52 | 3.50E-50 |
| SGO2 | 184.9724 | 2.495344 | 0.164792 | 15.14235 | 8.51E-52 | 5.34E-50 |
| CEP55 | 171.7587 | 3.182119 | 0.211063 | 15.07666 | 2.31E-51 | 1.43E-49 |
| STMN1 | 3334.388 | 2.231218 | 0.14818 | 15.05749 | 3.08E-51 | 1.89E-49 |
| TNFRSF4 | 157.7869 | 2.428393 | 0.161352 | 15.0503 | 3.44E-51 | 2.08E-49 |
| NDUFA4L2 | 1145.549 | 2.911249 | 0.193519 | 15.04371 | 3.80E-51 | 2.28E-49 |
| NUSAP1 | 957.6243 | 2.37445 | 0.159275 | 14.90784 | 2.93E-50 | 1.74E-48 |
| PCLAF | 391.4779 | 2.578874 | 0.173567 | 14.85812 | 6.16E-50 | 3.62E-48 |
| RFC4 | 674.5695 | 1.855407 | 0.124924 | 14.85224 | 6.73E-50 | 3.91E-48 |
| CDKN2C | 772.7069 | 2.401361 | 0.16213 | 14.81133 | 1.24E-49 | 7.11E-48 |
| SCAMP3 | 4709.962 | 1.395556 | 0.095317 | 14.64116 | 1.53E-48 | 8.72E-47 |
| CENPK | 113.6592 | 2.732594 | 0.186964 | 14.61561 | 2.23E-48 | 1.26E-46 |
| ECT2 | 550.2552 | 2.516088 | 0.172214 | 14.61023 | 2.42E-48 | 1.35E-46 |
| RND3 | 2277.728 | -2.37341 | 0.16262 | -14.5948 | 3.03E-48 | 1.67E-46 |
| TK1 | 1517.543 | 2.434548 | 0.168382 | 14.45844 | 2.22E-47 | 1.21E-45 |
| CTHRC1 | 330.4912 | 4.103341 | 0.284048 | 14.44595 | 2.66E-47 | 1.44E-45 |
| FEN1 | 1267.867 | 1.742715 | 0.120684 | 14.44037 | 2.88E-47 | 1.54E-45 |
| MAD2L1 | 345.5258 | 2.268832 | 0.157933 | 14.36583 | 8.48E-47 | 4.49E-45 |
| CENPH | 189.1785 | 1.962461 | 0.136692 | 14.35679 | 9.66E-47 | 5.06E-45 |
| PEA15 | 5057.394 | 1.288749 | 0.090423 | 14.25246 | 4.33E-46 | 2.25E-44 |
| TACC3 | 1219.06 | 2.23111 | 0.156798 | 14.22917 | 6.04E-46 | 3.11E-44 |
| KIF11 | 356.2018 | 2.306137 | 0.16327 | 14.12465 | 2.68E-45 | 1.36E-43 |
| ILF2 | 5481.756 | 1.082344 | 0.076809 | 14.09133 | 4.29E-45 | 2.17E-43 |
| PRR11 | 257.8867 | 2.600193 | 0.185948 | 13.98347 | 1.97E-44 | 9.83E-43 |
| HELLS | 323.0943 | 2.522882 | 0.181068 | 13.93332 | 3.97E-44 | 1.97E-42 |
| CENPU | 416.6703 | 2.290935 | 0.16464 | 13.91479 | 5.15E-44 | 2.53E-42 |
| ROBO1 | 2910.64 | 3.001507 | 0.215771 | 13.91064 | 5.46E-44 | 2.66E-42 |
| MCM6 | 1331.958 | 1.855984 | 0.133436 | 13.90914 | 5.57E-44 | 2.69E-42 |
| AFP | 15498.24 | 6.223334 | 0.449245 | 13.85288 | 1.22E-43 | 5.84E-42 |
| ZFP36 | 7344.38 | -2.043 | 0.147975 | -13.8064 | 2.33E-43 | 1.10E-41 |
| CCT3 | 15520.94 | 1.287707 | 0.093534 | 13.76721 | 4.01E-43 | 1.88E-41 |
| GBA | 3538.026 | 1.48836 | 0.108121 | 13.76567 | 4.10E-43 | 1.91E-41 |
| CHAF1A | 744.2341 | 1.577535 | 0.114645 | 13.76023 | 4.42E-43 | 2.04E-41 |
| WDR76 | 279.0303 | 2.336694 | 0.170363 | 13.71596 | 8.15E-43 | 3.70E-41 |
| RAB3B | 577.012 | 3.880502 | 0.284636 | 13.63323 | 2.54E-42 | 1.14E-40 |
| S100P | 2407.46 | 5.767306 | 0.423173 | 13.62872 | 2.70E-42 | 1.21E-40 |
| FAM111B | 314.8568 | 2.70286 | 0.198829 | 13.59387 | 4.35E-42 | 1.93E-40 |
| MCM3 | 3060.071 | 1.665374 | 0.122701 | 13.57267 | 5.82E-42 | 2.55E-40 |
| NQO1 | 7892.029 | 4.897326 | 0.361958 | 13.53011 | 1.04E-41 | 4.52E-40 |
| DDX39A | 2460.677 | 1.591923 | 0.11792 | 13.50005 | 1.56E-41 | 6.75E-40 |
| MUC13 | 5945.083 | 5.290798 | 0.393444 | 13.44739 | 3.19E-41 | 1.37E-39 |
| COL2A1 | 377.9929 | 8.115498 | 0.603716 | 13.44257 | 3.40E-41 | 1.45E-39 |
| RAD51AP1 | 170.0917 | 2.376302 | 0.177875 | 13.35939 | 1.04E-40 | 4.40E-39 |
| TRIB1 | 5696.496 | -1.88257 | 0.140932 | -13.358 | 1.06E-40 | 4.45E-39 |
| NT5DC2 | 1139.132 | 2.777093 | 0.209433 | 13.26004 | 3.95E-40 | 1.64E-38 |
| IGF2BP3 | 152.3206 | 4.868161 | 0.367978 | 13.22949 | 5.93E-40 | 2.44E-38 |
| MARCO | 737.2414 | -4.75019 | 0.359161 | -13.2258 | 6.23E-40 | 2.55E-38 |
| UBE2S | 705.4161 | 2.099075 | 0.159022 | 13.19989 | 8.79E-40 | 3.57E-38 |
| C1RL | 7068.325 | -1.54084 | 0.119 | -12.9482 | 2.40E-38 | 9.69E-37 |
| LCN2 | 9988.376 | 4.848067 | 0.375003 | 12.92807 | 3.13E-38 | 1.25E-36 |
| NELFE | 3162.065 | 1.465136 | 0.113475 | 12.91152 | 3.88E-38 | 1.53E-36 |
| KIAA1522 | 3235.106 | 1.627958 | 0.126563 | 12.86279 | 7.29E-38 | 2.85E-36 |
| TMEM106C | 2895.103 | 1.607224 | 0.125121 | 12.84534 | 9.14E-38 | 3.55E-36 |
| SMARCA4 | 3369.367 | 1.066113 | 0.083394 | 12.78398 | 2.01E-37 | 7.77E-36 |
| CLSPN | 79.99571 | 2.916215 | 0.228224 | 12.77788 | 2.18E-37 | 8.34E-36 |
| TPX2 | 1260.066 | 2.23193 | 0.175651 | 12.70663 | 5.43E-37 | 2.07E-35 |
| CETP | 452.3862 | -2.6611 | 0.209731 | -12.6882 | 6.88E-37 | 2.60E-35 |
| NUDT1 | 466.9028 | 1.944279 | 0.154201 | 12.60872 | 1.89E-36 | 7.04E-35 |
| CHEK1 | 342.3527 | 1.767826 | 0.140226 | 12.60693 | 1.93E-36 | 7.15E-35 |
| MCM4 | 2051.802 | 1.741721 | 0.138238 | 12.59944 | 2.13E-36 | 7.81E-35 |
| PHLDA1 | 4413.772 | -2.36343 | 0.188205 | -12.5578 | 3.60E-36 | 1.31E-34 |
| ABCC5 | 693.3374 | 1.200974 | 0.095637 | 12.55763 | 3.61E-36 | 1.31E-34 |
| KPNA2 | 2497.716 | 1.539451 | 0.122741 | 12.54223 | 4.38E-36 | 1.58E-34 |
| SFN | 1403.505 | 3.96855 | 0.317219 | 12.51043 | 6.55E-36 | 2.34E-34 |
| CCDC34 | 493.6207 | 1.784635 | 0.142721 | 12.50437 | 7.07E-36 | 2.51E-34 |
| FOS | 5828.262 | -3.00284 | 0.240504 | -12.4856 | 8.95E-36 | 3.16E-34 |
| COA6 | 1488.193 | 1.389509 | 0.111934 | 12.41363 | 2.20E-35 | 7.73E-34 |
| ETS2 | 9590.913 | -1.57359 | 0.127272 | -12.364 | 4.09E-35 | 1.42E-33 |
| ACLY | 4209.483 | 1.159447 | 0.093907 | 12.34677 | 5.07E-35 | 1.74E-33 |
| CKS1B | 1087.949 | 1.360148 | 0.110383 | 12.32209 | 6.89E-35 | 2.35E-33 |
| COL4A1 | 9175.278 | 1.899587 | 0.154272 | 12.31321 | 7.69E-35 | 2.61E-33 |
| ADAM15 | 3693.548 | 1.441802 | 0.117135 | 12.30893 | 8.11E-35 | 2.74E-33 |
| STIP1 | 5031.394 | 1.036381 | 0.084421 | 12.27635 | 1.21E-34 | 4.07E-33 |
| PLSCR4 | 1013.433 | -1.81364 | 0.147908 | -12.2619 | 1.45E-34 | 4.83E-33 |
| SNRPE | 2500.852 | 1.304888 | 0.106508 | 12.2516 | 1.65E-34 | 5.46E-33 |
| ENAH | 3213.1 | 1.677524 | 0.137097 | 12.23608 | 1.99E-34 | 6.57E-33 |
| CNIH4 | 1624.237 | 1.208971 | 0.098891 | 12.22526 | 2.28E-34 | 7.46E-33 |
| NDST3 | 12.63976 | -4.23495 | 0.346512 | -12.2217 | 2.38E-34 | 7.75E-33 |
| SSR2 | 12136.12 | 1.322989 | 0.108299 | 12.21608 | 2.55E-34 | 8.25E-33 |
| AKR1C3 | 16195.6 | 1.713188 | 0.141095 | 12.14206 | 6.32E-34 | 2.02E-32 |
| DTYMK | 1174.105 | 1.434649 | 0.118323 | 12.12487 | 7.80E-34 | 2.48E-32 |
| MCL1 | 16142.66 | -1.12617 | 0.092912 | -12.1209 | 8.19E-34 | 2.58E-32 |
| KRTCAP2 | 1412.744 | 1.292734 | 0.106662 | 12.11994 | 8.28E-34 | 2.60E-32 |
| ATAD2 | 2072.847 | 1.817111 | 0.15033 | 12.0875 | 1.23E-33 | 3.84E-32 |
| PLOD3 | 5837.39 | 1.147731 | 0.095309 | 12.04217 | 2.13E-33 | 6.58E-32 |
| CDCA4 | 285.9536 | 1.526401 | 0.126867 | 12.03154 | 2.43E-33 | 7.44E-32 |
| RTKN2 | 72.47887 | 2.966838 | 0.24699 | 12.01195 | 3.08E-33 | 9.36E-32 |
| GPAA1 | 10414.05 | 1.533946 | 0.127705 | 12.01164 | 3.09E-33 | 9.36E-32 |
| GPSM2 | 220.6076 | 1.711016 | 0.14275 | 11.98606 | 4.20E-33 | 1.27E-31 |
| SPP1 | 29533.51 | 4.401887 | 0.367589 | 11.97503 | 4.80E-33 | 1.44E-31 |
| MCM5 | 2547.265 | 1.517598 | 0.126738 | 11.97429 | 4.85E-33 | 1.45E-31 |
| MCM7 | 3781.338 | 1.603669 | 0.133967 | 11.97059 | 5.07E-33 | 1.50E-31 |
| CACYBP | 2422.592 | 1.16809 | 0.097709 | 11.95478 | 6.13E-33 | 1.81E-31 |
| LIG1 | 1320.275 | 1.253827 | 0.105054 | 11.93512 | 7.76E-33 | 2.28E-31 |
| DNASE1L3 | 1312.148 | -2.78588 | 0.23378 | -11.9167 | 9.69E-33 | 2.83E-31 |
| TRIM59 | 93.63841 | 2.057419 | 0.17278 | 11.90775 | 1.08E-32 | 3.13E-31 |
| HSP90AB1 | 44901.13 | 1.076799 | 0.090469 | 11.9024 | 1.15E-32 | 3.32E-31 |
| DUSP6 | 3571.597 | -1.62647 | 0.137037 | -11.8688 | 1.72E-32 | 4.91E-31 |
| SNRPB | 6312.877 | 1.276518 | 0.107632 | 11.86003 | 1.91E-32 | 5.43E-31 |
| IL1RAP | 1842.494 | -1.95893 | 0.165396 | -11.8439 | 2.31E-32 | 6.51E-31 |
| ATAD5 | 134.1355 | 1.755015 | 0.148174 | 11.84428 | 2.30E-32 | 6.51E-31 |
| SPRY2 | 623.5664 | -1.41683 | 0.119873 | -11.8194 | 3.10E-32 | 8.67E-31 |
| EPHA2 | 1427.918 | -1.96264 | 0.166131 | -11.8138 | 3.31E-32 | 9.22E-31 |
| SLC38A6 | 391.9012 | 1.299885 | 0.110383 | 11.77613 | 5.18E-32 | 1.43E-30 |
| H2AFZ | 4294.023 | 1.282121 | 0.109202 | 11.74079 | 7.87E-32 | 2.16E-30 |
| CD4 | 2740.031 | -1.86771 | 0.15939 | -11.7179 | 1.03E-31 | 2.81E-30 |
| EGR1 | 7538.107 | -2.50182 | 0.213629 | -11.711 | 1.12E-31 | 3.04E-30 |
| NCAPD2 | 1271.006 | 1.533117 | 0.13132 | 11.67468 | 1.72E-31 | 4.61E-30 |
| SULT1C2 | 769.3139 | 3.704709 | 0.317868 | 11.65486 | 2.17E-31 | 5.79E-30 |
| ACYP1 | 117.9463 | 1.246934 | 0.107328 | 11.61796 | 3.34E-31 | 8.84E-30 |
| CXCL12 | 3237.811 | -2.55921 | 0.222055 | -11.5251 | 9.86E-31 | 2.60E-29 |
| RBM3 | 5409.492 | 1.082368 | 0.094017 | 11.51245 | 1.14E-30 | 2.99E-29 |
| PNRC1 | 4904.775 | -1.31783 | 0.114485 | -11.511 | 1.16E-30 | 3.03E-29 |
| RBP7 | 506.6654 | 2.115014 | 0.18418 | 11.48338 | 1.60E-30 | 4.15E-29 |
| MAFG | 882.776 | 1.398097 | 0.122104 | 11.45009 | 2.35E-30 | 6.04E-29 |
| HMGA1 | 3627.382 | 1.839001 | 0.161052 | 11.41866 | 3.37E-30 | 8.63E-29 |
| GINS2 | 368.3935 | 1.886124 | 0.165648 | 11.38635 | 4.89E-30 | 1.25E-28 |
| HSPB1 | 24564.66 | 1.779134 | 0.156385 | 11.37663 | 5.47E-30 | 1.39E-28 |
| CCL25 | 204.857 | 4.544796 | 0.399643 | 11.37215 | 5.76E-30 | 1.45E-28 |
| LAMC1 | 6332.929 | 1.520765 | 0.134065 | 11.34348 | 7.99E-30 | 2.00E-28 |
| ITGA6 | 2807.688 | 1.523327 | 0.134336 | 11.3397 | 8.34E-30 | 2.08E-28 |
| LYVE1 | 410.0788 | -2.83592 | 0.2501 | -11.3391 | 8.40E-30 | 2.08E-28 |
| SLC50A1 | 3605.2 | 1.347516 | 0.11938 | 11.28759 | 1.51E-29 | 3.73E-28 |
| TYMS | 861.5175 | 1.891709 | 0.167956 | 11.26313 | 2.00E-29 | 4.87E-28 |
| HMGB2 | 1956.8 | 1.483407 | 0.131989 | 11.23885 | 2.63E-29 | 6.39E-28 |
| NEU1 | 5466.921 | 1.269794 | 0.113155 | 11.22173 | 3.19E-29 | 7.69E-28 |
| STXBP6 | 531.8365 | 1.775291 | 0.158217 | 11.22058 | 3.23E-29 | 7.75E-28 |
| COL4A2 | 10385.97 | 1.760904 | 0.157038 | 11.21325 | 3.51E-29 | 8.39E-28 |
| PHF19 | 532.122 | 1.550757 | 0.138729 | 11.17833 | 5.21E-29 | 1.23E-27 |
| NSD2 | 1486.215 | 1.239221 | 0.110944 | 11.1698 | 5.73E-29 | 1.35E-27 |
| PSPH | 1014.316 | 1.584015 | 0.142029 | 11.15272 | 6.94E-29 | 1.62E-27 |
| PLPP3 | 5302.683 | -1.55271 | 0.13972 | -11.113 | 1.08E-28 | 2.50E-27 |
| SAC3D1 | 717.2153 | 1.402177 | 0.126281 | 11.10364 | 1.20E-28 | 2.77E-27 |
| PSMD4 | 11718.5 | 1.198412 | 0.108042 | 11.09205 | 1.37E-28 | 3.14E-27 |
| H2AFX | 1199.806 | 1.568331 | 0.141408 | 11.0908 | 1.39E-28 | 3.17E-27 |
| BCO2 | 536.6579 | -2.98822 | 0.269462 | -11.0896 | 1.41E-28 | 3.20E-27 |
| PEG10 | 8285.561 | 4.728665 | 0.427612 | 11.05831 | 2.00E-28 | 4.50E-27 |
| FRMD4B | 936.3317 | -1.24625 | 0.112714 | -11.0567 | 2.03E-28 | 4.56E-27 |
| PCNA | 2913.524 | 1.172732 | 0.106352 | 11.02693 | 2.83E-28 | 6.32E-27 |
| NAAA | 1021.357 | -1.53247 | 0.138989 | -11.0258 | 2.87E-28 | 6.37E-27 |
| SLC38A2 | 11551.22 | -1.52505 | 0.13882 | -10.9859 | 4.47E-28 | 9.85E-27 |
| ERLIN1 | 3437.73 | -1.17754 | 0.107415 | -10.9625 | 5.79E-28 | 1.27E-26 |
| SIGLEC7 | 69.06939 | -1.94284 | 0.177637 | -10.9372 | 7.66E-28 | 1.67E-26 |
| GHR | 3588.926 | -2.34495 | 0.214457 | -10.9344 | 7.90E-28 | 1.71E-26 |
| UPF3B | 447.021 | 1.002242 | 0.091803 | 10.91729 | 9.53E-28 | 2.05E-26 |
| CD302 | 1393.909 | -1.58257 | 0.145331 | -10.8894 | 1.29E-27 | 2.76E-26 |
| S100A12 | 11.30607 | -2.85757 | 0.262557 | -10.8836 | 1.38E-27 | 2.93E-26 |
| ARID3A | 799.6935 | 2.649716 | 0.244138 | 10.85337 | 1.92E-27 | 4.05E-26 |
| CEP152 | 114.7906 | 1.488347 | 0.137179 | 10.84969 | 2.00E-27 | 4.20E-26 |
| LMNB2 | 1177.733 | 1.443554 | 0.133104 | 10.84528 | 2.10E-27 | 4.39E-26 |
| POLR2K | 2273.3 | 1.037399 | 0.095888 | 10.81881 | 2.80E-27 | 5.80E-26 |
| SIGLEC11 | 46.60086 | -2.23403 | 0.207492 | -10.7668 | 4.94E-27 | 1.01E-25 |
| SEZ6L2 | 1584.96 | 3.559486 | 0.330774 | 10.76109 | 5.25E-27 | 1.07E-25 |
| MPC1 | 4121.135 | -1.43586 | 0.133541 | -10.7522 | 5.79E-27 | 1.18E-25 |
| FOXO1 | 1547.981 | -1.60874 | 0.150116 | -10.7167 | 8.50E-27 | 1.71E-25 |
| MAP2K3 | 2945.31 | -1.13378 | 0.105938 | -10.7023 | 9.93E-27 | 1.99E-25 |
| ITGB1BP1 | 1164.548 | 1.000465 | 0.093506 | 10.69951 | 1.02E-26 | 2.05E-25 |
| SLC39A14 | 17369.9 | -1.52802 | 0.143329 | -10.661 | 1.55E-26 | 3.08E-25 |
| SAMD1 | 1504.249 | 1.079513 | 0.10131 | 10.6555 | 1.64E-26 | 3.25E-25 |
| LRRC4 | 39.00305 | -1.76739 | 0.16602 | -10.6457 | 1.83E-26 | 3.60E-25 |
| PLK3 | 388.2574 | -1.4768 | 0.139283 | -10.6029 | 2.89E-26 | 5.67E-25 |
| MCAM | 2397.12 | 1.491758 | 0.140894 | 10.58779 | 3.40E-26 | 6.64E-25 |
| C1R | 64330.83 | -1.66875 | 0.157786 | -10.576 | 3.85E-26 | 7.47E-25 |
| ATP6V1C1 | 3263.308 | 1.119791 | 0.105919 | 10.57217 | 4.01E-26 | 7.76E-25 |
| FOSB | 1922.987 | -3.13353 | 0.296879 | -10.5549 | 4.82E-26 | 9.30E-25 |
| CLN3 | 1612.801 | 1.219326 | 0.115571 | 10.55041 | 5.06E-26 | 9.72E-25 |
| PAFAH1B3 | 816.002 | 2.121288 | 0.201332 | 10.53628 | 5.88E-26 | 1.13E-24 |
| TIMD4 | 74.49698 | -3.33402 | 0.316622 | -10.53 | 6.29E-26 | 1.20E-24 |
| RASGEF1B | 1022.659 | -1.72095 | 0.164014 | -10.4927 | 9.33E-26 | 1.77E-24 |
| SERTAD1 | 848.383 | -1.43553 | 0.137191 | -10.4637 | 1.27E-25 | 2.38E-24 |
| PPP1R35 | 767.5678 | 1.205942 | 0.115896 | 10.40539 | 2.34E-25 | 4.36E-24 |
| GLA | 1008.46 | 1.29038 | 0.124121 | 10.39617 | 2.58E-25 | 4.79E-24 |
| DNMT1 | 1689.349 | 1.289009 | 0.124035 | 10.3923 | 2.69E-25 | 4.97E-24 |
| NCAPG2 | 538.3105 | 1.354375 | 0.130343 | 10.39084 | 2.73E-25 | 5.03E-24 |
| ALDH2 | 43183.38 | -1.57219 | 0.151324 | -10.3895 | 2.77E-25 | 5.06E-24 |
| AGO2 | 1246.123 | 1.142218 | 0.110497 | 10.33706 | 4.79E-25 | 8.68E-24 |
| MS4A6A | 1159.964 | -1.7175 | 0.166831 | -10.2949 | 7.43E-25 | 1.33E-23 |
| ACADSB | 12310.99 | -1.65501 | 0.1611 | -10.2732 | 9.31E-25 | 1.66E-23 |
| N4BP2L1 | 1247.742 | -1.3368 | 0.130301 | -10.2593 | 1.07E-24 | 1.89E-23 |
| PFDN6 | 2619.518 | 1.16278 | 0.113507 | 10.24408 | 1.26E-24 | 2.20E-23 |
| STK39 | 500.7312 | 2.116246 | 0.206798 | 10.23338 | 1.41E-24 | 2.45E-23 |
| NRM | 594.4307 | 1.604525 | 0.156801 | 10.23288 | 1.41E-24 | 2.45E-23 |
| ID2 | 7959.536 | -1.33998 | 0.130958 | -10.2322 | 1.42E-24 | 2.46E-23 |
| CKS2 | 771.1765 | 1.479744 | 0.145172 | 10.19307 | 2.13E-24 | 3.67E-23 |
| CKAP2 | 496.854 | 1.337691 | 0.131732 | 10.15467 | 3.16E-24 | 5.43E-23 |
| LILRB5 | 349.6481 | -1.81036 | 0.178475 | -10.1435 | 3.54E-24 | 6.05E-23 |
| NDRG2 | 9868.566 | -1.39178 | 0.137325 | -10.1349 | 3.87E-24 | 6.59E-23 |
| IER2 | 3325.485 | -1.22488 | 0.120963 | -10.1261 | 4.23E-24 | 7.19E-23 |
| CTSA | 14115.37 | 1.042111 | 0.103245 | 10.09356 | 5.90E-24 | 9.92E-23 |
| EEF1A2 | 3991.397 | 4.055557 | 0.40201 | 10.08821 | 6.23E-24 | 1.04E-22 |
| LDLR | 5061.255 | -1.40357 | 0.139469 | -10.0637 | 7.99E-24 | 1.33E-22 |
| LARP1B | 1300.003 | -1.00629 | 0.100057 | -10.0572 | 8.54E-24 | 1.42E-22 |
| IGFBP3 | 10511.59 | -1.87046 | 0.186094 | -10.0511 | 9.08E-24 | 1.50E-22 |
| AKR1B10 | 25537.1 | 3.718962 | 0.370757 | 10.03073 | 1.12E-23 | 1.84E-22 |
| NCOR1 | 2759.291 | -1.02056 | 0.101825 | -10.0227 | 1.21E-23 | 1.99E-22 |
| GADD45B | 7412.77 | -1.82108 | 0.182214 | -9.99417 | 1.62E-23 | 2.64E-22 |
| LSM4 | 4594.339 | 1.101864 | 0.110523 | 9.969593 | 2.07E-23 | 3.37E-22 |
| AC007906.2 | 24.09614 | -2.38188 | 0.239095 | -9.96206 | 2.23E-23 | 3.62E-22 |
| ACAA1 | 10748.22 | -1.4158 | 0.142165 | -9.95885 | 2.31E-23 | 3.72E-22 |
| FPR2 | 15.90899 | -2.73018 | 0.27465 | -9.94058 | 2.77E-23 | 4.44E-22 |
| ITGA9 | 649.1792 | -1.72138 | 0.173192 | -9.93913 | 2.81E-23 | 4.49E-22 |
| CD163 | 1616.616 | -1.87718 | 0.189111 | -9.92632 | 3.20E-23 | 5.08E-22 |
| GNE | 4137.099 | -1.45877 | 0.147122 | -9.91539 | 3.57E-23 | 5.63E-22 |
| SOCS3 | 2265.593 | -2.18723 | 0.220871 | -9.90273 | 4.05E-23 | 6.36E-22 |
| PLXNC1 | 728.4885 | 1.934427 | 0.195651 | 9.887126 | 4.73E-23 | 7.41E-22 |
| SERPINB9 | 601.4809 | -1.47183 | 0.149504 | -9.84472 | 7.22E-23 | 1.12E-21 |
| TNFRSF25 | 281.53 | 1.648314 | 0.167828 | 9.821424 | 9.10E-23 | 1.41E-21 |
| HIST1H3B | 14.39449 | 3.391458 | 0.346389 | 9.79089 | 1.23E-22 | 1.90E-21 |
| P2RY13 | 104.7553 | -1.8373 | 0.187783 | -9.78417 | 1.32E-22 | 2.02E-21 |
| JPT1 | 2183.325 | 1.428531 | 0.146159 | 9.773826 | 1.46E-22 | 2.22E-21 |
| GLMP | 4450.359 | 1.084013 | 0.11094 | 9.771125 | 1.50E-22 | 2.27E-21 |
| HPS5 | 1604.247 | -1.41622 | 0.145325 | -9.74524 | 1.93E-22 | 2.90E-21 |
| FAM50A | 3897.293 | 1.198894 | 0.123068 | 9.74173 | 2.00E-22 | 2.98E-21 |
| NFIL3 | 2362.059 | -1.3076 | 0.134368 | -9.7315 | 2.21E-22 | 3.28E-21 |
| FABP5 | 286.0182 | 1.806999 | 0.185724 | 9.729468 | 2.26E-22 | 3.34E-21 |
| SPAG5 | 1264.422 | 1.47463 | 0.151668 | 9.722743 | 2.41E-22 | 3.56E-21 |
| PHPT1 | 3529.504 | 1.351796 | 0.139146 | 9.714943 | 2.60E-22 | 3.83E-21 |
| CHKA | 1883.763 | 1.242778 | 0.127944 | 9.713435 | 2.64E-22 | 3.88E-21 |
| ALPK3 | 776.4037 | 2.18007 | 0.224811 | 9.697339 | 3.09E-22 | 4.53E-21 |
| JUN | 7780.33 | -1.43035 | 0.147812 | -9.67686 | 3.78E-22 | 5.50E-21 |
| RPP21 | 315.4148 | 1.030624 | 0.106531 | 9.674379 | 3.87E-22 | 5.62E-21 |
| ACAT1 | 15233.48 | -1.29033 | 0.133873 | -9.63847 | 5.50E-22 | 7.86E-21 |
| JUNB | 5308.584 | -1.43474 | 0.148931 | -9.63358 | 5.77E-22 | 8.22E-21 |
| PGP | 1023.019 | 1.284283 | 0.133339 | 9.631728 | 5.87E-22 | 8.34E-21 |
| TKT | 13463.14 | 1.608763 | 0.167034 | 9.631325 | 5.90E-22 | 8.36E-21 |
| MRPS21 | 3598.242 | 1.077848 | 0.111942 | 9.628593 | 6.06E-22 | 8.56E-21 |
| GBP2 | 3929.452 | 1.48764 | 0.154636 | 9.620248 | 6.57E-22 | 9.26E-21 |
| RFC3 | 369.5405 | 1.167614 | 0.12188 | 9.580005 | 9.70E-22 | 1.35E-20 |
| TMCO3 | 1776.505 | 1.371822 | 0.143201 | 9.579664 | 9.74E-22 | 1.35E-20 |
| ACAA2 | 16070.44 | -1.47491 | 0.15416 | -9.56735 | 1.10E-21 | 1.52E-20 |
| SLC39A10 | 446.2454 | 1.38575 | 0.144996 | 9.557191 | 1.21E-21 | 1.67E-20 |
| HIST1H3D | 30.65076 | 2.368632 | 0.247869 | 9.556 | 1.22E-21 | 1.69E-20 |
| IVD | 8273.647 | -1.10556 | 0.115713 | -9.55429 | 1.24E-21 | 1.71E-20 |
| RNF125 | 597.3057 | -1.76522 | 0.185844 | -9.49836 | 2.13E-21 | 2.88E-20 |
| BRCA2 | 137.8224 | 1.42387 | 0.15006 | 9.488647 | 2.34E-21 | 3.14E-20 |
| BSG | 20270.03 | 1.145005 | 0.120702 | 9.486219 | 2.40E-21 | 3.20E-20 |
| SYNE1 | 1161.892 | -1.19938 | 0.12651 | -9.48058 | 2.53E-21 | 3.36E-20 |
| MBNL2 | 2661.496 | -1.24882 | 0.131735 | -9.47976 | 2.55E-21 | 3.38E-20 |
| DBF4 | 250.8732 | 1.029312 | 0.108725 | 9.4671 | 2.88E-21 | 3.81E-20 |
| ATF3 | 2184.203 | -1.6233 | 0.172012 | -9.43712 | 3.83E-21 | 5.03E-20 |
| ANXA2 | 14208.53 | 1.28849 | 0.136666 | 9.428058 | 4.18E-21 | 5.46E-20 |
| BAX | 2380.951 | 1.013835 | 0.107829 | 9.402281 | 5.34E-21 | 6.91E-20 |
| NME1 | 2720.768 | 1.300327 | 0.138323 | 9.400653 | 5.42E-21 | 7.00E-20 |
| TESC | 1261.405 | 2.897774 | 0.309251 | 9.370304 | 7.23E-21 | 9.26E-20 |
| BARD1 | 165.5306 | 1.388502 | 0.148228 | 9.36731 | 7.44E-21 | 9.50E-20 |
| ITGA2 | 394.8292 | 2.079747 | 0.222059 | 9.365747 | 7.55E-21 | 9.62E-20 |
| RIPK4 | 1164.644 | -1.2526 | 0.134086 | -9.34175 | 9.48E-21 | 1.19E-19 |
| CYP2C8 | 21099.23 | -2.5689 | 0.275259 | -9.33269 | 1.03E-20 | 1.29E-19 |
| NR4A1 | 1551.53 | -2.03099 | 0.217771 | -9.32625 | 1.10E-20 | 1.37E-19 |
| SCIMP | 127.0608 | -1.57077 | 0.168807 | -9.30509 | 1.34E-20 | 1.66E-19 |
| DLK1 | 2391.356 | 6.277688 | 0.675399 | 9.294789 | 1.47E-20 | 1.82E-19 |
| RAP2A | 1484.645 | 1.142578 | 0.122995 | 9.289594 | 1.55E-20 | 1.90E-19 |
| KLF10 | 2669.621 | -1.32889 | 0.143076 | -9.288 | 1.57E-20 | 1.92E-19 |
| UQCC2 | 1962.148 | 1.119243 | 0.120528 | 9.2862 | 1.60E-20 | 1.95E-19 |
| CAT | 18885.31 | -1.30842 | 0.14115 | -9.26976 | 1.87E-20 | 2.27E-19 |
| ACSM3 | 1554.877 | -1.93474 | 0.208938 | -9.25987 | 2.05E-20 | 2.48E-19 |
| MAT1A | 48771.3 | -1.70471 | 0.18418 | -9.25566 | 2.13E-20 | 2.57E-19 |
| EGR3 | 77.23571 | -2.03053 | 0.219637 | -9.24496 | 2.35E-20 | 2.84E-19 |
| ALDH6A1 | 11780.4 | -1.63969 | 0.177646 | -9.2301 | 2.70E-20 | 3.24E-19 |
| ACSL1 | 30269.34 | -1.72627 | 0.187096 | -9.22663 | 2.79E-20 | 3.34E-19 |
| IRX3 | 228.1196 | 2.645597 | 0.286897 | 9.221429 | 2.93E-20 | 3.49E-19 |
| IRF8 | 849.0596 | -1.46354 | 0.158803 | -9.21606 | 3.08E-20 | 3.67E-19 |
| NOP56 | 3060.949 | 1.020455 | 0.110845 | 9.206182 | 3.38E-20 | 4.01E-19 |
| SOWAHC | 1929.706 | -1.0462 | 0.113725 | -9.19939 | 3.60E-20 | 4.26E-19 |
| CKAP4 | 5198.11 | 1.098752 | 0.119521 | 9.192977 | 3.82E-20 | 4.50E-19 |
| MASP1 | 6030.804 | -1.50157 | 0.163587 | -9.17904 | 4.35E-20 | 5.12E-19 |
| SQLE | 4267.46 | 1.741465 | 0.189829 | 9.173849 | 4.56E-20 | 5.33E-19 |
| SDC4 | 20372.64 | -1.14909 | 0.12562 | -9.14729 | 5.84E-20 | 6.76E-19 |
| KLRF1 | 18.53996 | -1.69708 | 0.185595 | -9.14403 | 6.02E-20 | 6.96E-19 |
| CYHR1 | 3136.189 | 1.015762 | 0.111175 | 9.13657 | 6.45E-20 | 7.44E-19 |
| PLIN2 | 17998.92 | -1.5606 | 0.170846 | -9.13454 | 6.57E-20 | 7.56E-19 |
| PUF60 | 9355.079 | 1.031535 | 0.113081 | 9.122076 | 7.37E-20 | 8.47E-19 |
| PBLD | 5256.448 | -1.64825 | 0.180703 | -9.12128 | 7.42E-20 | 8.51E-19 |
| STXBP4 | 307.55 | 1.171289 | 0.128487 | 9.115991 | 7.80E-20 | 8.90E-19 |
| S100A10 | 6251.597 | 1.37742 | 0.151425 | 9.096404 | 9.34E-20 | 1.06E-18 |
| LAPTM4B | 5203.615 | 1.673892 | 0.184219 | 9.086417 | 1.02E-19 | 1.16E-18 |
| LAGE3 | 1102.374 | 1.251212 | 0.138366 | 9.042742 | 1.53E-19 | 1.73E-18 |
| CXCR2 | 19.21197 | -2.25098 | 0.249216 | -9.03225 | 1.68E-19 | 1.90E-18 |
| CAMK4 | 68.08247 | -1.61977 | 0.179474 | -9.02513 | 1.79E-19 | 2.02E-18 |
| PROK2 | 3.623519 | -2.77733 | 0.308046 | -9.01594 | 1.95E-19 | 2.19E-18 |
| TTF2 | 283.3719 | 1.111902 | 0.123407 | 9.010074 | 2.06E-19 | 2.30E-18 |
| SERPINE1 | 8821.431 | -2.10816 | 0.234252 | -8.99954 | 2.27E-19 | 2.52E-18 |
| KLF13 | 2774.433 | 1.172911 | 0.130465 | 8.990228 | 2.47E-19 | 2.74E-18 |
| PLCB1 | 674.1341 | 1.631956 | 0.18181 | 8.976183 | 2.80E-19 | 3.07E-18 |
| FAM13A | 1075.773 | -1.79312 | 0.200348 | -8.95001 | 3.55E-19 | 3.85E-18 |
| PTGS2 | 68.62523 | -2.31575 | 0.259021 | -8.94042 | 3.88E-19 | 4.19E-18 |
| RHOB | 27325.29 | -1.36105 | 0.152363 | -8.93299 | 4.15E-19 | 4.47E-18 |
| PKM | 7767.077 | 1.950037 | 0.218718 | 8.915774 | 4.84E-19 | 5.19E-18 |
| DUSP1 | 12252.13 | -1.56718 | 0.175986 | -8.90513 | 5.33E-19 | 5.68E-18 |
| HMGCL | 8064.659 | -1.17513 | 0.131958 | -8.90535 | 5.32E-19 | 5.68E-18 |
| TUBB4A | 454.1727 | 3.313852 | 0.37231 | 8.900781 | 5.55E-19 | 5.89E-18 |
| NSUN6 | 1139.233 | -1.01627 | 0.1142 | -8.89909 | 5.63E-19 | 5.96E-18 |
| CYP4A11 | 20240.99 | -2.13102 | 0.239578 | -8.89489 | 5.85E-19 | 6.15E-18 |
| CKLF | 284.1362 | 1.136415 | 0.127968 | 8.880462 | 6.66E-19 | 6.95E-18 |
| NR4A3 | 222.4129 | -2.09637 | 0.236457 | -8.86577 | 7.60E-19 | 7.90E-18 |
| KCNJ5 | 215.6617 | 2.010693 | 0.226975 | 8.858658 | 8.10E-19 | 8.38E-18 |
| TLR4 | 390.3661 | -1.36635 | 0.154463 | -8.8458 | 9.09E-19 | 9.35E-18 |
| DDX39B | 1955.943 | 1.124031 | 0.12722 | 8.835335 | 9.98E-19 | 1.02E-17 |
| DLC1 | 1651.075 | -1.14218 | 0.129318 | -8.83233 | 1.03E-18 | 1.05E-17 |
| ALB | 3048748 | -1.7303 | 0.195982 | -8.82887 | 1.06E-18 | 1.08E-17 |
| ESCO2 | 98.92801 | 1.760668 | 0.19984 | 8.810411 | 1.25E-18 | 1.27E-17 |
| IGF2BP2 | 746.9831 | 2.415992 | 0.274268 | 8.808862 | 1.26E-18 | 1.28E-17 |
| KLF6 | 6529.227 | -1.22513 | 0.139098 | -8.80768 | 1.28E-18 | 1.29E-17 |
| ALDH8A1 | 4844.318 | -1.8049 | 0.20519 | -8.79624 | 1.41E-18 | 1.42E-17 |
| MANF | 4427.934 | 1.120963 | 0.127536 | 8.789352 | 1.50E-18 | 1.51E-17 |
| AQP3 | 3095.501 | -1.44086 | 0.163941 | -8.7889 | 1.51E-18 | 1.51E-17 |
| RSRP1 | 1056.769 | 1.026646 | 0.116868 | 8.784661 | 1.57E-18 | 1.56E-17 |
| TOMM40 | 3107.172 | 1.008071 | 0.114755 | 8.784574 | 1.57E-18 | 1.56E-17 |
| PPP1R3B | 4589.945 | -1.555 | 0.177059 | -8.7824 | 1.60E-18 | 1.59E-17 |
| CYP2B6 | 8451.484 | -2.52094 | 0.287669 | -8.76333 | 1.90E-18 | 1.87E-17 |
| NAA10 | 1798.33 | 1.078613 | 0.123136 | 8.759561 | 1.96E-18 | 1.93E-17 |
| TXNRD1 | 7764.126 | 1.501788 | 0.171528 | 8.755337 | 2.03E-18 | 1.99E-17 |
| PKN1 | 4968 | 1.164348 | 0.133211 | 8.740655 | 2.32E-18 | 2.25E-17 |
| CCSAP | 365.8353 | 1.141858 | 0.130857 | 8.725971 | 2.64E-18 | 2.55E-17 |
| SERPINH1 | 4458.006 | 1.228181 | 0.141003 | 8.710324 | 3.03E-18 | 2.90E-17 |
| CD160 | 29.37796 | -1.55812 | 0.17895 | -8.70703 | 3.12E-18 | 2.98E-17 |
| RBMS3 | 194.2555 | -1.41107 | 0.162339 | -8.6921 | 3.56E-18 | 3.39E-17 |
| TNFRSF18 | 76.67414 | 2.066731 | 0.23785 | 8.689218 | 3.65E-18 | 3.46E-17 |
| LPCAT1 | 1835.133 | 1.536543 | 0.177222 | 8.670155 | 4.32E-18 | 4.08E-17 |
| CCDC14 | 758.6423 | 1.044889 | 0.120885 | 8.643663 | 5.44E-18 | 5.08E-17 |
| PROS1 | 9213.247 | -1.11475 | 0.129316 | -8.62038 | 6.67E-18 | 6.21E-17 |
| BLVRA | 913.9982 | 1.372127 | 0.159259 | 8.615716 | 6.95E-18 | 6.46E-17 |
| LMNB1 | 1024.705 | 1.341928 | 0.156194 | 8.591431 | 8.59E-18 | 7.91E-17 |
| BHLHE40 | 7419.082 | -1.26848 | 0.147986 | -8.57162 | 1.02E-17 | 9.34E-17 |
| BDH2 | 1220.281 | -1.1908 | 0.138971 | -8.56873 | 1.05E-17 | 9.54E-17 |
| ECHS1 | 28056.74 | -1.09432 | 0.128073 | -8.54451 | 1.29E-17 | 1.18E-16 |
| MYBL1 | 180.3609 | 1.32596 | 0.155205 | 8.543281 | 1.30E-17 | 1.19E-16 |
| JDP2 | 526.8497 | -1.07375 | 0.125943 | -8.52568 | 1.52E-17 | 1.38E-16 |
| SMC2 | 570.2347 | 1.02288 | 0.120117 | 8.515719 | 1.66E-17 | 1.49E-16 |
| FBP1 | 15992.74 | -1.91374 | 0.225218 | -8.49727 | 1.94E-17 | 1.74E-16 |
| PDZK1IP1 | 2661.751 | 3.071194 | 0.361536 | 8.494856 | 1.98E-17 | 1.77E-16 |
| VMO1 | 155.1187 | -1.28747 | 0.151681 | -8.48801 | 2.10E-17 | 1.88E-16 |
| CD5L | 1006.204 | -2.92116 | 0.344645 | -8.47585 | 2.33E-17 | 2.08E-16 |
| SLC51B | 396.6773 | 2.446425 | 0.289046 | 8.463776 | 2.59E-17 | 2.29E-16 |
| VSIG4 | 490.4625 | -1.7914 | 0.211845 | -8.45617 | 2.76E-17 | 2.43E-16 |
| ACSL4 | 12331.06 | 2.277916 | 0.269792 | 8.443241 | 3.09E-17 | 2.71E-16 |
| PLP2 | 1703.911 | 1.641022 | 0.194716 | 8.427767 | 3.52E-17 | 3.07E-16 |
| EPHX2 | 6390.012 | -1.40922 | 0.167246 | -8.42603 | 3.58E-17 | 3.11E-16 |
| CFI | 19919.07 | -1.26104 | 0.150148 | -8.39861 | 4.52E-17 | 3.91E-16 |
| MYO1B | 11595.18 | -1.04401 | 0.124338 | -8.39658 | 4.60E-17 | 3.97E-16 |
| ASS1 | 49576.99 | -1.65488 | 0.197854 | -8.36414 | 6.06E-17 | 5.21E-16 |
| SLC41A2 | 2523.824 | -1.25818 | 0.151162 | -8.32339 | 8.55E-17 | 7.30E-16 |
| C1S | 101167.9 | -1.23309 | 0.148188 | -8.32112 | 8.71E-17 | 7.43E-16 |
| BCAT1 | 252.5908 | 1.51663 | 0.182502 | 8.310198 | 9.55E-17 | 8.11E-16 |
| GCH1 | 2080.764 | -1.41643 | 0.170485 | -8.30826 | 9.71E-17 | 8.23E-16 |
| PABPC1 | 39204.38 | 1.079283 | 0.129921 | 8.307249 | 9.79E-17 | 8.29E-16 |
| CDC25B | 2342.65 | 1.134615 | 0.136721 | 8.298785 | 1.05E-16 | 8.87E-16 |
| PPP1R16A | 4699.561 | 1.260658 | 0.152072 | 8.28987 | 1.13E-16 | 9.53E-16 |
| RPL22L1 | 1942.051 | 1.554635 | 0.1879 | 8.273726 | 1.30E-16 | 1.09E-15 |
| MAN1A1 | 7644.546 | -1.06729 | 0.129105 | -8.26686 | 1.38E-16 | 1.14E-15 |
| MT2A | 37072.96 | -2.33592 | 0.282652 | -8.26429 | 1.41E-16 | 1.17E-15 |
| KIF20B | 245.5891 | 1.1072 | 0.13403 | 8.260857 | 1.45E-16 | 1.20E-15 |
| WEE1 | 1605.087 | -1.05014 | 0.127536 | -8.23409 | 1.81E-16 | 1.48E-15 |
| MS4A7 | 743.8515 | -1.37603 | 0.167848 | -8.19804 | 2.44E-16 | 1.97E-15 |
| SNRPD2 | 5173.884 | 1.050244 | 0.128231 | 8.190236 | 2.61E-16 | 2.10E-15 |
| TUBA1B | 3957.415 | 1.072733 | 0.13099 | 8.189445 | 2.62E-16 | 2.11E-15 |
| PIM1 | 2044.211 | -1.24791 | 0.152656 | -8.17468 | 2.97E-16 | 2.38E-15 |
| C8A | 13000.83 | -1.7233 | 0.211817 | -8.13579 | 4.09E-16 | 3.27E-15 |
| ABAT | 15492.54 | -1.49865 | 0.18437 | -8.12849 | 4.35E-16 | 3.47E-15 |
| PDGFB | 674.0768 | 1.038632 | 0.127805 | 8.126715 | 4.41E-16 | 3.51E-15 |
| ORM1 | 146055.2 | -1.73286 | 0.213533 | -8.11516 | 4.85E-16 | 3.85E-15 |
| CLIC1 | 8123.18 | 1.056361 | 0.130402 | 8.100833 | 5.46E-16 | 4.30E-15 |
| HIST1H1B | 3.427992 | 3.209784 | 0.396236 | 8.100679 | 5.47E-16 | 4.30E-15 |
| FAM3B | 868.6474 | 3.109097 | 0.383884 | 8.099057 | 5.54E-16 | 4.35E-15 |
| SLC22A10 | 1382.389 | -2.20323 | 0.272197 | -8.09424 | 5.76E-16 | 4.52E-15 |
| ACADM | 5291.449 | -1.16665 | 0.144397 | -8.07945 | 6.51E-16 | 5.10E-15 |
| AXL | 590.952 | -1.25805 | 0.155834 | -8.07296 | 6.86E-16 | 5.36E-15 |
| PLAC8 | 145.4095 | -2.16513 | 0.268313 | -8.06944 | 7.06E-16 | 5.51E-15 |
| SCP2 | 20895.64 | -1.26198 | 0.156516 | -8.06292 | 7.45E-16 | 5.80E-15 |
| BPIFB2 | 332.4827 | 3.814095 | 0.473454 | 8.055902 | 7.89E-16 | 6.13E-15 |
| TDGF1 | 387.4947 | 2.542762 | 0.316206 | 8.041466 | 8.88E-16 | 6.89E-15 |
| SPAG4 | 276.6834 | 1.53203 | 0.190587 | 8.038494 | 9.09E-16 | 7.04E-15 |
| DSE | 355.3123 | -1.30192 | 0.162463 | -8.01366 | 1.11E-15 | 8.55E-15 |
| C6 | 15456.1 | -1.84489 | 0.230218 | -8.01365 | 1.11E-15 | 8.55E-15 |
| COLEC12 | 154.4441 | 2.222722 | 0.277588 | 8.007278 | 1.17E-15 | 8.99E-15 |
| MMP14 | 3880.899 | 1.467656 | 0.183403 | 8.002359 | 1.22E-15 | 9.35E-15 |
| SMC4 | 778.8868 | 1.381532 | 0.173036 | 7.984057 | 1.42E-15 | 1.08E-14 |
| GAS2L3 | 246.5001 | 1.282261 | 0.160665 | 7.980947 | 1.45E-15 | 1.10E-14 |
| VPS28 | 7916.435 | 1.071681 | 0.134896 | 7.944477 | 1.95E-15 | 1.48E-14 |
| PLGLB2 | 176.8685 | -1.80082 | 0.227225 | -7.92528 | 2.28E-15 | 1.71E-14 |
| ARHGEF2 | 1267.705 | 1.16201 | 0.146834 | 7.913788 | 2.50E-15 | 1.87E-14 |
| MFSD10 | 1149.143 | 1.228662 | 0.155288 | 7.912163 | 2.53E-15 | 1.89E-14 |
| FPR1 | 133.2564 | -1.83426 | 0.231836 | -7.91187 | 2.54E-15 | 1.89E-14 |
| CENPX | 2669.407 | 1.168686 | 0.147787 | 7.907909 | 2.62E-15 | 1.94E-14 |
| NAMPT | 8435.367 | -1.38666 | 0.175391 | -7.90609 | 2.66E-15 | 1.97E-14 |
| CCR1 | 245.6658 | -1.39615 | 0.17672 | -7.90034 | 2.78E-15 | 2.06E-14 |
| MYOM2 | 102.2635 | -1.93044 | 0.244853 | -7.88408 | 3.17E-15 | 2.33E-14 |
| SELENOM | 1794.145 | 1.900081 | 0.241084 | 7.881392 | 3.24E-15 | 2.38E-14 |
| SPON2 | 8531.824 | 1.175166 | 0.149174 | 7.877821 | 3.33E-15 | 2.44E-14 |
| CAPS | 551.9875 | 1.094583 | 0.139255 | 7.860306 | 3.83E-15 | 2.80E-14 |
| CCL3 | 204.1298 | -1.50079 | 0.191044 | -7.85576 | 3.97E-15 | 2.90E-14 |
| APOL6 | 5313.443 | -1.00718 | 0.128622 | -7.83057 | 4.86E-15 | 3.52E-14 |
| CLEC12A | 44.39008 | -1.58366 | 0.202298 | -7.82832 | 4.94E-15 | 3.58E-14 |
| AGTR1 | 1914.011 | -1.34269 | 0.171677 | -7.82099 | 5.24E-15 | 3.79E-14 |
| CKB | 2697.694 | 2.208404 | 0.282739 | 7.810765 | 5.68E-15 | 4.09E-14 |
| PON3 | 7484.543 | -1.42258 | 0.182161 | -7.80947 | 5.74E-15 | 4.12E-14 |
| RPL8 | 70264.04 | 1.277842 | 0.163753 | 7.803482 | 6.02E-15 | 4.32E-14 |
| HOMER3 | 551.0691 | 1.395025 | 0.178905 | 7.797586 | 6.31E-15 | 4.50E-14 |
| HIST1H2AL | 2.339622 | 3.016098 | 0.388796 | 7.757528 | 8.66E-15 | 6.12E-14 |
| C8B | 17544.47 | -1.45798 | 0.188036 | -7.75374 | 8.92E-15 | 6.29E-14 |
| SLC7A2 | 10188.65 | -1.60732 | 0.207347 | -7.75184 | 9.06E-15 | 6.37E-14 |
| SERPING1 | 123779.3 | -1.11821 | 0.144489 | -7.73905 | 1.00E-14 | 7.00E-14 |
| CR1 | 54.99658 | -1.9663 | 0.254077 | -7.73901 | 1.00E-14 | 7.00E-14 |
| ARHGAP10 | 319.1437 | -1.36859 | 0.176916 | -7.73585 | 1.03E-14 | 7.17E-14 |
| NR4A2 | 509.5433 | -1.78263 | 0.230992 | -7.71726 | 1.19E-14 | 8.25E-14 |
| SIGLEC9 | 88.83573 | -1.29047 | 0.16747 | -7.70565 | 1.30E-14 | 9.00E-14 |
| FEZ1 | 229.2546 | -1.32618 | 0.17217 | -7.70273 | 1.33E-14 | 9.20E-14 |
| LRG1 | 31437.08 | -1.55616 | 0.202152 | -7.69796 | 1.38E-14 | 9.50E-14 |
| LIMK2 | 1393.549 | 1.107917 | 0.144264 | 7.679779 | 1.59E-14 | 1.08E-13 |
| NTS | 907.6913 | 4.218074 | 0.549917 | 7.670387 | 1.71E-14 | 1.16E-13 |
| LYZ | 12999.97 | 2.275767 | 0.297077 | 7.660519 | 1.85E-14 | 1.25E-13 |
| BEX2 | 333.4888 | 2.695154 | 0.352488 | 7.646094 | 2.07E-14 | 1.39E-13 |
| CD24 | 5664.831 | 2.175881 | 0.284782 | 7.640506 | 2.16E-14 | 1.44E-13 |
| PLG | 70832.83 | -1.59637 | 0.209035 | -7.63686 | 2.23E-14 | 1.48E-13 |
| SLC16A3 | 1071.484 | 1.7927 | 0.23494 | 7.630461 | 2.34E-14 | 1.56E-13 |
| SLC8A1 | 157.1009 | -1.22673 | 0.160881 | -7.62511 | 2.44E-14 | 1.62E-13 |
| HPX | 133717.6 | -1.62868 | 0.213689 | -7.62173 | 2.50E-14 | 1.65E-13 |
| MT1F | 2954.528 | -2.7944 | 0.366715 | -7.62009 | 2.53E-14 | 1.67E-13 |
| PEMT | 3128.818 | -1.21067 | 0.158904 | -7.61888 | 2.56E-14 | 1.69E-13 |
| IL1B | 68.2934 | -1.7081 | 0.224813 | -7.59785 | 3.01E-14 | 1.97E-13 |
| GLUL | 144854.7 | 2.172008 | 0.286016 | 7.594014 | 3.10E-14 | 2.02E-13 |
| SH2D1B | 17.49169 | -1.34317 | 0.177205 | -7.57978 | 3.46E-14 | 2.25E-13 |
| HIST1H2AG | 165.2715 | 2.212295 | 0.291881 | 7.579452 | 3.47E-14 | 2.26E-13 |
| ARL2 | 921.1307 | 1.144491 | 0.151118 | 7.573514 | 3.63E-14 | 2.35E-13 |
| MCTP1 | 333.0515 | 1.667354 | 0.220368 | 7.566212 | 3.84E-14 | 2.48E-13 |
| SPHK1 | 920.4739 | 2.292464 | 0.303399 | 7.555944 | 4.16E-14 | 2.67E-13 |
| TTYH3 | 3315.168 | 1.135624 | 0.150909 | 7.525232 | 5.26E-14 | 3.35E-13 |
| GRHPR | 16658.47 | -1.09053 | 0.144919 | -7.5251 | 5.27E-14 | 3.35E-13 |
| PRKAR2B | 86.27771 | -1.75404 | 0.233157 | -7.52299 | 5.35E-14 | 3.40E-13 |
| MT1X | 13563.13 | -2.41449 | 0.3211 | -7.51942 | 5.50E-14 | 3.48E-13 |
| UGP2 | 14290.6 | -1.01091 | 0.13515 | -7.47986 | 7.44E-14 | 4.68E-13 |
| ATP11C | 1350.701 | -1.16309 | 0.155526 | -7.47842 | 7.52E-14 | 4.72E-13 |
| TREM2 | 233.1343 | 1.694538 | 0.226995 | 7.4651 | 8.32E-14 | 5.20E-13 |
| RPSA | 19373.25 | 1.032644 | 0.138421 | 7.460165 | 8.64E-14 | 5.39E-13 |
| B3GNT5 | 453.4447 | 1.794499 | 0.240559 | 7.459717 | 8.67E-14 | 5.41E-13 |
| HP | 494778.8 | -1.91361 | 0.257087 | -7.44345 | 9.81E-14 | 6.09E-13 |
| RETREG1 | 993.1151 | -2.15594 | 0.289809 | -7.43918 | 1.01E-13 | 6.27E-13 |
| SQSTM1 | 32250.03 | 1.18034 | 0.158904 | 7.427984 | 1.10E-13 | 6.79E-13 |
| SERPINA11 | 9388.498 | -1.8668 | 0.251768 | -7.41479 | 1.22E-13 | 7.48E-13 |
| SPINK1 | 15040.59 | 2.977322 | 0.402026 | 7.405802 | 1.30E-13 | 7.98E-13 |
| CITED2 | 1792.947 | -1.02071 | 0.137904 | -7.40166 | 1.34E-13 | 8.21E-13 |
| LY6E | 8465.949 | -1.88944 | 0.255371 | -7.39881 | 1.37E-13 | 8.37E-13 |
| DUSP10 | 1838.391 | -1.03653 | 0.1401 | -7.39851 | 1.38E-13 | 8.38E-13 |
| AFM | 9138.768 | -1.79732 | 0.24374 | -7.37392 | 1.66E-13 | 1.00E-12 |
| PCK1 | 47316.67 | -2.12698 | 0.288842 | -7.36382 | 1.79E-13 | 1.08E-12 |
| CD300A | 268.0877 | -1.18077 | 0.16036 | -7.36326 | 1.79E-13 | 1.08E-12 |
| APOBEC3A | 14.65164 | -1.72894 | 0.234899 | -7.36039 | 1.83E-13 | 1.10E-12 |
| HIST1H1C | 6726.015 | 1.565419 | 0.212749 | 7.358038 | 1.87E-13 | 1.12E-12 |
| PRR7 | 126.3334 | 1.365987 | 0.185942 | 7.346318 | 2.04E-13 | 1.22E-12 |
| DUSP5 | 1045.784 | -1.43915 | 0.195968 | -7.34381 | 2.08E-13 | 1.24E-12 |
| SORL1 | 4816.266 | -1.22607 | 0.167628 | -7.31421 | 2.59E-13 | 1.53E-12 |
| CNFN | 44.47053 | 2.139631 | 0.292654 | 7.311126 | 2.65E-13 | 1.56E-12 |
| CP | 40388.53 | -1.44108 | 0.19728 | -7.30478 | 2.78E-13 | 1.63E-12 |
| MYRF | 2654.041 | 1.211767 | 0.166531 | 7.276535 | 3.43E-13 | 1.98E-12 |
| GLS | 1630.994 | 1.200505 | 0.165024 | 7.274734 | 3.47E-13 | 2.00E-12 |
| SLC25A47 | 8396.362 | -2.53152 | 0.347985 | -7.27481 | 3.47E-13 | 2.00E-12 |
| F13A1 | 468.5673 | 1.870599 | 0.257368 | 7.268185 | 3.64E-13 | 2.10E-12 |
| TMSB10 | 21207.82 | 1.364788 | 0.188058 | 7.257267 | 3.95E-13 | 2.27E-12 |
| FHIT | 559.3681 | 1.013015 | 0.139773 | 7.247576 | 4.24E-13 | 2.43E-12 |
| CCDC112 | 109.9469 | 1.154766 | 0.159698 | 7.230943 | 4.80E-13 | 2.73E-12 |
| FBL | 5069.78 | 1.039177 | 0.144081 | 7.212423 | 5.50E-13 | 3.12E-12 |
| SCGN | 621.7264 | 2.301074 | 0.319288 | 7.206896 | 5.72E-13 | 3.24E-12 |
| GC | 132585.5 | -1.13032 | 0.156874 | -7.20523 | 5.79E-13 | 3.28E-12 |
| RRAGD | 1399.968 | 1.090737 | 0.151416 | 7.20357 | 5.87E-13 | 3.32E-12 |
| TSPAN8 | 4030.12 | 1.925722 | 0.269069 | 7.156972 | 8.25E-13 | 4.61E-12 |
| ACACB | 4477.888 | -1.09709 | 0.15344 | -7.14996 | 8.68E-13 | 4.83E-12 |
| SIGLEC1 | 562.0689 | -1.25999 | 0.176255 | -7.14868 | 8.76E-13 | 4.87E-12 |
| HGF | 554.1082 | -1.95001 | 0.273206 | -7.13752 | 9.50E-13 | 5.28E-12 |
| RPL36A | 2539.164 | 1.137497 | 0.159465 | 7.13319 | 9.81E-13 | 5.44E-12 |
| FGB | 373554 | -1.49041 | 0.209035 | -7.12995 | 1.00E-12 | 5.57E-12 |
| MT1E | 9618.565 | -2.46389 | 0.345611 | -7.12908 | 1.01E-12 | 5.60E-12 |
| FGL2 | 816.2346 | -1.19582 | 0.167767 | -7.12789 | 1.02E-12 | 5.64E-12 |
| RHOF | 53.50459 | 1.431916 | 0.200922 | 7.126712 | 1.03E-12 | 5.68E-12 |
| ADH1A | 24041.65 | -1.68689 | 0.236708 | -7.12644 | 1.03E-12 | 5.69E-12 |
| ORM2 | 41663.93 | -1.46041 | 0.205355 | -7.11162 | 1.15E-12 | 6.31E-12 |
| CD14 | 19956.22 | -1.31472 | 0.185249 | -7.09705 | 1.27E-12 | 6.99E-12 |
| THBS1 | 5973.307 | -1.54706 | 0.218146 | -7.09186 | 1.32E-12 | 7.25E-12 |
| ERRFI1 | 14834.63 | -1.24285 | 0.175672 | -7.07486 | 1.50E-12 | 8.16E-12 |
| NNMT | 30914.63 | -2.13144 | 0.301494 | -7.06958 | 1.55E-12 | 8.46E-12 |
| RPL39L | 289.0904 | 1.833797 | 0.259671 | 7.061992 | 1.64E-12 | 8.88E-12 |
| AGRN | 7399.839 | 1.027143 | 0.145458 | 7.061441 | 1.65E-12 | 8.90E-12 |
| HKDC1 | 1739.828 | 1.758139 | 0.249726 | 7.040285 | 1.92E-12 | 1.03E-11 |
| PLAU | 353.6636 | 1.057255 | 0.150418 | 7.028798 | 2.08E-12 | 1.11E-11 |
| MMP9 | 708.6487 | 1.963716 | 0.27957 | 7.024052 | 2.16E-12 | 1.15E-11 |
| MAFF | 1306.149 | -1.11646 | 0.159446 | -7.00213 | 2.52E-12 | 1.33E-11 |
| HES4 | 164.8673 | 1.353705 | 0.193554 | 6.993942 | 2.67E-12 | 1.41E-11 |
| TMEM98 | 2032.337 | 1.33278 | 0.190743 | 6.987293 | 2.80E-12 | 1.48E-11 |
| FKBP11 | 2687.141 | 1.017335 | 0.14612 | 6.962327 | 3.35E-12 | 1.76E-11 |
| CPE | 2641.979 | 1.418923 | 0.204025 | 6.95464 | 3.53E-12 | 1.85E-11 |
| CD226 | 80.1557 | -1.33227 | 0.191756 | -6.94771 | 3.71E-12 | 1.94E-11 |
| GOLM1 | 3993.738 | 1.367926 | 0.197049 | 6.94207 | 3.86E-12 | 2.01E-11 |
| COX6C | 11772.03 | 1.017621 | 0.146794 | 6.932328 | 4.14E-12 | 2.14E-11 |
| AZGP1 | 46336.91 | -1.45985 | 0.210655 | -6.93005 | 4.21E-12 | 2.17E-11 |
| ADGRG6 | 2976.704 | -1.2394 | 0.179032 | -6.92277 | 4.43E-12 | 2.28E-11 |
| VCAN | 1545.366 | 2.035422 | 0.294114 | 6.920531 | 4.50E-12 | 2.31E-11 |
| PPM1K | 452.5696 | -1.17072 | 0.169391 | -6.91135 | 4.80E-12 | 2.46E-11 |
| TMPRSS2 | 1886.075 | -1.1588 | 0.167751 | -6.90783 | 4.92E-12 | 2.52E-11 |
| MTMR7 | 180.7384 | 1.818451 | 0.263785 | 6.893678 | 5.44E-12 | 2.76E-11 |
| RPS21 | 13912.81 | 1.025729 | 0.14894 | 6.886839 | 5.70E-12 | 2.89E-11 |
| CAPG | 1376.281 | 1.431447 | 0.208012 | 6.881558 | 5.92E-12 | 3.00E-11 |
| TMEM71 | 31.80004 | -1.01953 | 0.148201 | -6.87939 | 6.01E-12 | 3.04E-11 |
| FNIP2 | 2183.423 | -1.1486 | 0.167236 | -6.86816 | 6.50E-12 | 3.28E-11 |
| TAX1BP3 | 587.1975 | 1.104384 | 0.160904 | 6.863613 | 6.71E-12 | 3.38E-11 |
| NTN4 | 724.9797 | -1.14688 | 0.167316 | -6.85457 | 7.15E-12 | 3.60E-11 |
| SAA4 | 5643.882 | -1.72926 | 0.252447 | -6.84998 | 7.39E-12 | 3.71E-11 |
| SLC9A9 | 201.7808 | -1.09173 | 0.159691 | -6.83649 | 8.12E-12 | 4.05E-11 |
| SLC38A4 | 14716.86 | -1.39883 | 0.204693 | -6.83382 | 8.27E-12 | 4.11E-11 |
| NFAM1 | 232.6598 | -1.17653 | 0.172958 | -6.80243 | 1.03E-11 | 5.08E-11 |
| ARHGAP4 | 1323.987 | 1.179562 | 0.173415 | 6.801966 | 1.03E-11 | 5.09E-11 |
| UGT2B7 | 16906.45 | -1.71162 | 0.25288 | -6.76851 | 1.30E-11 | 6.35E-11 |
| NDRG1 | 10395.88 | 1.295074 | 0.192014 | 6.74467 | 1.53E-11 | 7.41E-11 |
| PSAT1 | 4394.164 | -1.19027 | 0.177041 | -6.72312 | 1.78E-11 | 8.57E-11 |
| SLC22A1 | 14482.24 | -2.26231 | 0.336703 | -6.71901 | 1.83E-11 | 8.79E-11 |
| TRNP1 | 919.4541 | 1.865202 | 0.277934 | 6.710957 | 1.93E-11 | 9.23E-11 |
| NUPR1 | 12755.7 | 1.177684 | 0.175738 | 6.701342 | 2.07E-11 | 9.81E-11 |
| ADM | 990.5628 | -1.20138 | 0.179394 | -6.69689 | 2.13E-11 | 1.01E-10 |
| NRBP2 | 4865.678 | 1.044427 | 0.156278 | 6.683151 | 2.34E-11 | 1.11E-10 |
| SKAP1 | 454.6248 | -1.44025 | 0.215687 | -6.67751 | 2.43E-11 | 1.15E-10 |
| STX11 | 116.6808 | -1.23545 | 0.185553 | -6.6582 | 2.77E-11 | 1.30E-10 |
| ATP1B3 | 1303.507 | 1.017004 | 0.153228 | 6.637207 | 3.20E-11 | 1.49E-10 |
| SPIC | 9.847791 | -1.75267 | 0.264157 | -6.63495 | 3.25E-11 | 1.51E-10 |
| OXCT1 | 229.9165 | 1.763776 | 0.26585 | 6.634474 | 3.26E-11 | 1.51E-10 |
| HK3 | 177.6178 | -1.29884 | 0.196056 | -6.62483 | 3.48E-11 | 1.61E-10 |
| ALAS1 | 14291.84 | -1.13862 | 0.171917 | -6.62307 | 3.52E-11 | 1.62E-10 |
| MPEG1 | 1200.378 | -1.03092 | 0.155807 | -6.61668 | 3.67E-11 | 1.69E-10 |
| ZEB2 | 628.9341 | -1.04242 | 0.157549 | -6.61651 | 3.68E-11 | 1.69E-10 |
| HAO1 | 9790.76 | -1.34765 | 0.203702 | -6.61581 | 3.70E-11 | 1.70E-10 |
| HIST1H3G | 9.976573 | 3.11627 | 0.471669 | 6.606908 | 3.92E-11 | 1.79E-10 |
| SHMT1 | 13567.84 | -1.16717 | 0.176664 | -6.60672 | 3.93E-11 | 1.79E-10 |
| FGA | 566776.4 | -1.2815 | 0.194263 | -6.59672 | 4.20E-11 | 1.91E-10 |
| MTHFD1 | 12461.87 | -1.11107 | 0.168676 | -6.58703 | 4.49E-11 | 2.03E-10 |
| MBL2 | 5049.501 | -1.72172 | 0.261703 | -6.57893 | 4.74E-11 | 2.14E-10 |
| EIF5A2 | 295.2281 | 1.509859 | 0.229844 | 6.569056 | 5.06E-11 | 2.28E-10 |
| HIST2H2AC | 44.15242 | 1.185386 | 0.180454 | 6.568919 | 5.07E-11 | 2.28E-10 |
| GABRA2 | 29.25741 | 4.807855 | 0.734341 | 6.547168 | 5.86E-11 | 2.62E-10 |
| BCAM | 8431.408 | 1.153302 | 0.176371 | 6.539066 | 6.19E-11 | 2.76E-10 |
| CYP2C9 | 22793.62 | -1.71167 | 0.261819 | -6.53761 | 6.25E-11 | 2.79E-10 |
| C5AR1 | 398.7332 | -1.00261 | 0.153735 | -6.52168 | 6.95E-11 | 3.09E-10 |
| IL4I1 | 182.3086 | 1.607818 | 0.246538 | 6.521582 | 6.96E-11 | 3.09E-10 |
| SRGN | 1592.449 | -1.02141 | 0.156893 | -6.51024 | 7.50E-11 | 3.32E-10 |
| ITLN1 | 41.8194 | -2.46164 | 0.378279 | -6.50749 | 7.64E-11 | 3.38E-10 |
| TMEM154 | 168.7904 | -1.69178 | 0.259981 | -6.50733 | 7.65E-11 | 3.38E-10 |
| SERPINF2 | 60979.06 | -1.15514 | 0.177872 | -6.49426 | 8.34E-11 | 3.67E-10 |
| SLC7A8 | 362.4797 | -1.29445 | 0.199771 | -6.47969 | 9.19E-11 | 4.03E-10 |
| IL10 | 13.9788 | -1.47408 | 0.227875 | -6.46882 | 9.88E-11 | 4.31E-10 |
| FGG | 317343 | -1.29431 | 0.200172 | -6.466 | 1.01E-10 | 4.38E-10 |
| GPR65 | 91.85701 | -1.0796 | 0.16738 | -6.44996 | 1.12E-10 | 4.85E-10 |
| SERPINA10 | 12277.33 | -1.04645 | 0.16263 | -6.43455 | 1.24E-10 | 5.35E-10 |
| TDO2 | 11633.98 | -1.71292 | 0.266526 | -6.42687 | 1.30E-10 | 5.62E-10 |
| SLC1A3 | 332.1485 | 1.288552 | 0.200562 | 6.424724 | 1.32E-10 | 5.69E-10 |
| PLPP2 | 1034.194 | 2.225409 | 0.346652 | 6.419728 | 1.37E-10 | 5.87E-10 |
| CCL4 | 245.9249 | -1.27067 | 0.197967 | -6.41858 | 1.38E-10 | 5.91E-10 |
| THBD | 548.8863 | -1.13156 | 0.176475 | -6.41204 | 1.44E-10 | 6.16E-10 |
| FBLN1 | 2105.412 | 1.624197 | 0.253613 | 6.404243 | 1.51E-10 | 6.46E-10 |
| CHD3 | 1326.903 | 1.083092 | 0.169203 | 6.401158 | 1.54E-10 | 6.59E-10 |
| CXCL2 | 2011.831 | -1.46194 | 0.229144 | -6.37998 | 1.77E-10 | 7.52E-10 |
| VNN2 | 703.6641 | 1.787796 | 0.280862 | 6.365384 | 1.95E-10 | 8.22E-10 |
| HMOX1 | 3570.629 | -1.25325 | 0.197055 | -6.35992 | 2.02E-10 | 8.50E-10 |
| ADH1B | 93680.96 | -1.6944 | 0.267664 | -6.33035 | 2.45E-10 | 1.03E-09 |
| F9 | 12389.61 | -1.76314 | 0.278598 | -6.32862 | 2.47E-10 | 1.03E-09 |
| LRRK2 | 308.1979 | -1.12603 | 0.178242 | -6.31745 | 2.66E-10 | 1.11E-09 |
| HIST1H1E | 11.98163 | 1.51291 | 0.239625 | 6.313659 | 2.73E-10 | 1.13E-09 |
| RIDA | 14794.46 | -1.12564 | 0.178481 | -6.30678 | 2.85E-10 | 1.18E-09 |
| ASPH | 8544.798 | 1.060596 | 0.16827 | 6.302931 | 2.92E-10 | 1.21E-09 |
| PCK2 | 21952.84 | -1.1338 | 0.17992 | -6.3017 | 2.94E-10 | 1.22E-09 |
| RGS18 | 46.10916 | -1.1534 | 0.183174 | -6.29674 | 3.04E-10 | 1.26E-09 |
| SIPA1L2 | 1393.326 | 1.218218 | 0.194337 | 6.268588 | 3.64E-10 | 1.49E-09 |
| PHGDH | 4148.61 | -1.39122 | 0.222253 | -6.25965 | 3.86E-10 | 1.58E-09 |
| EHHADH | 11191.27 | -1.18005 | 0.189386 | -6.23093 | 4.64E-10 | 1.88E-09 |
| GPD1 | 4373.774 | -1.50058 | 0.240929 | -6.22828 | 4.72E-10 | 1.90E-09 |
| ARRDC2 | 2394.631 | 1.092367 | 0.175925 | 6.209278 | 5.32E-10 | 2.14E-09 |
| SOX4 | 1594.59 | 1.367467 | 0.220466 | 6.202617 | 5.55E-10 | 2.22E-09 |
| SPART | 425.7337 | -1.22891 | 0.198736 | -6.18363 | 6.26E-10 | 2.50E-09 |
| MVD | 3209.218 | 1.039805 | 0.168299 | 6.178311 | 6.48E-10 | 2.58E-09 |
| CA2 | 3152.775 | -1.22213 | 0.199455 | -6.12732 | 8.94E-10 | 3.52E-09 |
| CD69 | 124.8636 | -1.2873 | 0.211177 | -6.09585 | 1.09E-09 | 4.25E-09 |
| GATM | 48536.87 | -1.08244 | 0.177675 | -6.09226 | 1.11E-09 | 4.34E-09 |
| SLC27A2 | 7946.427 | -1.21711 | 0.200427 | -6.07261 | 1.26E-09 | 4.89E-09 |
| DBNDD2 | 40.11139 | 1.048649 | 0.173129 | 6.057037 | 1.39E-09 | 5.36E-09 |
| KLRD1 | 90.07184 | -1.18154 | 0.195247 | -6.0515 | 1.44E-09 | 5.53E-09 |
| ACOX2 | 5330.742 | -1.03097 | 0.171536 | -6.01022 | 1.85E-09 | 7.05E-09 |
| FASN | 32427.36 | 1.081708 | 0.180973 | 5.977171 | 2.27E-09 | 8.55E-09 |
| PALM2-AKAP2 | 21.74885 | -1.46772 | 0.245935 | -5.96793 | 2.40E-09 | 9.03E-09 |
| ERICH5 | 1712.267 | 1.669908 | 0.280162 | 5.960517 | 2.51E-09 | 9.42E-09 |
| LAIR2 | 14.13119 | 1.889276 | 0.317254 | 5.955089 | 2.60E-09 | 9.71E-09 |
| ADGRG1 | 1163.779 | 1.208107 | 0.204889 | 5.896407 | 3.72E-09 | 1.37E-08 |
| FCN1 | 173.0861 | -1.2041 | 0.205109 | -5.87053 | 4.34E-09 | 1.60E-08 |
| LILRB1 | 173.8888 | -1.05782 | 0.180472 | -5.86139 | 4.59E-09 | 1.68E-08 |
| TGFBR3 | 1206.892 | -1.0184 | 0.174903 | -5.82264 | 5.79E-09 | 2.11E-08 |
| KDELR3 | 1039.903 | 1.097179 | 0.188493 | 5.820783 | 5.86E-09 | 2.14E-08 |
| TMED3 | 1572.896 | 1.343753 | 0.231295 | 5.809696 | 6.26E-09 | 2.27E-08 |
| KCNJ15 | 81.87353 | -1.30638 | 0.2256 | -5.79069 | 7.01E-09 | 2.52E-08 |
| CPNE5 | 93.21491 | 1.051978 | 0.181878 | 5.783987 | 7.30E-09 | 2.62E-08 |
| FKBP10 | 1374.323 | 1.414655 | 0.244818 | 5.778388 | 7.54E-09 | 2.70E-08 |
| UGT2B10 | 11289.3 | -1.50398 | 0.260725 | -5.76846 | 8.00E-09 | 2.85E-08 |
| NCKAP5 | 131.3732 | -1.17745 | 0.204364 | -5.76154 | 8.34E-09 | 2.96E-08 |
| GADD45G | 3823.222 | -1.24441 | 0.216313 | -5.75279 | 8.78E-09 | 3.11E-08 |
| SLC27A5 | 25141.89 | -1.40416 | 0.244151 | -5.75122 | 8.86E-09 | 3.14E-08 |
| FXYD1 | 1156.195 | -1.71868 | 0.298958 | -5.74891 | 8.98E-09 | 3.17E-08 |
| PKIB | 353.7026 | 1.599312 | 0.278395 | 5.744767 | 9.20E-09 | 3.25E-08 |
| FTCD | 17474.66 | -1.27965 | 0.222864 | -5.74182 | 9.37E-09 | 3.30E-08 |
| SOX9 | 1539.189 | 1.385198 | 0.24225 | 5.718052 | 1.08E-08 | 3.78E-08 |
| TNFSF9 | 37.1649 | 1.526452 | 0.268626 | 5.682433 | 1.33E-08 | 4.62E-08 |
| FCRL6 | 38.09464 | -1.10592 | 0.194899 | -5.67431 | 1.39E-08 | 4.82E-08 |
| HSD17B6 | 22563.8 | -1.27195 | 0.224309 | -5.67051 | 1.42E-08 | 4.93E-08 |
| APOA5 | 13148.4 | -1.39664 | 0.246464 | -5.66672 | 1.46E-08 | 5.02E-08 |
| SULT1A1 | 4568.285 | -1.01238 | 0.178754 | -5.66356 | 1.48E-08 | 5.11E-08 |
| DCDC2 | 1448.56 | 1.777746 | 0.315134 | 5.641243 | 1.69E-08 | 5.79E-08 |
| PRR15L | 332.021 | 1.826689 | 0.324012 | 5.637718 | 1.72E-08 | 5.90E-08 |
| EHF | 330.4964 | 1.577485 | 0.280061 | 5.632653 | 1.77E-08 | 6.05E-08 |
| ALDOB | 210870.7 | -1.43793 | 0.255668 | -5.6242 | 1.86E-08 | 6.34E-08 |
| SRGAP1 | 271.2293 | 1.024537 | 0.182318 | 5.619496 | 1.92E-08 | 6.51E-08 |
| DMKN | 483.3425 | 2.197437 | 0.39131 | 5.615591 | 1.96E-08 | 6.66E-08 |
| DERL3 | 430.8157 | 1.356915 | 0.241677 | 5.614591 | 1.97E-08 | 6.68E-08 |
| TMIGD3 | 147.1598 | -1.11144 | 0.198422 | -5.60142 | 2.13E-08 | 7.17E-08 |
| TTR | 123971.5 | -1.38763 | 0.247771 | -5.60044 | 2.14E-08 | 7.21E-08 |
| FCGR3A | 1520.109 | -1.07777 | 0.192735 | -5.59197 | 2.25E-08 | 7.55E-08 |
| SOX6 | 408.3676 | -1.25104 | 0.223761 | -5.59097 | 2.26E-08 | 7.59E-08 |
| SGK1 | 3206.908 | -1.00514 | 0.180859 | -5.55759 | 2.74E-08 | 9.10E-08 |
| CTNND2 | 423.8537 | 2.555767 | 0.461442 | 5.53865 | 3.05E-08 | 1.01E-07 |
| HSD17B13 | 8997.37 | -2.13053 | 0.384751 | -5.53742 | 3.07E-08 | 1.01E-07 |
| HOPX | 122.008 | 1.136178 | 0.206095 | 5.512896 | 3.53E-08 | 1.16E-07 |
| RAMP1 | 4613.096 | 1.20235 | 0.218362 | 5.506217 | 3.67E-08 | 1.20E-07 |
| AOX1 | 39313.34 | -1.33729 | 0.243179 | -5.49918 | 3.82E-08 | 1.25E-07 |
| FCGBP | 522.6487 | 1.46958 | 0.267593 | 5.491837 | 3.98E-08 | 1.30E-07 |
| JCHAIN | 1021.921 | -1.78066 | 0.324953 | -5.47975 | 4.26E-08 | 1.38E-07 |
| IL2RB | 370.6685 | -1.06192 | 0.194117 | -5.47053 | 4.49E-08 | 1.45E-07 |
| CDA | 947.3766 | -1.24724 | 0.228067 | -5.46872 | 4.53E-08 | 1.47E-07 |
| SLCO1B1 | 6512.272 | -1.19144 | 0.218156 | -5.46142 | 4.72E-08 | 1.52E-07 |
| MYC | 2701.731 | -1.07451 | 0.196829 | -5.45909 | 4.79E-08 | 1.54E-07 |
| SLC44A3 | 733.964 | 1.077333 | 0.197364 | 5.458613 | 4.80E-08 | 1.54E-07 |
| ABCA8 | 1359.115 | -1.37195 | 0.251952 | -5.44527 | 5.17E-08 | 1.66E-07 |
| PRKCB | 190.949 | -1.06835 | 0.196493 | -5.43705 | 5.42E-08 | 1.73E-07 |
| PGLYRP2 | 8227.722 | -1.44249 | 0.265346 | -5.43628 | 5.44E-08 | 1.74E-07 |
| FCGR2B | 325.4575 | -1.5853 | 0.291783 | -5.43313 | 5.54E-08 | 1.77E-07 |
| IFI27L2 | 341.8126 | 1.076633 | 0.198199 | 5.43208 | 5.57E-08 | 1.78E-07 |
| DNAJC12 | 833.9478 | -1.29806 | 0.238958 | -5.43218 | 5.57E-08 | 1.78E-07 |
| SLC46A3 | 2405.321 | -1.2443 | 0.229234 | -5.42809 | 5.70E-08 | 1.81E-07 |
| MUSTN1 | 9.028119 | 1.726626 | 0.31814 | 5.427247 | 5.72E-08 | 1.82E-07 |
| NAALADL2 | 159.4608 | -1.15105 | 0.212112 | -5.42661 | 5.74E-08 | 1.83E-07 |
| TMEM54 | 620.4945 | 1.203509 | 0.221784 | 5.426481 | 5.75E-08 | 1.83E-07 |
| TRPM8 | 1202.121 | -1.35279 | 0.249321 | -5.42592 | 5.77E-08 | 1.83E-07 |
| ANGPTL3 | 17193.87 | -1.05387 | 0.195031 | -5.4036 | 6.53E-08 | 2.07E-07 |
| PON1 | 16301.15 | -1.25739 | 0.232773 | -5.40177 | 6.60E-08 | 2.08E-07 |
| HMGCS2 | 63542.68 | -1.0761 | 0.199438 | -5.39568 | 6.83E-08 | 2.15E-07 |
| GATA3 | 73.50469 | -1.18489 | 0.220017 | -5.38543 | 7.23E-08 | 2.27E-07 |
