# Supplementary material for: Prognostic risk model of LIHC T-cells based on scRNA-seq and RNA-seq and the regulation of the tumor immune microenvironment
Source: Discov Oncol. 2024 Oct 10;15:540. doi: 10.1007/s12672-024-01424-z (PMC11467143; doi:10.1007/s12672-024-01424-z)
Supplement: Supplementary file 5 — Supplementary material 5. [file 12672_2024_1424_MOESM5_ESM.doc]

| **Supplementary Table 4 LASSO regression screening for differential genes** | | | | | | |
| --- | --- | --- | --- | --- | --- | --- |
|  | baseMean | log2FoldChange | lfcSE | stat | pvalue | padj |
| PTTG1 | 703.3985011 | 3.765471351 | 0.190397376 | 19.77690782 | 4.71E-87 | 2.19E-84 |
| TNFRSF4 | 150.4961704 | 2.368045037 | 0.145918325 | 16.22856507 | 3.17E-59 | 7.37E-57 |
| STMN1 | 3378.242792 | 2.216723462 | 0.147861277 | 14.99191346 | 8.29E-51 | 1.29E-48 |
| UBE2S | 681.1105707 | 2.011223632 | 0.147157425 | 13.66715703 | 1.60E-42 | 1.48E-40 |
| ZFP36 | 7516.972317 | -2.049786858 | 0.149856015 | -13.6783756 | 1.37E-42 | 1.48E-40 |
| PHLDA1 | 4515.708599 | -2.419646562 | 0.187061725 | -12.93501684 | 2.86E-38 | 2.21E-36 |
| SERTAD1 | 834.8887028 | -1.517279731 | 0.124532537 | -12.18380164 | 3.79E-34 | 2.52E-32 |
| RTKN2 | 74.13228902 | 3.001394883 | 0.24807645 | 12.09866913 | 1.07E-33 | 6.24E-32 |
| EGR1 | 7737.466491 | -2.53789726 | 0.213556337 | -11.88397075 | 1.43E-32 | 7.41E-31 |
| H2AFZ | 4318.789204 | 1.244185137 | 0.106451686 | 11.68779179 | 1.47E-31 | 6.84E-30 |
| CACYBP | 2460.876127 | 1.132082715 | 0.099086651 | 11.42517887 | 3.13E-30 | 1.32E-28 |
| IER2 | 3315.232664 | -1.283142667 | 0.112653957 | -11.39012518 | 4.68E-30 | 1.81E-28 |
| HSP90AB1 | 45640.73064 | 1.036078393 | 0.092340252 | 11.22022493 | 3.24E-29 | 1.16E-27 |
| HMGB2 | 1987.847213 | 1.458291846 | 0.132717214 | 10.98796303 | 4.37E-28 | 1.45E-26 |
| FOSB | 1935.485241 | -3.18116101 | 0.295510417 | -10.76497078 | 5.04E-27 | 1.56E-25 |
| NEU1 | 5594.310688 | 1.246838239 | 0.116792002 | 10.67571598 | 1.32E-26 | 3.84E-25 |
| FOXO1 | 1615.938102 | -1.639631056 | 0.156435553 | -10.48119193 | 1.05E-25 | 2.88E-24 |
| JUNB | 5307.590176 | -1.482328721 | 0.143222532 | -10.34982902 | 4.19E-25 | 1.08E-23 |
| ID2 | 8228.499505 | -1.366758748 | 0.135943953 | -10.05383998 | 8.84E-24 | 2.16E-22 |
| GBP2 | 3903.36783 | 1.451898222 | 0.149254809 | 9.727647829 | 2.30E-22 | 4.86E-21 |
| CKLF | 271.7537052 | 1.042560385 | 0.108074062 | 9.646721527 | 5.08E-22 | 1.03E-20 |
| NR4A1 | 1607.259989 | -2.066720945 | 0.219519502 | -9.414748713 | 4.74E-21 | 8.48E-20 |
| JUN | 8068.34822 | -1.436648391 | 0.153324884 | -9.369962334 | 7.26E-21 | 1.20E-19 |
| S100A10 | 6194.965784 | 1.313231609 | 0.145582534 | 9.020529968 | 1.87E-19 | 3.00E-18 |
| AQP3 | 3175.388674 | -1.485398487 | 0.165144845 | -8.994519245 | 2.37E-19 | 3.68E-18 |
| SYNE1 | 1216.394112 | -1.214208767 | 0.136125685 | -8.919762432 | 4.67E-19 | 7.01E-18 |
| PKM | 7477.793661 | 1.867964323 | 0.209934154 | 8.897858165 | 5.69E-19 | 8.27E-18 |
| TNFRSF18 | 69.22232705 | 1.915156241 | 0.22049233 | 8.685817973 | 3.76E-18 | 5.14E-17 |
| SERPINH1 | 4379.064814 | 1.144034635 | 0.132146364 | 8.657329679 | 4.83E-18 | 6.42E-17 |
| KLF6 | 6781.841669 | -1.250832456 | 0.145123976 | -8.6190614 | 6.75E-18 | 8.72E-17 |
| RSRP1 | 1083.695952 | 1.008520906 | 0.121023879 | 8.333238971 | 7.87E-17 | 9.38E-16 |
| DUSP5 | 1026.736615 | -1.529231363 | 0.187060248 | -8.175073956 | 2.96E-16 | 3.35E-15 |
| RPL8 | 65050.06139 | 1.127286626 | 0.140920682 | 7.999440622 | 1.25E-15 | 1.38E-14 |
| SPON2 | 8571.152499 | 1.138689163 | 0.146679903 | 7.763089162 | 8.29E-15 | 8.20E-14 |
| NR4A2 | 532.3642403 | -1.812375299 | 0.234077237 | -7.742637954 | 9.74E-15 | 9.43E-14 |
| CCL4 | 234.2818148 | -1.392836692 | 0.182721223 | -7.62274174 | 2.48E-14 | 2.31E-13 |
| SKAP1 | 445.8582341 | -1.53361314 | 0.207465065 | -7.392151242 | 1.44E-13 | 1.24E-12 |
| ZEB2 | 634.0452056 | -1.105928598 | 0.15452298 | -7.157049366 | 8.24E-13 | 6.52E-12 |
| GLS | 1683.739616 | 1.203656942 | 0.168794437 | 7.130904081 | 9.97E-13 | 7.60E-12 |
| LAIR2 | 13.22034959 | 2.238816696 | 0.3139452 | 7.131234041 | 9.95E-13 | 7.60E-12 |
| CITED2 | 1886.20765 | -1.034396168 | 0.147944577 | -6.99178158 | 2.71E-12 | 1.83E-11 |
| KLRD1 | 88.89386811 | -1.2798858 | 0.187899375 | -6.811548994 | 9.66E-12 | 6.15E-11 |
| CD69 | 121.0357572 | -1.338179247 | 0.201649135 | -6.636176493 | 3.22E-11 | 1.86E-10 |
| CCL20 | 1616.786254 | 1.990209635 | 0.308000567 | 6.461707705 | 1.04E-10 | 5.53E-10 |
| GADD45G | 3832.68923 | -1.313614092 | 0.212537096 | -6.180634423 | 6.38E-10 | 3.06E-09 |
| SAMSN1 | 153.7069202 | -1.003963551 | 0.176966378 | -5.673188103 | 1.40E-08 | 5.77E-08 |
| HOPX | 117.7700591 | 1.083729605 | 0.197012732 | 5.50080998 | 3.78E-08 | 1.46E-07 |
| S100A6 | 3661.942152 | 1.185336938 | 0.229743938 | 5.159382867 | 2.48E-07 | 8.66E-07 |
| TNFRSF9 | 78.52168686 | 1.383759587 | 0.277838022 | 4.980454363 | 6.34E-07 | 2.06E-06 |
| SLC38A1 | 1719.128544 | 1.102244477 | 0.222734865 | 4.948684064 | 7.47E-07 | 2.35E-06 |
| BATF | 206.5124329 | 1.063782191 | 0.24828457 | 4.284527996 | 1.83E-05 | 4.63E-05 |
| CTLA4 | 45.66672501 | 1.057400734 | 0.248039499 | 4.263033666 | 2.02E-05 | 4.99E-05 |
