# Supplementary material for: Prognostic risk model of LIHC T-cells based on scRNA-seq and RNA-seq and the regulation of the tumor immune microenvironment
Source: Discov Oncol. 2024 Oct 10;15:540. doi: 10.1007/s12672-024-01424-z (PMC11467143; doi:10.1007/s12672-024-01424-z)
Supplement: Supplementary file 6 — Supplementary material 6. [file 12672_2024_1424_MOESM6_ESM.doc]

| **Supplementary Table 5 Multifactorial Cox regression screening for differential genes** | | | | | |
| --- | --- | --- | --- | --- | --- |
| ID | coef | HR | HR.95L | HR.95H | pvalue |
| PTTG1 | 0.053416 | 1.136 | 1.016 | 1.27 | 0.0025 |
| STMN1 | 0.107892 | 1.157 | 1.019 | 1.315 | 0.025 |
| UBE2S | 0.046867 | 1.22 | 1.054 | 1.412 | 0.008 |
| RTKN2 | 0.276494 | 2.126 | 1.265 | 3.572 | 0.004 |
| CD69 | -0.52761 | 1.233 | 1.017 | 1.494 | 0.033 |
| S100A10 | 0.084581 | 1.129 | 1 | 1.275 | 0.005 |
| CITED2 | 0.150974 | 1.23 | 1.075 | 1.407 | 0.003 |
| SLC38A1 | 0.147608 | 1.18 | 1.007 | 1.384 | 0.004 |
