# Supplementary material for: Prognostic risk model of LIHC T-cells based on scRNA-seq and RNA-seq and the regulation of the tumor immune microenvironment
Source: Discov Oncol. 2024 Oct 10;15:540. doi: 10.1007/s12672-024-01424-z (PMC11467143; doi:10.1007/s12672-024-01424-z)
Supplement: Supplementary file 7 — Supplementary material 7. [file 12672_2024_1424_MOESM7_ESM.doc]

| **Supplementary Table 6 Risk assessment data associated with 8 genes** | | | | | | | | | | | |
| --- | --- | --- | --- | --- | --- | --- | --- | --- | --- | --- | --- |
|  | OS.time | OS | PTTG1 | STMN1 | UBE2S | RTKN2 | S100A10 | CITED2 | SLC38A1 | CD69 | riskScore |
| TCGA.2Y.A9GT.01A | 53.39178082 | 1 | 1.529755309 | 2.845432986 | 1.351893883 | 0.158140622 | 5.340594046 | 4.98101328 | 0.885616219 | 1.381154308 | 1.544494443 |
| TCGA.2Y.A9GU.01A | 63.74794521 | 0 | 2.57598155 | 2.732235092 | 2.089242532 | 0.100073665 | 3.169995636 | 3.77010744 | 0.272284987 | 0.235835822 | 1.940885893 |
| TCGA.2Y.A9GV.01A | 83.24383562 | 1 | 1.024083304 | 2.068629736 | 1.306122287 | 0.041322885 | 4.992597563 | 4.530962367 | 1.477165875 | 0.978086499 | 2.324294623 |
| TCGA.2Y.A9GW.01A | 41.78630137 | 1 | 2.135135352 | 2.792269018 | 1.998802281 | 0.081165172 | 6.344209166 | 3.723177317 | 2.989689882 | 0.941810621 | 2.95438175 |
| TCGA.2Y.A9GX.01A | 80.28493151 | 0 | 1.76843364 | 2.66651769 | 1.886804506 | 0.217190319 | 6.075721399 | 4.370909949 | 2.280195273 | 2.053619187 | 1.870468419 |
| TCGA.2Y.A9GY.01A | 24.88767123 | 1 | 3.761369869 | 4.847938993 | 3.425973333 | 0.606377096 | 7.602642423 | 4.758746005 | 2.932803418 | 1.279358132 | 3.571715852 |
| TCGA.2Y.A9GZ.01A | 27.87945205 | 1 | 1.783542132 | 3.53833636 | 1.417101941 | 0.121580179 | 5.782207748 | 4.034183091 | 0.513099111 | 0.135945946 | 2.480588406 |
| TCGA.2Y.A9H0.01A | 120.8219178 | 0 | 4.332483569 | 5.552579394 | 4.100001169 | 0.718855531 | 6.411413585 | 3.738564486 | 1.936373496 | 0.434497568 | 3.030104691 |
| TCGA.2Y.A9H1.01A | 40.40547945 | 1 | 1.620151762 | 1.585218341 | 2.19078704 | 0.048008499 | 6.62238816 | 1.866687392 | 1.003596724 | 0.155852586 | 2.240909943 |
| TCGA.2Y.A9H2.01A | 56.90958904 | 0 | 2.880529473 | 3.602156712 | 2.516706727 | 0.185787152 | 5.267570596 | 3.285028033 | 4.255647766 | 0.557012602 | 2.884174406 |
| TCGA.2Y.A9H3.01A | 49.84109589 | 0 | 2.112560819 | 2.84115234 | 2.258193522 | 0.091670525 | 6.025000635 | 2.632723816 | 0.752487508 | 0.734255673 | 2.256071895 |
| TCGA.2Y.A9H4.01A | 47.7369863 | 0 | 2.912588695 | 4.438659707 | 3.292462673 | 0.04643039 | 6.611431108 | 4.29041232 | 1.310166793 | 0.37932612 | 2.631066102 |
| TCGA.2Y.A9H5.01A | 18.24657534 | 1 | 2.363634918 | 2.612612859 | 1.218706882 | 0.247728877 | 7.231578948 | 4.505338907 | 0.756826358 | 0.762933071 | 2.650634633 |
| TCGA.2Y.A9H6.01A | 11.7369863 | 0 | 1.525650961 | 2.475440439 | 1.637183283 | 0.103181536 | 4.45869807 | 3.445691133 | 1.132752516 | 1.129155935 | 1.879962047 |
| TCGA.2Y.A9H7.01A | 38.4 | 0 | 2.995390658 | 3.056541856 | 2.068659141 | 0.242646855 | 7.11474159 | 1.84213691 | 1.468542153 | 0.54193911 | 2.271673187 |
| TCGA.2Y.A9H9.01A | 22.91506849 | 0 | 2.772580011 | 3.595221376 | 2.325548424 | 0.04949215 | 6.461825094 | 2.907609594 | 1.76974056 | 0.210249453 | 2.609567052 |
| TCGA.2Y.A9HA.01A | 1.183561644 | 1 | 2.681838642 | 3.564752185 | 2.222788424 | 0.272713017 | 7.08783822 | 3.85003967 | 1.440758366 | 0.176982205 | 3.632955081 |
| TCGA.2Y.A9HB.01A | 8.547945205 | 0 | 2.019840884 | 2.497413908 | 1.568856477 | 0.102554665 | 4.947413196 | 4.955383766 | 0.747571009 | 1.004524855 | 1.908112124 |
| TCGA.3K.AAZ8.01A | 13.01917808 | 0 | 1.362403253 | 2.41110269 | 2.110008398 | 0.068226438 | 6.265339521 | 4.711105749 | 1.109638102 | 1.675522984 | 1.795011559 |
| TCGA.4R.AA8I.01A | 8.61369863 | 1 | 2.692870759 | 3.611101972 | 2.330919428 | 0.272294626 | 5.47241334 | 3.83708087 | 2.520757959 | 0.670803154 | 3.068180036 |
| TCGA.5C.A9VG.01A | 10.78356164 | 0 | 3.420807257 | 3.391034643 | 3.232302256 | 0.052172247 | 3.642395145 | 3.190010508 | 3.111214234 | 1.08725148 | 2.646942531 |
| TCGA.5C.A9VH.01A | 10.58630137 | 0 | 2.553323525 | 3.239315697 | 1.51651097 | 0.150797881 | 7.57079908 | 4.037478561 | 0.47914816 | 1.201745736 | 2.690904937 |
| TCGA.5C.AAPD.01A | 0.657534247 | 0 | 4.111580695 | 4.966696335 | 4.545068631 | 0.159504158 | 7.633379911 | 5.431876516 | 1.532113488 | 1.082869426 | 3.097643878 |
| TCGA.5R.AA1C.01A | 17.09589041 | 0 | 1.9645697 | 3.105044029 | 1.495586672 | 0.079883979 | 6.90322211 | 4.128767238 | 1.430924979 | 0.994022948 | 2.067889574 |
| TCGA.5R.AA1D.01A | 14.76164384 | 0 | 0.874580959 | 3.17249808 | 1.476680717 | 0.093899957 | 5.970020881 | 4.821856546 | 2.372927043 | 1.496309013 | 2.401692822 |
| TCGA.5R.AAAM.01A | 1.512328767 | 1 | 0.941919105 | 2.355320955 | 1.547022635 | 0.053654906 | 5.290212209 | 4.795815769 | 1.300432308 | 1.893226393 | 2.213155694 |
| TCGA.BC.A10X.01A | 25.31506849 | 1 | 0.89733621 | 1.979136455 | 1.370128291 | 0.025373404 | 4.736829702 | 5.361165355 | 1.383945924 | 1.180271826 | 2.089815777 |
| TCGA.BC.A10Z.01A | 1.117808219 | 1 | 2.400059514 | 4.217197515 | 3.202517403 | 0.23738552 | 4.836586706 | 3.85818274 | 0.306451494 | 0.10832291 | 2.176138612 |
| TCGA.BC.A216.01A | 44.41643836 | 0 | 5.314265199 | 5.357007508 | 3.143945159 | 0.466250492 | 4.867531744 | 4.916684069 | 3.650776731 | 1.533672635 | 3.033862508 |
| TCGA.BC.A217.01A | 45.92876712 | 1 | 4.669811562 | 6.37085111 | 3.697997391 | 0.620044506 | 9.243065818 | 4.716998794 | 2.306638142 | 1.275843475 | 3.215320133 |
| TCGA.BC.A3KF.01A | 0.263013699 | 0 | 2.626274676 | 4.077534108 | 2.517319487 | 0.116538642 | 6.78505046 | 3.11752069 | 0.156997502 | 0.661266197 | 2.139632285 |
| TCGA.BC.A3KG.01A | 22.35616438 | 0 | 5.025287993 | 6.274971715 | 3.508102419 | 1.153047038 | 2.549948023 | 5.677250685 | 2.329408758 | 0.906255928 | 3.677107276 |
| TCGA.BC.A5W4.01A | 17.98356164 | 1 | 3.012887192 | 3.25280194 | 3.022158187 | 0.018361795 | 6.608370277 | 3.272708541 | 2.580697459 | 0.036905032 | 2.540365528 |
| TCGA.BC.A69H.01A | 14.59726027 | 0 | 4.963342565 | 4.071316974 | 4.037975303 | 0.361046952 | 7.288217458 | 3.526474278 | 2.352574875 | 0.232662367 | 3.894658082 |
| TCGA.BC.A69I.01A | 12.72328767 | 0 | 1.576871426 | 2.395539194 | 2.174431304 | 0 | 6.749286411 | 2.631939589 | 0.252387979 | 0.256033866 | 3.088353287 |
| TCGA.BC.A8YO.01A | 18.47671233 | 0 | 4.141313071 | 4.488017545 | 3.230825169 | 0.840332876 | 7.21070363 | 5.346925905 | 1.976920413 | 1.053986386 | 3.221794699 |
| TCGA.BD.A3EP.01A | 13.44657534 | 0 | 2.150996439 | 3.193257517 | 2.581707469 | 0.212907682 | 5.258709704 | 3.138335481 | 2.470170093 | 1.211143735 | 2.513160155 |
| TCGA.BD.A3ER.01A | 36.65753425 | 0 | 1.215105189 | 2.043142457 | 1.469342484 | 0.048644389 | 5.738776774 | 2.518676189 | 2.156844841 | 0.460221985 | 2.643418967 |
| TCGA.BW.A5NO.01A | 0.657534247 | 0 | 2.524316737 | 4.511726751 | 2.300938236 | 0.129539524 | 6.719012827 | 4.700696292 | 2.033399932 | 1.691674167 | 2.104622957 |
| TCGA.CC.5258.01A | 4.24109589 | 1 | 3.411149361 | 3.895391672 | 2.408613099 | 0.783900285 | 6.360211506 | 3.68694026 | 3.240154131 | 0.561801306 | 3.49723127 |
| TCGA.CC.5259.01A | 8.219178082 | 0 | 4.03524291 | 2.822058549 | 3.951510309 | 0.149070855 | 6.763839797 | 2.147009004 | 2.79178237 | 0.194813746 | 3.276418874 |
| TCGA.CC.5260.01A | 2.860273973 | 1 | 2.60577385 | 4.091365311 | 3.072169644 | 0.166007332 | 6.849671964 | 2.594720313 | 5.056423559 | 0.906895999 | 3.44329304 |
| TCGA.CC.5261.01A | 3.189041096 | 1 | 3.02530434 | 4.423923848 | 2.355892739 | 0.714313483 | 6.15510302 | 3.734140182 | 3.687435672 | 1.69976301 | 3.002958963 |
| TCGA.CC.5262.01A | 3.38630137 | 1 | 3.456334038 | 4.561910342 | 3.679116136 | 0.203728731 | 6.212380929 | 3.525816683 | 2.880581072 | 0.833555063 | 3.770132459 |
| TCGA.CC.5263.01A | 4.24109589 | 1 | 4.644803357 | 5.380109477 | 2.912151468 | 0.823926247 | 7.250411574 | 5.632819254 | 4.40537072 | 0.401644247 | 4.139775199 |
| TCGA.CC.5264.01A | 3.353424658 | 1 | 4.207112162 | 5.161311543 | 2.736747059 | 0.559416429 | 8.37670283 | 6.16315749 | 3.221829068 | 0.305143885 | 3.769844404 |
| TCGA.CC.A123.01A | 7.2 | 0 | 4.644203156 | 2.654743936 | 3.534986445 | 0.196373548 | 6.122893189 | 1.560125769 | 1.275926332 | 0.30786612 | 2.284779137 |
| TCGA.CC.A1HT.01A | 3.320547945 | 1 | 5.009453513 | 5.127931455 | 3.580552245 | 0.783641551 | 9.308032343 | 4.123453513 | 3.822761247 | 1.67415633 | 3.519000689 |
| TCGA.CC.A3M9.01A | 9.863013699 | 1 | 5.536817404 | 5.715671633 | 5.678860109 | 0.538837379 | 7.19517998 | 3.571863084 | 2.597508982 | 1.642855569 | 3.625370428 |
| TCGA.CC.A3MA.01A | 9.961643836 | 1 | 4.613671438 | 5.488701442 | 3.370409194 | 2.296172278 | 5.726522413 | 2.375778458 | 3.788275249 | 0.326085919 | 4.057503614 |
| TCGA.CC.A3MB.01A | 10.35616438 | 1 | 4.407568642 | 4.200840283 | 3.406016751 | 0.140261197 | 6.456436942 | 3.727713382 | 3.801545042 | 0.29806594 | 3.399498222 |
| TCGA.CC.A3MC.01A | 11.93424658 | 0 | 3.868370164 | 4.02641545 | 3.01179044 | 0.222885441 | 6.15528231 | 3.673395424 | 2.855327538 | 0.242727879 | 3.53745045 |
| TCGA.CC.A5UC.01A | 11.40821918 | 1 | 4.630509859 | 4.206968977 | 4.219712332 | 0.861875734 | 5.984719848 | 3.230967007 | 3.010892511 | 0.342765835 | 3.705067196 |
| TCGA.CC.A5UD.01A | 9.994520548 | 1 | 4.703745166 | 6.040100157 | 3.246374209 | 0.434485952 | 8.916589377 | 3.533592712 | 2.610493973 | 0.282323447 | 4.604428382 |
| TCGA.CC.A5UE.01A | 8.942465753 | 1 | 4.633847505 | 6.040749829 | 4.214500544 | 0.491964322 | 8.370749543 | 4.544581869 | 2.457845065 | 0.485322874 | 3.90304691 |
| TCGA.CC.A7IE.01A | 7.134246575 | 1 | 3.943235306 | 3.280623991 | 3.504784384 | 0.514316808 | 4.535875661 | 3.773659543 | 2.026512957 | 0.940314816 | 3.153140445 |
| TCGA.CC.A7IF.01A | 21.3369863 | 1 | 2.989594203 | 4.207078538 | 2.559419309 | 0.329002852 | 6.577246559 | 1.716438834 | 0.33178038 | 0.258144202 | 2.309565309 |
| TCGA.CC.A7IG.01A | 9.830136986 | 1 | 5.693409849 | 5.668176155 | 4.926194868 | 0.461345936 | 7.157974959 | 2.831834317 | 3.220751652 | 0.201971296 | 4.212934958 |
| TCGA.CC.A7IH.01A | 12 | 0 | 1.985484989 | 3.519937013 | 1.765698074 | 0.162149593 | 5.820181186 | 4.875974315 | 0.313263352 | 0.332436291 | 2.576945675 |
| TCGA.CC.A7II.01A | 13.11780822 | 0 | 5.415139054 | 5.538884299 | 5.123766841 | 0.818100944 | 5.567184587 | 3.824585995 | 3.137844069 | 0.109369275 | 3.864006941 |
| TCGA.CC.A7IJ.01A | 12.55890411 | 0 | 5.472745629 | 5.329331846 | 5.074303563 | 1.005043951 | 7.414065867 | 3.158116422 | 3.920296366 | 1.666474925 | 3.293535794 |
| TCGA.CC.A7IK.01A | 8.61369863 | 1 | 3.579533909 | 4.979073009 | 2.884011707 | 0.34961371 | 6.78408693 | 3.877354271 | 2.784033887 | 0.201315872 | 3.205954098 |
| TCGA.CC.A7IL.01A | 9.139726027 | 1 | 3.012509805 | 2.931603756 | 1.930840508 | 0.24837026 | 6.857987951 | 3.34864606 | 0.014871441 | 0.029895479 | 3.423232449 |
| TCGA.CC.A8HS.01A | 9.863013699 | 1 | 3.954260312 | 5.233976728 | 3.676086374 | 0.890599466 | 6.359193219 | 4.146208281 | 3.221769958 | 0.430394747 | 3.757363011 |
| TCGA.CC.A8HT.01A | 4.602739726 | 1 | 5.354408977 | 5.250239566 | 5.060674014 | 0.348117316 | 6.405942611 | 3.752601437 | 3.879999139 | 1.154944871 | 3.439438538 |
| TCGA.CC.A8HU.01A | 11.30958904 | 1 | 4.081146866 | 6.199779236 | 3.875721795 | 0.808904182 | 8.329287044 | 4.094242456 | 1.222085686 | 0.241472762 | 3.835540912 |
| TCGA.CC.A8HV.01A | 9.17260274 | 1 | 3.749740376 | 3.876814315 | 3.295270261 | 0.567268127 | 7.253961863 | 2.444868761 | 4.156036385 | 0.218081614 | 3.887944716 |
| TCGA.CC.A9FS.01A | 6.936986301 | 0 | 2.223050934 | 3.202720155 | 2.353449963 | 0.154250675 | 6.739888668 | 2.129938661 | 1.786974226 | 0.583077425 | 2.051154303 |
| TCGA.CC.A9FW.01A | 8.153424658 | 0 | 2.866999143 | 2.815684091 | 2.563982316 | 0.555393104 | 6.897105195 | 2.993406762 | 1.657991328 | 0.81989736 | 2.596222755 |
| TCGA.DD.A113.01A | 79.7260274 | 0 | 2.791534179 | 3.662027275 | 2.681021558 | 0.138252204 | 7.116710257 | 3.805966896 | 1.961109684 | 0.576178877 | 2.961301339 |
| TCGA.DD.A114.01A | 37.77534247 | 1 | 4.571581181 | 5.365845921 | 4.299012498 | 0.187171949 | 7.497510943 | 4.93200041 | 3.682829777 | 1.81347432 | 3.228615048 |
| TCGA.DD.A115.01A | 83.57260274 | 1 | 4.093879848 | 4.557000854 | 3.402292853 | 0.338625357 | 6.469428239 | 3.181864583 | 0.63856202 | 0.926257766 | 2.610632765 |
| TCGA.DD.A116.01A | 53.3260274 | 1 | 2.86527335 | 2.942547719 | 2.634974132 | 0.034983353 | 6.459907499 | 2.409863312 | 3.275241442 | 0.132964788 | 2.925526592 |
| TCGA.DD.A118.01A | 112.9972603 | 0 | 2.246982079 | 2.926258912 | 2.453055312 | 0.026980734 | 3.479461967 | 2.762688571 | 0.927998958 | 0.115337403 | 2.350642737 |
| TCGA.DD.A119.01A | 7.331506849 | 1 | 3.18151885 | 3.770862459 | 2.625966639 | 0.077952609 | 6.783840239 | 2.357419671 | 0.5001041 | 0.451970104 | 2.640081744 |
| TCGA.DD.A11A.01A | 2.597260274 | 1 | 1.402512439 | 3.146886961 | 2.277456806 | 0.160598083 | 4.492846384 | 4.234386248 | 2.900056346 | 0.247506258 | 3.079868 |
| TCGA.DD.A11B.01A | 0.460273973 | 1 | 1.65797809 | 2.219449317 | 1.218196435 | 0.125972404 | 6.060006169 | 3.477849515 | 1.810603584 | 0.527190176 | 1.854935488 |
| TCGA.DD.A11C.01A | 21.76438356 | 0 | 3.162062291 | 3.982575206 | 2.52590487 | 0.338237469 | 4.288238908 | 4.463062187 | 0.419299159 | 1.256414402 | 2.665541922 |
| TCGA.DD.A11D.01A | 51.28767123 | 1 | 1.511642494 | 2.236079744 | 1.308125842 | 0.03865048 | 7.270341656 | 4.460502757 | 0.404445896 | 0.375754623 | 2.863615974 |
| TCGA.DD.A1EA.01A | 79.39726027 | 0 | 2.47818983 | 3.114752932 | 2.104696891 | 0.069766326 | 5.556810557 | 3.447263759 | 2.547983543 | 0.869975925 | 2.415801256 |
| TCGA.DD.A1EB.01A | 66.31232877 | 0 | 1.013399893 | 1.292036753 | 1.427422357 | 0.008349822 | 2.232509194 | 5.005603505 | 0.403598828 | 0.349334338 | 2.026534897 |
| TCGA.DD.A1EC.01A | 19.79178082 | 0 | 2.417216006 | 3.43476648 | 3.170673982 | 0.445463455 | 4.339790016 | 4.676518533 | 4.043259702 | 2.663549217 | 1.63530895 |
| TCGA.DD.A1ED.01A | 75.64931507 | 0 | 0.26866944 | 1.672518682 | 0.919812014 | 0.017074003 | 5.18211457 | 4.026952619 | 0.755393465 | 1.93155287 | 1.622295421 |
| TCGA.DD.A1EE.01A | 11.4739726 | 1 | 2.405978844 | 2.966235459 | 2.139410762 | 0.036442617 | 6.208982386 | 3.301364202 | 2.843893699 | 0.147711067 | 3.77918144 |
| TCGA.DD.A1EF.01A | 12.95342466 | 1 | 2.293136992 | 3.961325567 | 2.195305798 | 0.437121009 | 5.038046398 | 4.097514429 | 1.652187586 | 0.971706151 | 2.720000881 |
| TCGA.DD.A1EG.01A | 45.10684932 | 1 | 2.865603073 | 3.447598647 | 2.425122142 | 0.155647465 | 6.744720062 | 3.093714091 | 2.049708906 | 0.808562837 | 3.084222508 |
| TCGA.DD.A1EH.01A | 49.15068493 | 0 | 2.45509005 | 3.220527922 | 1.981555038 | 0.563719068 | 6.96676226 | 2.775624602 | 1.780224425 | 1.260962112 | 2.738615302 |
| TCGA.DD.A1EI.01A | 6.016438356 | 0 | 2.992380097 | 3.029274915 | 2.253323635 | 0.411005243 | 5.811513027 | 3.447502164 | 4.15362687 | 0.787293449 | 3.105194643 |
| TCGA.DD.A1EJ.01A | 33.04109589 | 1 | 3.085218617 | 3.191025772 | 2.519490601 | 0.65118419 | 6.155256703 | 5.05543094 | 0.462310297 | 0.31964617 | 3.420667029 |
| TCGA.DD.A1EK.01A | 18.34520548 | 1 | 2.26058416 | 2.764202077 | 2.358368028 | 0.119855555 | 6.453500331 | 3.878090202 | 0.587460012 | 0.503068205 | 3.167865206 |
| TCGA.DD.A1EL.01A | 13.64383562 | 1 | 4.09677851 | 5.08373296 | 2.749068878 | 0.112233566 | 7.187101699 | 2.845808273 | 2.901089041 | 0.685361896 | 3.67420024 |
| TCGA.DD.A39V.01A | 21.13972603 | 1 | 3.690818762 | 3.035599292 | 3.183382959 | 0.026075882 | 8.172769328 | 2.491145092 | 1.531246481 | 0.347460482 | 3.330010368 |
| TCGA.DD.A39W.01A | 27.1890411 | 1 | 1.662652961 | 2.095954907 | 1.169798148 | 0.035397083 | 5.228749224 | 2.351068007 | 0.216760501 | 0.239239745 | 1.918133668 |
| TCGA.DD.A39X.01A | 55.69315068 | 1 | 2.504986805 | 2.686900385 | 2.82807959 | 0.068924063 | 8.267713077 | 3.627476801 | 1.551762766 | 0.556304903 | 2.943351558 |
| TCGA.DD.A39Y.01A | 5.621917808 | 1 | 5.360525648 | 5.245816116 | 3.993469974 | 0.205249801 | 7.451980215 | 5.036156525 | 2.684867812 | 0.142984193 | 4.210395541 |
| TCGA.DD.A39Z.01A | 19.75890411 | 1 | 3.29034532 | 3.558035798 | 2.74526514 | 0.162199738 | 5.027489042 | 2.491045977 | 1.056636093 | 0.126889088 | 2.599241065 |
| TCGA.DD.A3A1.01A | 7.660273973 | 1 | 3.058192184 | 1.900329083 | 2.367956563 | 0.033387842 | 6.585341878 | 3.117680132 | 2.491196593 | 0.347873748 | 2.803980152 |
| TCGA.DD.A3A2.01A | 70.06027397 | 1 | 0.508299968 | 1.218389807 | 1.029952644 | 0 | 2.063466385 | 5.138801603 | 0.837588114 | 0.336508308 | 1.880811212 |
| TCGA.DD.A3A3.01A | 17.5890411 | 1 | 3.299255218 | 3.168773072 | 2.949716731 | 0.086134333 | 6.515976899 | 2.882119644 | 1.52564822 | 0.12135295 | 2.315082273 |
| TCGA.DD.A3A4.01A | 20.12054795 | 1 | 0.455653588 | 2.064247685 | 1.115611184 | 0.00913055 | 4.223341296 | 4.367959177 | 3.53937742 | 0.040598026 | 3.194145227 |
| TCGA.DD.A3A5.01A | 102.739726 | 1 | 3.484746801 | 4.320307962 | 2.690377906 | 0.176839306 | 7.534493435 | 3.527788233 | 0.11092777 | 0.229860682 | 2.358437688 |
| TCGA.DD.A3A6.01A | 107.1123288 | 1 | 2.022921197 | 3.159036929 | 2.519634027 | 0.111169224 | 4.883878317 | 1.534973333 | 1.132462748 | 1.315981601 | 2.235286006 |
| TCGA.DD.A3A7.01A | 13.77534247 | 1 | 5.03836197 | 3.465185098 | 4.82288974 | 0.264867191 | 6.552528156 | 2.577309872 | 2.842253524 | 0.127831963 | 3.553971028 |
| TCGA.DD.A3A8.01A | 0.361643836 | 1 | 1.727097164 | 2.392925282 | 1.545774543 | 0.002937599 | 6.50685687 | 2.483074953 | 1.509949493 | 0.358114348 | 2.567004788 |
| TCGA.DD.A3A9.01A | 30.60821918 | 1 | 2.209854589 | 2.834184749 | 2.700765345 | 0.067339971 | 4.531043234 | 2.573749973 | 0.958758711 | 0.888712392 | 2.664438888 |
| TCGA.DD.A4NA.01A | 33.13972603 | 0 | 2.070158982 | 2.894710819 | 2.131979732 | 0.139171484 | 5.324316952 | 4.030685094 | 4.617813974 | 0.511149984 | 2.406199177 |
| TCGA.DD.A4NB.01A | 32.51506849 | 0 | 0.920538809 | 2.70998082 | 1.51592323 | 0.142547819 | 5.008146807 | 5.030145597 | 2.795062287 | 1.652870228 | 2.419243443 |
| TCGA.DD.A4ND.01A | 90.27945205 | 0 | 2.825616575 | 3.773419358 | 2.065186468 | 0.496108605 | 6.125274385 | 3.419682918 | 2.93465918 | 1.796696586 | 2.239437001 |
| TCGA.DD.A4NE.01A | 21.69863014 | 1 | 3.859659917 | 4.706900982 | 2.914787262 | 0.227572801 | 5.145156297 | 4.473909538 | 0.876418237 | 0.123205645 | 2.881664354 |
| TCGA.DD.A4NF.01A | 30.96986301 | 0 | 0.41792448 | 1.909875961 | 1.218828216 | 0.025326371 | 5.304293755 | 2.882431144 | 2.374448717 | 0.128134572 | 2.726486149 |
| TCGA.DD.A4NG.01A | 26.36712329 | 1 | 1.815838131 | 2.283048087 | 1.611839154 | 0.223271909 | 6.291968686 | 2.589704786 | 2.006452105 | 0.733843971 | 2.429616023 |
| TCGA.DD.A4NH.01A | 30.14794521 | 0 | 3.646895465 | 3.513766127 | 2.827103368 | 0.557266412 | 6.737381329 | 3.288531254 | 4.931263751 | 0.501784112 | 3.739678214 |
| TCGA.DD.A4NI.01A | 26.82739726 | 0 | 0.85340476 | 2.066286836 | 1.249030301 | 0.107437572 | 5.88713814 | 2.752017994 | 0.764474827 | 1.041435098 | 1.627499658 |
| TCGA.DD.A4NJ.01A | 30.50958904 | 0 | 3.500214608 | 4.359832851 | 2.125274861 | 0.671858756 | 6.472484955 | 2.959105915 | 2.811927484 | 1.194525508 | 2.905650946 |
| TCGA.DD.A4NK.01A | 39.78082192 | 1 | 1.003626588 | 2.187279522 | 1.589530093 | 0.04952908 | 4.296055681 | 3.3847972 | 0.151851894 | 0.305107778 | 1.816713182 |
| TCGA.DD.A4NL.01A | 56.25205479 | 0 | 0.424909323 | 1.589994786 | 0.968812928 | 0.016704865 | 4.804174847 | 3.944534532 | 1.314884195 | 0.717161282 | 2.363189012 |
| TCGA.DD.A4NN.01A | 29.55616438 | 1 | 3.861649107 | 4.161116702 | 2.844847279 | 0.677605706 | 7.4032107 | 3.926530328 | 0.26707573 | 0.120082964 | 3.56014917 |
| TCGA.DD.A4NO.01A | 73.80821918 | 0 | 1.572149226 | 2.119178641 | 2.09848054 | 0.063112767 | 6.250896376 | 2.423430474 | 2.678787091 | 0.589504264 | 2.532804597 |
| TCGA.DD.A4NP.01A | 108.7561644 | 0 | 0.393985924 | 1.645220889 | 1.036187312 | 0.0036689 | 5.384394611 | 3.176603864 | 0.19311283 | 0.335526704 | 1.712741913 |
| TCGA.DD.A4NQ.01A | 12.2630137 | 1 | 3.817933723 | 3.71692166 | 3.594455577 | 0.11688941 | 6.740456581 | 4.949573613 | 3.578671163 | 0.284682005 | 3.860901312 |
| TCGA.DD.A4NR.01A | 0.295890411 | 1 | 3.520291967 | 3.594602129 | 2.832145398 | 0.518864675 | 6.397043877 | 3.669146533 | 3.298567384 | 2.125787733 | 2.587209078 |
| TCGA.DD.A4NS.01A | 80.74520548 | 1 | 1.258005366 | 2.866402358 | 1.835363844 | 0.130990483 | 6.162695556 | 4.084188659 | 1.755162709 | 2.157744792 | 2.095876222 |
| TCGA.DD.A4NV.01A | 78.83835616 | 0 | 0.987533974 | 2.018227778 | 1.970087964 | 0.017926969 | 6.088944327 | 4.565141148 | 0.547443969 | 0.696248206 | 2.095905972 |
| TCGA.DD.A73A.01A | 23.93424658 | 0 | 2.144076944 | 2.845415834 | 1.222476002 | 0.061103447 | 5.764575317 | 3.725776316 | 2.336647864 | 1.505622573 | 2.197911217 |
| TCGA.DD.A73B.01A | 9.304109589 | 1 | 3.38456564 | 3.655072185 | 2.836493998 | 0.423650552 | 5.811352331 | 4.905220895 | 0.943858434 | 0.092109788 | 2.55110483 |
| TCGA.DD.A73C.01A | 23.04657534 | 0 | 0.734501168 | 1.965495804 | 1.038751178 | 0.005374241 | 3.02227627 | 5.342836602 | 0.485717122 | 0.279654899 | 2.10932731 |
| TCGA.DD.A73D.01A | 22.78356164 | 0 | 1.367600043 | 2.499019174 | 1.603205836 | 0.00904145 | 6.303752509 | 3.416398462 | 0.053749964 | 0.069642008 | 2.012697274 |
| TCGA.DD.A73E.01A | 1.446575342 | 0 | 1.385792392 | 2.759253081 | 1.555084429 | 0.003427124 | 7.357854159 | 4.01646275 | 0.137197449 | 0.244186061 | 2.285240823 |
| TCGA.DD.A73F.01A | 35.67123288 | 0 | 2.963058911 | 4.194057462 | 2.404739692 | 0.39398595 | 5.72672691 | 3.957610834 | 2.054463902 | 1.814550982 | 2.212168472 |
| TCGA.DD.A73G.01A | 114.3452055 | 0 | 3.469213641 | 3.22951702 | 4.382815984 | 0.029619532 | 3.59879678 | 1.124146265 | 1.048156288 | 0.049584797 | 2.349281053 |
| TCGA.DD.AA3A.01A | 13.47945205 | 1 | 4.807650181 | 4.723780981 | 3.618697776 | 0.054964595 | 5.972982564 | 2.429741303 | 4.679848876 | 0.403027862 | 3.076483869 |
| TCGA.DD.AAC8.01A | 0.526027397 | 1 | 2.075252437 | 3.135075578 | 1.984900179 | 0.093715559 | 6.666146382 | 2.116081407 | 1.741009364 | 0.25069456 | 3.315111589 |
| TCGA.DD.AAC9.01A | 11.40821918 | 0 | 1.562648549 | 2.328310801 | 1.826324544 | 0.04635793 | 5.858318393 | 3.713644719 | 1.620056628 | 0.534904323 | 2.367001169 |
| TCGA.DD.AACA.01A | 75.64931507 | 0 | 3.178015956 | 3.748274697 | 2.486200587 | 0.324885438 | 6.669732969 | 3.177802597 | 2.45346674 | 0.322038082 | 2.930266662 |
| TCGA.DD.AACB.01A | 76.40547945 | 0 | 3.657160877 | 4.951284728 | 2.41217281 | 0.323921265 | 6.731986543 | 3.859728378 | 1.367653918 | 0.694509318 | 3.206611855 |
| TCGA.DD.AACC.01A | 55.39726027 | 1 | 3.912946186 | 3.433334689 | 2.725968083 | 0.2228032 | 6.319684775 | 2.869986919 | 2.264662946 | 2.130334432 | 2.008519059 |
| TCGA.DD.AACD.01A | 12.5260274 | 1 | 2.914544884 | 3.975829664 | 2.105845536 | 0.085345217 | 5.516677148 | 1.673620922 | 1.696744654 | 0.424855178 | 1.940794775 |
| TCGA.DD.AACE.01A | 71.80273973 | 0 | 1.77233112 | 2.114075368 | 1.20984492 | 0.09000198 | 3.857105531 | 4.450325136 | 0.11820599 | 0.283361168 | 2.15429696 |
| TCGA.DD.AACF.01A | 12 | 1 | 3.896136595 | 4.785001031 | 2.671252458 | 0.289519502 | 7.168439393 | 3.622399075 | 1.606042746 | 0.353324178 | 3.046095519 |
| TCGA.DD.AACG.01A | 15.41917808 | 1 | 5.583345127 | 4.179235443 | 4.18293282 | 0.493530273 | 7.160005504 | 2.712436532 | 1.660781494 | 0.288278062 | 3.048369501 |
| TCGA.DD.AACH.01A | 6.410958904 | 1 | 4.913814436 | 5.14923386 | 3.885392374 | 0.358734951 | 7.719821704 | 4.255503069 | 3.265587971 | 0.371490542 | 3.967836286 |
| TCGA.DD.AACI.01A | 53.19452055 | 0 | 2.883659976 | 2.729379616 | 2.564994965 | 0.125354785 | 7.694709719 | 2.611887877 | 0.911626886 | 1.069696321 | 2.451686049 |
| TCGA.DD.AACJ.01A | 69.10684932 | 0 | 1.776348752 | 1.676895488 | 2.027477518 | 0.008129222 | 6.476874723 | 3.335306848 | 1.41539453 | 0.040649095 | 2.687684896 |
| TCGA.DD.AACK.01A | 0.295890411 | 0 | 2.119961872 | 2.228846975 | 1.773785812 | 0.087523463 | 6.017288525 | 3.371545724 | 0.31641388 | 0.950519334 | 2.327572554 |
| TCGA.DD.AACL.01A | 3.517808219 | 1 | 5.30773494 | 5.525244319 | 4.721131514 | 0.705571601 | 6.378682901 | 3.937364578 | 3.719369968 | 1.034764498 | 3.945195079 |
| TCGA.DD.AACN.01A | 42.80547945 | 0 | 1.820926783 | 3.118476046 | 1.707173166 | 0.150870666 | 6.518791115 | 3.186067243 | 1.64400945 | 2.063444062 | 1.735777298 |
| TCGA.DD.AACO.01A | 61.67671233 | 0 | 4.176483625 | 3.174662598 | 2.136074922 | 0.110734311 | 7.962210618 | 2.042388998 | 0.120249778 | 0.253529462 | 1.891615729 |
| TCGA.DD.AACP.01A | 13.64383562 | 0 | 4.867721941 | 5.151644542 | 4.418527676 | 0.816511133 | 7.60697714 | 2.04925517 | 0.658152058 | 0.324542408 | 3.423550214 |
| TCGA.DD.AACQ.01A | 14.20273973 | 1 | 2.283249447 | 2.802151191 | 2.199822562 | 0.091171115 | 5.223643376 | 4.577256179 | 1.209991325 | 0.090429536 | 2.516173064 |
| TCGA.DD.AACS.01A | 59.30958904 | 0 | 2.575117904 | 4.547179087 | 2.735616406 | 0.333218825 | 7.02073697 | 5.690417625 | 1.164472426 | 0.172036214 | 2.905273875 |
| TCGA.DD.AACT.01A | 51.35342466 | 0 | 2.294936113 | 2.742842604 | 2.282753169 | 0.032640777 | 6.310166351 | 3.987983425 | 0.522371619 | 0.727495278 | 2.381466104 |
| TCGA.DD.AACU.01A | 51.51780822 | 0 | 2.005617164 | 3.248598818 | 1.272183377 | 0.137382893 | 7.438666256 | 3.62730732 | 0.398590385 | 0.775641013 | 2.370169606 |
| TCGA.DD.AACV.01A | 50.33424658 | 0 | 4.325471339 | 5.448128773 | 2.58401219 | 0.785469215 | 7.028333517 | 4.181611973 | 0.396262202 | 0.313832423 | 2.798632962 |
| TCGA.DD.AACW.01A | 46.81643836 | 0 | 3.791610375 | 3.518612871 | 3.008966298 | 0.633966964 | 6.597212602 | 3.879478398 | 0.275528941 | 0.632315183 | 2.613693845 |
| TCGA.DD.AACX.01A | 5.589041096 | 0 | 3.058986196 | 3.648479646 | 2.672729776 | 0.045865284 | 6.755303498 | 3.815056159 | 2.160168272 | 0.15855164 | 2.901194973 |
| TCGA.DD.AACY.01A | 47.67123288 | 0 | 0.820530988 | 1.634846825 | 1.958643748 | 0.002838737 | 4.860777066 | 3.034899526 | 1.423562238 | 0.958243616 | 1.740976747 |
| TCGA.DD.AACZ.01A | 5.621917808 | 1 | 3.526447196 | 4.20491054 | 2.857475829 | 0.684045279 | 7.182842634 | 4.256699285 | 3.523712331 | 2.418509214 | 2.947311538 |
| TCGA.DD.AAD0.01A | 4.504109589 | 0 | 2.922596062 | 2.661752986 | 2.558255826 | 0 | 7.254635586 | 1.873333643 | 0.250115753 | 0.211583739 | 2.185174815 |
| TCGA.DD.AAD1.01A | 18.54246575 | 0 | 1.712908766 | 2.933487711 | 1.682984451 | 0.198108162 | 5.838732517 | 3.219706095 | 2.958880229 | 2.502471771 | 1.700802717 |
| TCGA.DD.AAD2.01A | 21.63287671 | 0 | 3.193676211 | 3.488139428 | 2.279105903 | 0.139536252 | 5.878456494 | 3.406477588 | 1.913953664 | 1.127260632 | 2.536492904 |
| TCGA.DD.AAD3.01A | 42.57534247 | 0 | 1.462014253 | 2.302942304 | 1.582558436 | 0.080075001 | 6.014958839 | 4.849703614 | 1.29960232 | 0.991781965 | 2.455020317 |
| TCGA.DD.AAD5.01A | 44.21917808 | 0 | 4.580163412 | 4.440143433 | 2.782016447 | 0.038091273 | 8.109191322 | 3.68567187 | 3.543245424 | 0.376690868 | 3.120992864 |
| TCGA.DD.AAD6.01A | 22.09315068 | 0 | 3.423701275 | 3.432260682 | 2.382824011 | 0.432291613 | 5.851441765 | 3.050225775 | 1.287343084 | 0.095563972 | 2.952106899 |
| TCGA.DD.AAD8.01A | 40.07671233 | 0 | 2.756068861 | 4.328773432 | 2.46841777 | 0.313463052 | 7.139975768 | 3.110457291 | 1.537883389 | 0.903036339 | 2.789592078 |
| TCGA.DD.AADA.01A | 40.5369863 | 0 | 1.389302976 | 2.124617466 | 0.796625198 | 0.055636661 | 3.75327295 | 2.882762332 | 0.604862731 | 1.297567484 | 1.665452879 |
| TCGA.DD.AADB.01A | 40.83287671 | 0 | 3.563889483 | 3.60719669 | 4.155067295 | 0.843912281 | 4.094955159 | 3.670752708 | 4.095034864 | 1.142142638 | 3.193438101 |
| TCGA.DD.AADC.01A | 13.97260274 | 1 | 5.078465624 | 4.243580616 | 4.290454755 | 0.898148491 | 7.744254264 | 3.162172925 | 3.006268999 | 0.918180571 | 3.586800675 |
| TCGA.DD.AADD.01A | 40.47123288 | 0 | 3.483048388 | 5.104755822 | 3.055971823 | 0.822188369 | 7.164191256 | 2.449954845 | 0.296222567 | 0.55739719 | 2.626133597 |
| TCGA.DD.AADF.01A | 3.780821918 | 1 | 4.43918178 | 5.124693376 | 3.816651249 | 0.213341849 | 7.328355533 | 2.114231353 | 1.461749942 | 0.333722607 | 2.772212797 |
| TCGA.DD.AADG.01A | 37.64383562 | 0 | 1.946657485 | 2.555896586 | 1.786204052 | 0.06217667 | 5.697538984 | 4.833869761 | 0.126932901 | 0.163752544 | 2.162019661 |
| TCGA.DD.AADI.01A | 35.67123288 | 0 | 3.226711352 | 2.807035225 | 2.155769744 | 0.138366748 | 6.386015788 | 2.804932736 | 1.001065341 | 1.091680823 | 2.486279646 |
| TCGA.DD.AADJ.01A | 35.04657534 | 0 | 2.830567463 | 2.67405181 | 2.010600487 | 0.064328816 | 6.181020152 | 2.036629286 | 0.280317944 | 0.547154251 | 1.983792296 |
| TCGA.DD.AADK.01A | 34.48767123 | 0 | 2.784186987 | 2.456100111 | 2.317017581 | 0.108938175 | 6.917982159 | 2.602389562 | 2.619549223 | 0.680999747 | 2.372216641 |
| TCGA.DD.AADL.01A | 20.90958904 | 0 | 2.760201948 | 4.531184508 | 2.474548633 | 0.2661694 | 6.547795253 | 3.468278735 | 2.735565317 | 0.137858648 | 3.050706747 |
| TCGA.DD.AADM.01A | 0.394520548 | 1 | 4.539598935 | 3.670832035 | 3.473832157 | 0.188832947 | 7.275625129 | 3.317702793 | 2.101208957 | 0.265507467 | 2.903612459 |
| TCGA.DD.AADN.01A | 29.52328767 | 0 | 4.779595384 | 4.302059522 | 3.927746556 | 0.129149256 | 7.140495358 | 1.923112236 | 2.567812401 | 0.546407477 | 3.030710976 |
| TCGA.DD.AADO.01A | 14.89315068 | 0 | 4.04991579 | 2.691313153 | 2.778382686 | 0.280328937 | 6.412054323 | 2.031970959 | 1.442533437 | 0.654431136 | 2.394423554 |
| TCGA.DD.AADP.01A | 15.05753425 | 0 | 2.834211322 | 3.425908356 | 2.170863303 | 0.207287528 | 6.820379966 | 3.229157586 | 2.485380408 | 0.652880975 | 2.892868783 |
| TCGA.DD.AADQ.01A | 14.33424658 | 0 | 2.790308428 | 2.690202552 | 2.122517732 | 0.119677068 | 4.60733633 | 1.451390563 | 2.439878778 | 0.27669056 | 2.13607196 |
| TCGA.DD.AADR.01A | 66.6739726 | 0 | 3.728766812 | 3.627740558 | 2.195869902 | 0.319189234 | 7.4542936 | 2.7769682 | 2.651157883 | 0.736494926 | 2.575180618 |
| TCGA.DD.AADS.01A | 15.58356164 | 0 | 1.944224461 | 2.61156658 | 1.734496779 | 0.019556601 | 6.276697661 | 1.562360451 | 0.645885014 | 0.265987964 | 1.606337767 |
| TCGA.DD.AADU.01A | 18.21369863 | 0 | 1.949215754 | 2.282640357 | 1.543007661 | 0.020101304 | 5.594905727 | 2.383741003 | 2.504069327 | 0.151048306 | 2.96331463 |
| TCGA.DD.AADV.01A | 18.87123288 | 0 | 3.019768575 | 4.75241676 | 2.540153001 | 0.365643757 | 6.807235948 | 4.252394318 | 1.042732283 | 0.504096411 | 2.779689969 |
| TCGA.DD.AADW.01A | 19.29863014 | 0 | 2.837762116 | 4.290160804 | 2.86706982 | 1.085288733 | 4.950344199 | 3.398984814 | 2.192588775 | 0.692844896 | 3.132990726 |
| TCGA.DD.AADY.01A | 18.24657534 | 0 | 2.491868288 | 2.993423446 | 1.607793985 | 0.126621443 | 3.527692575 | 4.173553698 | 2.089269148 | 0.242786784 | 2.603384755 |
| TCGA.DD.AAE0.01A | 18.24657534 | 0 | 5.583545166 | 5.55104761 | 3.932657713 | 0.420540643 | 7.890461587 | 2.781462554 | 1.357735852 | 0.279694805 | 3.637848392 |
| TCGA.DD.AAE1.01A | 18.14794521 | 0 | 2.29899404 | 2.091322499 | 1.604690524 | 0.278179614 | 6.939182911 | 2.923803012 | 0.346283574 | 0.570195925 | 2.289609811 |
| TCGA.DD.AAE2.01A | 20.97534247 | 0 | 2.236769525 | 2.846611679 | 2.114628364 | 0.044100162 | 4.610124783 | 5.264440532 | 0.511774945 | 1.403448455 | 1.724228819 |
| TCGA.DD.AAE3.01A | 18.60821918 | 0 | 1.225534951 | 2.573122667 | 1.722005043 | 0.038060667 | 7.039708752 | 3.528405589 | 0.738382252 | 0.150763846 | 2.654544447 |
| TCGA.DD.AAE4.01A | 19.9890411 | 0 | 4.01480664 | 4.421398838 | 2.251858846 | 0.21449692 | 6.177566701 | 4.377985913 | 0.344290854 | 1.266129355 | 2.154704216 |
| TCGA.DD.AAE6.01A | 4.635616438 | 0 | 4.488508775 | 5.52727849 | 3.422391138 | 0.269432414 | 6.548001206 | 2.325210928 | 0.403463381 | 0.010933658 | 2.611972092 |
| TCGA.DD.AAE7.01A | 21.17260274 | 0 | 0.913652961 | 2.219872899 | 2.011180916 | 0.074831509 | 5.371448805 | 3.402558536 | 1.66005341 | 1.271118695 | 1.537794488 |
| TCGA.DD.AAE9.01A | 23.7369863 | 0 | 2.33437465 | 3.677792493 | 2.380655614 | 0.158416537 | 6.476770617 | 2.819605687 | 2.220040839 | 0.11231068 | 2.930872398 |
| TCGA.DD.AAEA.01A | 18.90410959 | 0 | 3.25358027 | 4.399164334 | 3.744548977 | 0.307593391 | 7.000711555 | 2.465993232 | 1.75295873 | 1.346561621 | 2.15026675 |
| TCGA.DD.AAEB.01A | 15.71506849 | 0 | 1.555512581 | 2.354288789 | 1.351767421 | 0.0136535 | 6.477403484 | 2.933128162 | 0.096938506 | 0.458400532 | 1.790032696 |
| TCGA.DD.AAED.01A | 25.08493151 | 0 | 2.612436711 | 2.664815377 | 1.771672098 | 0.162357507 | 7.442390732 | 3.05302538 | 3.868462071 | 0.308924092 | 3.321745401 |
| TCGA.DD.AAEE.01A | 26.63013699 | 0 | 3.548413135 | 4.244530248 | 2.398884014 | 0.207435463 | 6.427832964 | 2.039429073 | 1.711724788 | 0.174793046 | 2.607236754 |
| TCGA.DD.AAEG.01A | 23.63835616 | 0 | 2.717941144 | 3.08287537 | 2.219206992 | 0.085568589 | 6.481968673 | 3.435612072 | 1.835172303 | 0.103524291 | 2.464194463 |
| TCGA.DD.AAEH.01A | 25.77534247 | 0 | 2.56240831 | 4.025396993 | 1.892866019 | 0.092252823 | 3.550249658 | 3.137868756 | 0.85525658 | 1.378310668 | 1.409880051 |
| TCGA.DD.AAEI.01A | 50.33424658 | 0 | 2.702893205 | 2.95922624 | 2.533319783 | 0.011818783 | 6.196833632 | 4.138669051 | 0.857321133 | 0.444168264 | 2.428235326 |
| TCGA.DD.AAEK.01A | 35.07945205 | 0 | 2.249320061 | 2.701112083 | 1.577946706 | 0.147662918 | 6.407930101 | 2.334928071 | 2.59665478 | 1.814930098 | 1.791658639 |
| TCGA.DD.AAVP.01A | 90.47671233 | 0 | 2.172977287 | 3.411103775 | 2.619716623 | 0.010963067 | 6.628869338 | 4.260931832 | 1.327110449 | 0.484004462 | 2.381814034 |
| TCGA.DD.AAVQ.01A | 89.68767123 | 0 | 3.816180227 | 3.610997418 | 3.014894364 | 0.426953141 | 6.577970679 | 3.311956978 | 1.373745675 | 1.876241965 | 2.227027659 |
| TCGA.DD.AAVR.01A | 82.61917808 | 0 | 2.290799418 | 3.476830764 | 1.895905867 | 0.082410026 | 4.657479755 | 3.067024353 | 0.907620334 | 2.00345577 | 1.930889415 |
| TCGA.DD.AAVS.01A | 59.93424658 | 0 | 3.018210682 | 3.478534776 | 1.865046959 | 0.405908717 | 7.228398075 | 3.033846322 | 0.714280526 | 0.725563614 | 2.571857512 |
| TCGA.DD.AAVU.01A | 72.39452055 | 0 | 3.365282629 | 3.774969253 | 2.974697525 | 0.193157759 | 7.009787296 | 1.966010036 | 1.274232132 | 0.033329751 | 2.737383575 |
| TCGA.DD.AAVV.01A | 80.71232877 | 0 | 2.687852992 | 2.971870393 | 3.335211831 | 0.145982276 | 5.952513868 | 4.542219105 | 1.898193428 | 0.974770908 | 2.96713712 |
| TCGA.DD.AAVW.01A | 76.17534247 | 0 | 2.245147516 | 2.765192098 | 1.935847927 | 0.285791072 | 7.012777357 | 3.53816113 | 0.770188359 | 1.432327639 | 2.533446932 |
| TCGA.DD.AAVX.01A | 56.48219178 | 0 | 1.317569501 | 2.370435923 | 1.741853045 | 0.044211756 | 6.406878621 | 2.652493045 | 0.280092584 | 1.018544541 | 1.570779753 |
| TCGA.DD.AAVY.01A | 64.76712329 | 0 | 0.892247604 | 3.016451519 | 1.395462682 | 0.005529457 | 5.107728245 | 2.709189178 | 0.155090648 | 0.19208542 | 1.964702599 |
| TCGA.DD.AAVZ.01A | 62.46575342 | 0 | 2.541161299 | 2.445345183 | 1.443754681 | 0.398072016 | 6.09866514 | 2.57606623 | 0.854836474 | 0.711966818 | 2.109876104 |
| TCGA.DD.AAW0.01A | 66.24657534 | 0 | 1.793454957 | 2.211800061 | 1.810178147 | 0.119023747 | 6.942528048 | 2.507280472 | 1.941494579 | 0.959034422 | 2.159330538 |
| TCGA.DD.AAW1.01A | 65.39178082 | 0 | 0.543987616 | 2.088544874 | 0.869248109 | 0.005150261 | 5.715045854 | 2.785149531 | 2.950536801 | 0.16423635 | 2.536592622 |
| TCGA.DD.AAW2.01A | 60.98630137 | 0 | 1.777678218 | 3.511790576 | 1.14725215 | 0.15547577 | 5.946696114 | 4.149952297 | 0.604146961 | 0.211594893 | 2.569599692 |
| TCGA.DD.AAW3.01A | 53.68767123 | 0 | 1.198380694 | 2.771513532 | 1.774133341 | 0.133644719 | 5.884396357 | 2.946002686 | 0.202098693 | 0.140468099 | 2.030637255 |
| TCGA.ED.A459.01A | 29.91780822 | 0 | 3.433085322 | 2.949466208 | 3.069128349 | 0.085274922 | 4.961946136 | 3.960398877 | 4.150388584 | 0.503042278 | 3.352735253 |
| TCGA.ED.A4XI.01A | 26.9260274 | 0 | 1.028187813 | 2.586669947 | 1.058386255 | 0.046797405 | 4.634909406 | 4.272560886 | 0.880698933 | 1.404745078 | 1.917427423 |
| TCGA.ED.A5KG.01A | 28.07671233 | 0 | 3.367099189 | 4.157887839 | 2.41854352 | 1.193141175 | 5.913871081 | 4.308957518 | 3.932910332 | 3.910131797 | 2.352126797 |
| TCGA.ED.A627.01A | 13.90684932 | 0 | 1.478759913 | 3.148845289 | 1.790542935 | 0.09584765 | 6.12135733 | 4.808672558 | 2.144488435 | 3.453075328 | 1.555131431 |
| TCGA.ED.A66X.01A | 13.34794521 | 0 | 2.677344269 | 3.254258928 | 2.903332666 | 0.569385594 | 5.987340429 | 3.450102069 | 1.952144658 | 0.618623747 | 2.951480141 |
| TCGA.ED.A66Y.01A | 9.731506849 | 1 | 2.813851385 | 3.301804115 | 2.607913951 | 0.302288463 | 2.111925509 | 2.772341963 | 3.247366701 | 0.224539259 | 3.075853229 |
| TCGA.ED.A7PX.01A | 0.197260274 | 0 | 3.004644475 | 3.864735697 | 2.68794875 | 0.097404928 | 5.639660425 | 2.695832583 | 5.388649613 | 0.524560263 | 3.185440708 |
| TCGA.ED.A7PY.01A | 12.82191781 | 0 | 3.086007452 | 3.011715336 | 2.074363046 | 0.263896438 | 6.268461409 | 2.83485603 | 0.154366042 | 0.507473207 | 1.907494655 |
| TCGA.ED.A7PZ.01A | 0.197260274 | 0 | 3.984908834 | 4.781463856 | 4.095109096 | 0.153095704 | 5.577252612 | 3.869652144 | 1.031803945 | 0.119856669 | 3.221701381 |
| TCGA.ED.A7XO.01A | 14.03835616 | 0 | 3.462715453 | 4.637730436 | 3.070385576 | 0.177746081 | 6.977956884 | 4.745664235 | 0.803234175 | 1.271762952 | 2.761337009 |
| TCGA.ED.A7XP.01A | 13.15068493 | 0 | 3.169970042 | 3.587109035 | 2.004402908 | 0.158202085 | 6.7799481 | 4.428926145 | 1.855292727 | 0.633403642 | 3.150456136 |
| TCGA.ED.A82E.01A | 13.41369863 | 0 | 0.904313763 | 2.856068987 | 2.395655425 | 0.041400195 | 5.588872292 | 2.833232686 | 4.616212095 | 0.552097338 | 2.768286451 |
| TCGA.ED.A8O5.01A | 13.34794521 | 0 | 2.777906895 | 3.485678842 | 2.078327374 | 0.488608195 | 4.437976953 | 3.795356491 | 1.864216849 | 0.547266683 | 3.011880234 |
| TCGA.ED.A8O6.01A | 1.84109589 | 1 | 3.114420458 | 3.4290279 | 2.499563023 | 0.506873919 | 6.549884529 | 3.587946537 | 1.263594863 | 0.221324939 | 2.948114823 |
| TCGA.ED.A97K.01A | 0.197260274 | 0 | 2.441022065 | 3.21714669 | 2.514204918 | 0.601059391 | 7.492095663 | 4.120506258 | 3.614206316 | 0.901292998 | 3.131712388 |
| TCGA.EP.A12J.01A | 18.73972603 | 0 | 1.597187284 | 2.554490392 | 1.350134058 | 0.045038875 | 6.526784968 | 3.114364533 | 1.550319742 | 0.245270685 | 2.158656931 |
| TCGA.EP.A26S.01A | 19.9890411 | 0 | 1.561706821 | 2.681614864 | 1.421491392 | 0.022067602 | 4.408672896 | 3.520189612 | 1.36802821 | 0.16956612 | 2.086485394 |
| TCGA.EP.A2KA.01A | 20.61369863 | 1 | 4.05517721 | 4.288200979 | 2.830855759 | 0.096653608 | 6.183717964 | 3.78043306 | 2.181108882 | 1.690766394 | 2.418622624 |
| TCGA.EP.A2KB.01A | 19.59452055 | 1 | 4.099996086 | 3.562066637 | 2.5726237 | 0.622673151 | 7.250209938 | 3.56679884 | 0.766951342 | 0.507480309 | 2.936814212 |
| TCGA.EP.A2KC.01A | 0.624657534 | 1 | 2.466403981 | 2.844360521 | 1.771963869 | 0.074671516 | 6.196511554 | 4.034340887 | 1.782262781 | 0.421873245 | 3.256871508 |
| TCGA.EP.A3JL.01A | 9.961643836 | 0 | 2.828950517 | 3.388039595 | 2.661120863 | 0.166180413 | 6.204019319 | 2.956099837 | 1.710475968 | 1.297871476 | 2.272620923 |
| TCGA.EP.A3RK.01A | 11.93424658 | 0 | 3.785819655 | 3.794021863 | 2.932419225 | 0.141767836 | 6.394270448 | 3.10030347 | 2.673292969 | 1.633512644 | 2.692502078 |
| TCGA.ES.A2HS.01A | 22.61917808 | 1 | 2.110977733 | 3.098015449 | 1.995471425 | 0.002958171 | 5.907111938 | 1.619119002 | 1.129482498 | 0.187010454 | 1.579828825 |
| TCGA.ES.A2HT.01A | 14.4 | 1 | 0.979175031 | 1.696861937 | 2.218746852 | 0.007188032 | 6.965914828 | 3.836105979 | 0.459013893 | 0.258777413 | 2.153275813 |
| TCGA.FV.A23B.01A | 60.88767123 | 1 | 2.875742982 | 3.209047304 | 2.736627606 | 0.552989806 | 5.745496826 | 2.559486094 | 2.033983395 | 0.726748404 | 2.984047115 |
| TCGA.FV.A2QQ.01A | 23.96712329 | 0 | 2.432229851 | 2.529713388 | 2.314842917 | 0.053753752 | 7.149182201 | 3.239325547 | 1.995225972 | 0.783058105 | 3.347237891 |
| TCGA.FV.A2QR.01A | 19.10136986 | 1 | 2.368608636 | 2.76618938 | 2.164626241 | 0.086921411 | 7.326571529 | 2.88832362 | 3.050028222 | 0.15771117 | 3.569009401 |
| TCGA.FV.A3I0.01A | 27.87945205 | 0 | 2.231715746 | 4.00443067 | 2.404453895 | 0.066588791 | 6.104918081 | 3.046548313 | 4.833405397 | 0.672294738 | 2.968390631 |
| TCGA.FV.A3I1.01A | 8.120547945 | 1 | 2.585249195 | 3.628810546 | 2.471421617 | 0.424636777 | 6.008103231 | 3.946506814 | 1.339948687 | 1.033595812 | 2.792192922 |
| TCGA.FV.A3R2.01A | 6.378082192 | 1 | 2.984938782 | 3.966662912 | 2.795755842 | 0.134012949 | 5.273330039 | 3.742283698 | 3.613076003 | 0.245818103 | 3.314822171 |
| TCGA.FV.A3R3.01A | 12.03287671 | 1 | 0.626375616 | 2.833803618 | 1.268627284 | 0.018852908 | 6.247457939 | 4.450986812 | 1.137687488 | 1.144071228 | 2.651606968 |
| TCGA.FV.A495.01A | 0.032876712 | 0 | 4.999776759 | 4.588153569 | 4.335334056 | 0.18121684 | 6.668871 | 3.235637344 | 1.030813939 | 0.847644142 | 2.58594808 |
| TCGA.FV.A496.01A | 0.328767123 | 0 | 1.611850002 | 2.300433086 | 2.08391092 | 0.053281992 | 4.039392171 | 3.780304079 | 2.779330013 | 0.076369815 | 2.611789418 |
| TCGA.FV.A4ZP.01A | 81.73150685 | 1 | 5.137480464 | 2.274151085 | 5.31384854 | 0.04009995 | 8.680824344 | 2.71979222 | 1.552009987 | 0 | 3.100507619 |
| TCGA.FV.A4ZQ.01A | 0.394520548 | 0 | 4.555753071 | 5.493171567 | 2.828715352 | 0.672160921 | 5.953809434 | 3.889054148 | 2.160581475 | 1.585359157 | 2.66969295 |
| TCGA.G3.A25S.01A | 13.67671233 | 1 | 4.698239625 | 4.171238775 | 4.039695234 | 0.508296716 | 6.765681722 | 3.176881058 | 2.681576315 | 0.423815951 | 3.710129777 |
| TCGA.G3.A25T.01A | 51.05753425 | 0 | 4.111262493 | 4.787733875 | 3.74170845 | 0.135794587 | 8.052652304 | 2.591836733 | 3.858675299 | 0.387975669 | 3.072619467 |
| TCGA.G3.A25U.01A | 53.78630137 | 0 | 2.601955392 | 3.481832665 | 1.862961113 | 0.013585708 | 5.729857923 | 4.200877423 | 0.913782515 | 0.364111296 | 2.292047206 |
| TCGA.G3.A25V.01A | 28.2739726 | 0 | 1.355619868 | 2.256972639 | 1.437313522 | 0.059414496 | 5.559684148 | 3.828638112 | 1.60087227 | 1.089175538 | 2.152429898 |
| TCGA.G3.A25X.01A | 58.48767123 | 0 | 4.686630494 | 3.925853011 | 4.033212932 | 0.173451204 | 8.929821688 | 3.301302244 | 3.295866696 | 0.941230205 | 2.763221511 |
| TCGA.G3.A25Y.01A | 14.86027397 | 1 | 3.848500785 | 4.184039811 | 2.71546273 | 0.441526988 | 5.117081304 | 2.728209781 | 2.83818808 | 1.024299058 | 2.987220792 |
| TCGA.G3.A25Z.01A | 21.53424658 | 0 | 2.316857106 | 2.605166544 | 2.018710358 | 0.080924149 | 6.266913261 | 3.8472976 | 2.523373466 | 0.113570295 | 2.926102727 |
| TCGA.G3.A3CG.01A | 22.1260274 | 0 | 1.618185667 | 2.501396561 | 1.663722457 | 0.039485932 | 3.246615061 | 3.876845858 | 1.904568125 | 0.658379771 | 2.364008252 |
| TCGA.G3.A3CH.01A | 25.64383562 | 0 | 2.208004516 | 3.846275328 | 2.367338692 | 0.043732734 | 6.408968381 | 3.023798739 | 2.083186903 | 0.793497819 | 2.175391411 |
| TCGA.G3.A3CI.01A | 5.917808219 | 0 | 0.883033138 | 1.383367454 | 1.381664533 | 0.024837541 | 3.642030588 | 3.771453196 | 1.673307553 | 0.76769856 | 1.878376768 |
| TCGA.G3.A3CJ.01A | 19.52876712 | 0 | 1.092504401 | 2.110576193 | 2.067006444 | 0.002820566 | 6.752150457 | 3.129973869 | 0.042436475 | 0.065138366 | 2.889218413 |
| TCGA.G3.A3CK.01A | 19.23287671 | 0 | 1.677617733 | 2.572118531 | 1.380036208 | 0.039542014 | 6.080645928 | 2.777260842 | 1.396559333 | 0.670561953 | 1.98258471 |
| TCGA.G3.A5SI.01A | 25.24931507 | 1 | 3.922415606 | 4.52248609 | 3.033269429 | 0.358143224 | 5.656729476 | 2.872087461 | 0.211488917 | 0.03562377 | 2.656035747 |
| TCGA.G3.A5SJ.01A | 22.94794521 | 0 | 3.062327885 | 4.803154926 | 2.472675206 | 0.278218153 | 7.626998182 | 5.825437257 | 3.27808079 | 1.772914046 | 3.411297781 |
| TCGA.G3.A5SK.01A | 24.46027397 | 0 | 0.723314331 | 1.620548573 | 0.943976697 | 0.020338811 | 3.889706205 | 4.125358406 | 1.275744637 | 1.060035286 | 1.404471127 |
| TCGA.G3.A5SL.01A | 20.41643836 | 0 | 1.695545037 | 2.810886184 | 1.245251585 | 0.072187187 | 5.335586254 | 3.896135002 | 0.84848104 | 0.465267179 | 2.315327294 |
| TCGA.G3.A5SM.01A | 17.09589041 | 0 | 2.66502642 | 2.953810003 | 2.007393906 | 0.101654216 | 5.860095448 | 3.863680998 | 1.959950625 | 0.423916793 | 2.806788944 |
| TCGA.G3.A6UC.01A | 22.06027397 | 0 | 1.018000646 | 2.664605387 | 1.249449943 | 0 | 5.576550613 | 4.376966564 | 0.175981164 | 0.094192743 | 1.961977988 |
| TCGA.G3.A7M5.01A | 14.69589041 | 0 | 1.615728102 | 2.359374269 | 2.375248399 | 0.070042522 | 6.398036625 | 4.593853342 | 1.391990479 | 0.490908465 | 2.280127159 |
| TCGA.G3.A7M6.01A | 20.77808219 | 0 | 2.1817119 | 4.566600576 | 3.18098711 | 0.382562911 | 6.998651438 | 4.799888641 | 4.535344021 | 1.486101352 | 3.839499641 |
| TCGA.G3.A7M7.01A | 11.86849315 | 0 | 0.967165132 | 2.343938874 | 1.725125927 | 0.016047853 | 5.670512676 | 3.114248449 | 2.364098776 | 0.183661948 | 2.276474125 |
| TCGA.G3.A7M8.01A | 14.1369863 | 0 | 0.523115837 | 2.08236339 | 0.799824824 | 0.011513585 | 4.945938457 | 3.370219473 | 0.28416947 | 0.094283462 | 2.251536804 |
| TCGA.G3.A7M9.01A | 1.84109589 | 1 | 4.958711158 | 5.386960042 | 4.791723489 | 0.855645938 | 7.789211757 | 5.906063854 | 3.501876379 | 0.317775719 | 4.568340691 |
| TCGA.G3.AAUZ.01A | 15.78082192 | 0 | 3.874770386 | 4.438143496 | 3.170829078 | 0.101718728 | 7.007134368 | 3.517967816 | 1.484025818 | 0.474354135 | 2.747075625 |
| TCGA.G3.AAV0.01A | 15.64931507 | 0 | 1.858326961 | 1.842823398 | 2.764175405 | 0.020421936 | 4.354140431 | 3.324921269 | 1.343739298 | 0.272951097 | 1.873063515 |
| TCGA.G3.AAV1.01A | 11.80273973 | 1 | 2.317308794 | 2.672476809 | 1.693190401 | 0.061581354 | 5.863734078 | 2.681039708 | 2.728784381 | 0.970020741 | 3.301334488 |
| TCGA.G3.AAV2.01A | 12.23013699 | 0 | 0.676997944 | 1.753781322 | 1.602289842 | 0.011703079 | 5.259135428 | 3.979931954 | 1.57724523 | 0.105029253 | 2.617548171 |
| TCGA.G3.AAV3.01A | 13.54520548 | 0 | 2.704810407 | 3.95755704 | 2.272977901 | 0.199260668 | 5.293439165 | 2.775991944 | 1.163867939 | 1.164294409 | 2.154279452 |
| TCGA.G3.AAV4.01A | 0.887671233 | 1 | 2.739180379 | 3.9843188 | 2.07701955 | 0.010448131 | 6.63333381 | 2.27825509 | 1.38401847 | 1.159257278 | 2.297932878 |
| TCGA.G3.AAV5.01A | 11.63835616 | 0 | 3.353655919 | 3.653139669 | 2.861029275 | 0.004938797 | 7.047279865 | 3.230952584 | 0.18769714 | 0.587767914 | 2.893228518 |
| TCGA.G3.AAV6.01A | 2.136986301 | 1 | 4.920728988 | 4.960006669 | 4.399564897 | 0.857982646 | 7.375370397 | 2.948019097 | 0.172636408 | 0.449158182 | 3.33251712 |
| TCGA.G3.AAV7.01A | 11.86849315 | 0 | 5.032509502 | 5.560094788 | 4.721823675 | 1.154027805 | 7.534869689 | 4.83521724 | 4.115829605 | 0.618733637 | 4.129109887 |
| TCGA.GJ.A3OU.01A | 28.89863014 | 0 | 3.446929532 | 3.74761549 | 2.953208177 | 0.430871283 | 7.220789106 | 4.117672742 | 3.114614954 | 2.270701288 | 2.807030206 |
| TCGA.GJ.A6C0.01A | 1.019178082 | 1 | 3.638340941 | 4.070722579 | 3.596732077 | 0.358528165 | 7.284836573 | 5.919142416 | 2.966866615 | 0.649127172 | 3.562410172 |
| TCGA.GJ.A9DB.01A | 2.202739726 | 1 | 2.638506128 | 4.015166989 | 2.456472932 | 0.057136027 | 6.520193303 | 3.354873865 | 2.375009031 | 0.696416671 | 2.909446073 |
| TCGA.HP.A5MZ.01A | 2.991780822 | 1 | 2.256088437 | 2.914631183 | 1.963154567 | 0.148262272 | 6.813941715 | 3.783296498 | 1.43532224 | 1.47790383 | 2.699814406 |
| TCGA.K7.A5RF.01A | 20.74520548 | 0 | 0.594071048 | 1.797459036 | 1.544571158 | 0.03532647 | 5.093613544 | 4.878448119 | 1.330253522 | 1.560748003 | 1.710131293 |
| TCGA.K7.A5RG.01A | 17.0630137 | 0 | 3.348058584 | 4.266897683 | 2.350843346 | 0.369039829 | 6.14301717 | 4.927557801 | 2.720880588 | 2.849551944 | 1.747541932 |
| TCGA.K7.A6G5.01A | 16.83287671 | 0 | 2.40265261 | 2.55364264 | 2.27840858 | 0.033411538 | 5.458987824 | 3.489180919 | 0.587441075 | 0.689015728 | 2.353046346 |
| TCGA.K7.AAU7.01A | 11.80273973 | 0 | 3.152957076 | 3.985112126 | 3.200073236 | 0.316153911 | 6.637050685 | 5.047788428 | 4.726874174 | 1.586036307 | 3.210591289 |
| TCGA.KR.A7K0.01A | 2.136986301 | 1 | 2.18661732 | 2.892701851 | 1.955669862 | 0.070692588 | 6.271623324 | 3.577499645 | 0.891866985 | 0.647863444 | 2.785284971 |
| TCGA.KR.A7K2.01A | 27.25479452 | 0 | 2.495742292 | 3.524785948 | 2.804955257 | 0.193822597 | 7.439349405 | 3.440268568 | 1.094810453 | 1.264483918 | 2.524253386 |
| TCGA.KR.A7K7.01A | 31.26575342 | 0 | 4.804411452 | 4.445352681 | 4.475515909 | 0.194916333 | 6.064739919 | 3.550959921 | 1.564606079 | 0.760249484 | 3.303243148 |
| TCGA.KR.A7K8.01A | 29.78630137 | 0 | 3.099072935 | 3.590111151 | 3.310563966 | 0.224323022 | 5.632417048 | 3.444465402 | 0.771951715 | 0.78015397 | 2.419387697 |
| TCGA.LG.A6GG.01A | 12.72328767 | 0 | 2.699821448 | 3.157711188 | 2.120469163 | 0.20409903 | 6.53928548 | 3.984913271 | 0.339506075 | 0.260065335 | 2.82199412 |
| TCGA.LG.A9QC.01A | 13.97260274 | 0 | 2.349690377 | 2.253039448 | 2.10676389 | 0.017397482 | 5.962643289 | 4.885986106 | 0.979344474 | 0.228715818 | 2.674347865 |
| TCGA.LG.A9QD.01A | 12.03287671 | 0 | 0.672966603 | 1.664597144 | 1.395449555 | 0.015412676 | 5.008662736 | 2.078207909 | 2.02751761 | 0.597406226 | 1.673610497 |
| TCGA.MI.A75C.01A | 9.567123288 | 0 | 2.310777541 | 2.962753066 | 2.386925257 | 0.072525233 | 7.345790638 | 3.012256913 | 3.084882362 | 0.50300676 | 3.100067478 |
| TCGA.MI.A75E.01A | 16.66849315 | 0 | 2.136123185 | 2.926307263 | 2.053266498 | 0.011274985 | 5.824931908 | 3.040059611 | 1.295639679 | 0.994181838 | 2.296930249 |
| TCGA.MI.A75G.01A | 22.94794521 | 0 | 1.619121588 | 2.670944121 | 2.77636217 | 0.034159673 | 6.141455274 | 4.239506815 | 2.402091812 | 0.161003743 | 2.948553392 |
| TCGA.MR.A520.01A | 7.528767123 | 0 | 0.465760194 | 1.315283599 | 0.766861429 | 0.011543656 | 4.098560543 | 3.862756156 | 1.732301773 | 0.35345863 | 1.45751621 |
| TCGA.MR.A8JO.01A | 10.84931507 | 0 | 1.899525134 | 3.038747538 | 2.577308556 | 0.226516436 | 5.440163344 | 4.42676403 | 2.841499458 | 1.806953807 | 2.629043833 |
| TCGA.NI.A4U2.01A | 58.88219178 | 1 | 1.39583062 | 1.71499504 | 1.131069356 | 0.020581927 | 3.393417384 | 3.54157374 | 2.311259242 | 0.176566501 | 2.224855549 |
| TCGA.NI.A8LF.01A | 26.26849315 | 0 | 2.163591181 | 2.44222914 | 1.592250275 | 0.050856965 | 6.180185234 | 3.440759024 | 1.023646001 | 0.374480675 | 2.550857702 |
| TCGA.O8.A75V.01A | 17.68767123 | 0 | 2.779137151 | 3.330532528 | 2.040458072 | 0.089110028 | 6.308038144 | 2.930177576 | 1.115863656 | 0.567417932 | 2.377898827 |
| TCGA.PD.A5DF.01A | 21.00821918 | 1 | 3.079566832 | 3.950608579 | 2.414713655 | 0.131765896 | 6.815457671 | 4.238720956 | 3.082025414 | 0.48099486 | 3.281722661 |
| TCGA.QA.A7B7.01A | 3.090410959 | 0 | 4.64557929 | 4.628742125 | 3.338330147 | 0.651963888 | 3.038524006 | 4.953405326 | 2.190641561 | 0.381229812 | 2.797447009 |
| TCGA.RC.A6M4.01A | 0.723287671 | 0 | 2.46963182 | 4.173213254 | 1.387296867 | 0.058710945 | 5.344897303 | 3.124633052 | 0.456512687 | 0.03984954 | 2.26393495 |
| TCGA.RC.A6M5.01A | 0.493150685 | 0 | 0.466917434 | 2.648065675 | 1.442270397 | 0.016510409 | 5.666606079 | 3.896377672 | 2.676387038 | 0.426214465 | 2.553588248 |
| TCGA.RC.A6M6.01A | 0.295890411 | 0 | 4.526109969 | 3.235605885 | 3.083443773 | 0.239794925 | 6.836875701 | 4.058103474 | 3.299524864 | 0.212583991 | 3.434831858 |
| TCGA.RC.A7S9.01A | 21.04109589 | 0 | 4.072590864 | 4.761474139 | 3.889082956 | 0.168844219 | 5.480280336 | 2.801961425 | 3.506065293 | 0.392285573 | 3.154846689 |
| TCGA.RC.A7SB.01A | 19.33150685 | 0 | 2.700219342 | 4.023529922 | 2.091765134 | 0.137271935 | 6.162587998 | 4.174015648 | 2.080335693 | 0.305884828 | 3.19684554 |
| TCGA.RC.A7SF.01A | 19.03561644 | 0 | 3.177459297 | 3.024501804 | 2.575345099 | 0.077827822 | 6.227575502 | 4.317834376 | 0.306431896 | 0.757550186 | 2.318903534 |
| TCGA.RC.A7SH.01A | 15.38630137 | 0 | 4.391538488 | 3.144715733 | 3.392952019 | 0.479075804 | 7.67469537 | 3.010977911 | 2.140584209 | 0.267361259 | 3.118747991 |
| TCGA.RC.A7SK.01A | 15.51780822 | 0 | 2.157119222 | 3.477454758 | 1.490627805 | 0.108863107 | 6.93969356 | 3.46131748 | 1.234307451 | 0.139645464 | 2.508936863 |
| TCGA.RG.A7D4.01A | 36.09863014 | 0 | 4.720469218 | 4.755526985 | 3.03105648 | 0.379747466 | 6.291419723 | 2.499239345 | 1.278262263 | 1.779457917 | 2.317982762 |
| TCGA.UB.A7MA.01A | 27.87945205 | 0 | 4.063336398 | 4.424024496 | 2.426782116 | 0.196945682 | 7.387333046 | 3.055797139 | 4.155666262 | 0.413004482 | 3.367055765 |
| TCGA.UB.A7MB.01A | 19.75890411 | 0 | 3.573463513 | 4.517692624 | 2.825452511 | 0.559424691 | 6.617365543 | 5.007459236 | 2.275418406 | 0.129016875 | 3.046021574 |
| TCGA.UB.A7MC.01A | 16.43835616 | 0 | 3.183525943 | 3.920912914 | 2.328798343 | 0.230823773 | 6.271410037 | 5.250869177 | 1.523409011 | 0.113834507 | 2.938020487 |
| TCGA.UB.A7MD.01A | 1.709589041 | 1 | 2.195161102 | 3.356863564 | 2.63003875 | 0.0506553 | 7.464248016 | 4.991613417 | 1.076040657 | 0.915241618 | 3.019700778 |
| TCGA.UB.A7ME.01A | 15.97808219 | 0 | 2.806465519 | 3.666940079 | 2.113045507 | 0.228902897 | 6.937645844 | 3.218035169 | 1.544321861 | 1.275357552 | 2.236033276 |
| TCGA.UB.A7MF.01A | 7.035616438 | 1 | 3.859235962 | 4.098563541 | 3.495190687 | 0.268284229 | 7.5126434 | 3.438789974 | 0.582502299 | 2.135210917 | 1.938275956 |
| TCGA.UB.AA0U.01A | 10.75068493 | 0 | 2.77126113 | 3.79180625 | 1.988767754 | 0.161450292 | 6.367272457 | 3.542014514 | 2.354000269 | 1.320236078 | 2.509489489 |
| TCGA.UB.AA0V.01A | 10.32328767 | 0 | 0.565726851 | 1.899228188 | 1.056320151 | 0.020713241 | 4.772199789 | 3.622712038 | 2.080253695 | 1.024031603 | 1.843670933 |
| TCGA.WJ.A86L.01A | 11.34246575 | 0 | 2.505504444 | 2.483108625 | 2.745737865 | 0.297555581 | 6.371392635 | 4.111580058 | 2.596274801 | 0.351414035 | 2.785967737 |
| TCGA.WQ.AB4B.01A | 12.98630137 | 0 | 3.058406296 | 2.753271719 | 2.328788082 | 0.058206794 | 6.97177615 | 2.160646699 | 2.239645232 | 0.480330995 | 2.393803482 |
| TCGA.WX.AA44.01A | 20.21917808 | 0 | 3.621143956 | 4.584803074 | 2.164083724 | 0.267040664 | 7.001621124 | 3.648253297 | 2.414556191 | 1.175495268 | 2.923978693 |
| TCGA.WX.AA46.01A | 24.85479452 | 0 | 0.481587326 | 2.153697559 | 1.157604503 | 0.009068293 | 5.592766379 | 4.014176723 | 0.329971802 | 0.410893715 | 1.844352425 |
| TCGA.WX.AA47.01A | 18.27945205 | 1 | 2.441272163 | 2.097910758 | 2.411104745 | 0.010518795 | 4.399516111 | 2.976751301 | 2.597723174 | 0.0807487 | 2.55533317 |
| TCGA.XR.A8TC.01A | 44.02191781 | 0 | 2.275183502 | 2.285175948 | 1.387343128 | 0.46992447 | 5.958296189 | 3.750823509 | 0.27134661 | 0.738257982 | 1.945656858 |
| TCGA.XR.A8TD.01A | 33.8630137 | 0 | 3.941432372 | 3.801357531 | 4.304574442 | 0.287960217 | 6.242386646 | 5.667348589 | 1.743080539 | 2.383427355 | 2.326309938 |
| TCGA.XR.A8TE.01A | 30.4109589 | 0 | 0.980312461 | 2.338409159 | 1.638788633 | 0.036179943 | 5.071999721 | 3.386070179 | 0.336392708 | 1.079323724 | 2.710742862 |
| TCGA.XR.A8TF.01A | 22.78356164 | 1 | 2.338476403 | 3.76075775 | 2.140829049 | 0.192294991 | 6.847970172 | 1.888474999 | 0.436143715 | 0.543440136 | 1.971251374 |
| TCGA.XR.A8TG.01A | 29.52328767 | 0 | 3.48635416 | 4.746380368 | 2.859178783 | 0.280034789 | 6.136547675 | 4.779537908 | 2.168087801 | 0.832965582 | 3.329812133 |
| TCGA.YA.A8S7.01A | 13.54520548 | 1 | 4.193077484 | 4.456014033 | 3.414496609 | 0.127970208 | 8.904299469 | 5.00309054 | 2.935214883 | 0.806593248 | 3.966153723 |
| TCGA.ZS.A9CD.01A | 45.56712329 | 1 | 1.049503989 | 2.498999257 | 2.029009191 | 0.02481601 | 6.106386727 | 3.153444522 | 1.899799459 | 0.989905484 | 2.501028446 |
| TCGA.ZS.A9CE.01A | 40.8 | 0 | 2.317474213 | 2.381651445 | 1.783583997 | 0.039128783 | 6.274893355 | 3.564006206 | 0.440168568 | 0.065360213 | 1.961821705 |
| TCGA.ZS.A9CF.01A | 79.29863014 | 0 | 3.628227056 | 4.767839438 | 2.042278031 | 0.259716631 | 6.265175974 | 4.388778823 | 0.470280001 | 1.586505894 | 2.338894693 |
| TCGA.ZS.A9CG.01A | 11.2109589 | 0 | 0.523325565 | 2.076267331 | 1.030402256 | 0.018439727 | 7.332042226 | 4.998536518 | 1.180939682 | 0.390458381 | 2.730895255 |
